# Metabolomic signatures distinguish the impact of formula carbohydrates on disease outcome in a preterm piglet model of NEC

**DOI:** 10.1186/s40168-018-0498-0

**Published:** 2018-06-19

**Authors:** Lee Call, Barbara Stoll, Berthe Oosterloo, Nadim Ajami, Fariha Sheikh, Anja Wittke, Rosaline Waworuntu, Brian Berg, Joseph Petrosino, Oluyinka Olutoye, Douglas Burrin

**Affiliations:** 1 Department Pediatric Gastroenterology, Hepatology, and Nutrition, USDA-ARS Children’s Nutrition Research Center, 1100 Bates Ave, Houston, TX 77030 USA; 2 Division of Pediatric Surgery, Baylor College of Medicine, 6701 Fannin St, Suite 1210, Houston, TX 77030 USA; 3 Alkek Center for Metagenomics and Microbiome Research, Baylor College of Medicine, One Baylor Plaza, MS BCM385, Houston, TX 77030 USA; 4 Mead Johnson Pediatric Nutrition Institute, 2400 W Lloyd Expressway, Evansville, IN 47712 USA

**Keywords:** Necrotizing enterocolitis, Infant formula, Lactose, Maltodextrin, Corn syrup solids, Clostridium, Lactate

## Abstract

**Background:**

Major risk factors for necrotizing enterocolitis (NEC) include premature birth and formula feeding in the context of microbial colonization of the gastrointestinal tract. We previously showed that feeding formula composed of lactose vs. corn syrup solids protects against NEC in preterm pigs; however, the microbial and metabolic effects of these different carbohydrates used in infant formula has not been explored.

**Objective:**

Our objective was to characterize the effects of lactose- and corn syrup solid-based formulas on the metabolic and microbial profiles of preterm piglets and to determine whether unique metabolomic or microbiome signatures correlate with severity or incidence of NEC.

**Design/methods:**

Preterm piglets (103 days gestation) were given total parenteral nutrition (2 days) followed by gradual (5 days) advancement of enteral feeding of formulas matched in nutrient content but containing either lactose (LAC), corn syrup solids (CSS), or 1:1 mix (MIX). Gut contents and mucosal samples were collected and analyzed for microbial profiles by sequencing the V4 region of the 16S rRNA gene. Metabolomic profiles of cecal contents and plasma were analyzed by LC/GC mass spectrometry.

**Results:**

NEC incidence was 14, 50, and 44% in the LAC, MIX, and CSS groups, respectively. The dominant classes of bacteria were *Bacilli*, *Clostridia*, and *Gammaproteobacteria*. The number of observed OTUs was lowest in colon contents of CSS-fed pigs. CSS-based formula was associated with higher *Bacilli* and lower *Clostridium* from clusters XIVa and XI in the colon. NEC was associated with decreased *Gammaproteobacteria* in the stomach and increased *Clostridium* sensu stricto in the ileum. Plasma from NEC piglets was enriched with metabolites of purine metabolism, aromatic amino acid metabolism, and bile acids. Markers of glycolysis, e.g., lactate, were increased in the cecal contents of CSS-fed pigs and in plasma of pigs which developed NEC.

**Conclusions:**

Feeding formula containing lactose is not completely protective against NEC, yet selects for greater microbial richness associated with changes in *Bacilli* and *Clostridium* and lower NEC incidence. We conclude that feeding preterm piglets a corn syrup solid vs. lactose-based formula increases the incidence of NEC and produces distinct metabolomic signatures despite modest changes in microbiome profiles.

**Electronic supplementary material:**

The online version of this article (10.1186/s40168-018-0498-0) contains supplementary material, which is available to authorized users.

## Background

Necrotizing enterocolitis (NEC) persists as a major gastrointestinal disease among preterm infants that is characterized by a severe inflammatory response leading to necrosis of intestinal tissue and major morbidity [1, 2]. At least two dominant factors that are thought to be causally associated with NEC are bacterial colonization and enteral formula feeding. Recent evidence suggests that the preterm infant gut is not sterile [3, 4], but clearly the process of birth and especially enteral feeding sets off a cascade of physiological processes that lead to increased numbers and diversity of microbes. In animal models, NEC-like disease does not occur in animals raised germ-free [5–7], or when given broad-spectrum antibiotics [8]. In healthy, term newborn infants, gut microbial colonization can facilitate normal development of the host immune system [9–11]. However, in preterm infants, gut microbes trigger an exaggerated proinflammatory response leading to necrosis and injury because of immature intestinal innate immune function.

Microbial colonization is a critical element in NEC pathogenesis, and this has spawned widespread investigation to find specific bacteria linked to NEC [12–24]. Several reports in the past 10 years have shown associations between gut microbes and NEC using molecular, culture-independent approaches based on 16S rRNA gene sequence or metagenomic analysis [13, 25–27]. Despite these studies, no single species or uniform microbial signature has been linked to NEC; however, some taxonomic groups have been associated including *Proteobacteria* [28], *Gammaproteobacteria* [29], and *Clostridium and Staphylococcus sp.* [24] . This question has proven difficult due to several factors including the low diversity yet high inter-individual variation characteristic of the neonatal microbiota [30–32], differences in analysis methods (i.e., culture- vs. non-culture-based, different sequencing platforms) [33, 34], and the challenges of interpreting results across the various animal models and human patients, each harboring its own co-evolved microbiota [35–38]. Many of these studies also suggest that differences exist in the functional metabolic capacity of microbial communities with similar 16S rRNA gene signatures. More recently, studies have pursued a dual approach using non-targeted, metabolomic profiling of blood, urine, or stool coupled with genomic sequence analysis of fecal microbial community structure in infants with NEC [13, 25–27]. Some of these studies have shown concordance between select metabolites and 16S microbiome signatures in NEC and healthy infants.

Another dominant factor in NEC pathogenesis is diet, and numerous studies show that breast milk significantly lowers the incidence of NEC in preterm infants, in comparison to milk-based enteral formulas [39–43]. The primary carbohydrates in breast milk are lactose and oligosaccharides, whereas preterm infant formulas contain only a mixture (~ 50/50%) of lactose and maltodextrin or corn syrup solids. Corn syrup solids and maltodextrin are short (< 20) chains of glucose that are considered easier to digest than lactose in preterm neonates who often have lower lactase expression compared to term infants [44–47]. Human milk oligosaccharides have received considerable attention in support of normal microbiome development and as a protective component of human milk against NEC [48, 49]. However, the specific effects of lactose and corn syrup solids on the developing infant gut microbiome are largely unknown. Importantly, we previously showed in a preterm piglet model that feeding formulas containing lactose reduced the incidence of NEC by threefold compared to those fed maltodextrin [50]. The aim of the current study was to extend these findings and use the dual approach of microbiome and non-targeted metabolomic profiling to interrogate the relationships between dietary carbohydrates, gut microbiome community structure, and NEC pathogenesis in a preterm, neonatal piglet model. The preterm piglet is a well-established model to study NEC because it incorporates the necessary elements of prematurity and gut microbial colonization and employs relevant clinical features of enteral and parenteral nutrition in a species with close homology in physiology, intestinal anatomy, and metabolism to human preterm infants [6, 8, 51–54]. These reports have shown that the clinical and histopathologic NEC phenotype in pigs is remarkably similar to NEC in human infants.

## Results

### NEC incidence and severity highest in CSS-fed piglets

**Fig. 1:**
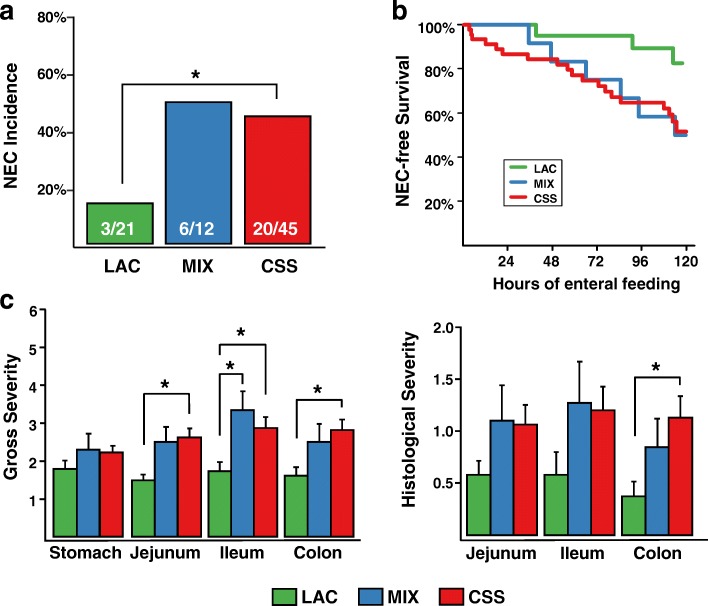
Phenotypic outcomes of the study. **a** NEC incidence within each of the three formula groups; Fisher’s exact test. **b** Kaplan-Meir analysis comparing survival time before euthanizing for NEC across the three formula groups; log-rank test. **c** Gross and histological NEC severity scores across GI regions for the three formula groups. For the gross severity, each of the GI regions is assessed at the time of dissection and assigned a score of 1–2 (healthy tissue), 3–4 (moderate inflammation), or 5–6 (pneumatosis and necrosis). For histological severity, H & E-stained tissue sections are scored as 0 (no damage), or from a range of 1 to 4 based on extent of necrosis, villus blunting, and pneumatosis. Values are mean +/− standard error of the mean; linear model with birthweight and farm as covariates and Tukey’s post hoc comparisons

The composition of the formulas fed in each dietary group is shown in Table 1.  A total of 78 pigs were included in this experiment. The incidence of NEC within the LAC, MIX, and CSS groups were 14% (3/21), 50% (6/12), and 44% (20/45), respectively (Fig. 1a). By analyzing the rates of fatal cases of NEC within each group, we found that the Kaplan-Meier survival curves of the LAC and CSS groups differ by the log-rank test (*p* = 0.027) (Fig. 1b). This also was reflected in the earlier time of death or euthanasia due to NEC in CSS and MIX pigs than LAC pigs (Table 2). The jejunum, ileum, and colon mean gross severity scores as determined at the time of death or euthanasia were lower in the LAC group compared to the CSS group; the LAC group’s ileum scores were also lower compared to the MIX group, while no difference was detected for the stomach (Fig. 1c). The severity of NEC lesions were subsequently confirmed by histological scoring using an index previously adapted for this model (Fig. 1c) [55].

**Table 1:** Ingredient and macronutrient composition of formulas containing lactose (LAC), corn syrup solids (CSS), or mixture (MIX)

	LAC	MIX	CSS
Lactose	60.0	30.0	–
Corn syrup solids	–	30.0	60.0
MCT oil	24.7	24.7	24.7
Soybean oil	18.2	18.2	18.2
High oleic (sunflower) oil	16.1	16.1	16.1
Casein protein hydrolysate	28.7	28.7	28.7
Whey protein isolate	37.3	37.3	37.3
Protein	66.0	66.0	66.0
Fat	59.0	59.0	59.0
Carbohydrate	60.0	60.0	60.0

Components are listed as g/L. All additional macrominerals, trace minerals, and vitamins are formulated to meet or exceed the requirements of neonatal piglets

**Table 2:** Body weights and gut morphometry of piglets fed formulas containing lactose (LAC), corn syrup solids (CSS), or mixture (MIX)

	LAC	MIX	CSS
Number pigs/group			
Healthy	18	6	25
NEC	3	6	20
Time of death, hr post-feeding			
Healthy	106 ± 7	121 ± 1	104 ± 6
NEC	83 ± 23	75 ± 12†	62 ± 9†
Birth weight (g)			
Healthy	934 ± 45	1011 ± 62	1066 ± 45
NEC	872 ± 28	1010 ± 108	926 ± 50†
Final weight (g)			
Healthy	1382 ± 81	1511 ± 86	1530 ± 79
NEC	1203 ± 116	1310 ± 122	1203 ± 97**†**
Weight gain (g•kg^− 1^•day^− 1^)			
Healthy	104 ± 6	99 ± 6	94 ± 6
NEC	106 ± 17	86 ± 30	61 ± 20
Proximal jejunum			
Villus height **(**μm**)**			
Healthy	467 ± 34	427 ± 39	569 ± 33
NEC	314 ± 25†	305 ± 32†	330 ± 38†
Crypt depth (μm)			
Healthy	94 ± 3	107 ± 8	102 ± 5
NEC	75 ± 8	79 ± 9†	83 ± 4†
Terminal ileum			
Villus height **(**μm**)**			
Healthy	537 ± 21	518 ± 12	634 ± 45
NEC	179 ± 52†	277 ± 55†	291 ± 46†
Crypt depth (μm)			
Healthy	114 ± 6	131 ± 12	121 ± 6
NEC	97 ± 3†	75 ± 9†	82 ± 6†
Colon			
Crypt depth (μm)			
Healthy	232 ± 14	183 ± 16	216 ± 9
NEC	314 ± 2^a^†	148 ± 5^b^	213 ± 14^c^

Values are mean ± standard error of the mean. Superscripts a, b, and c designate the results of Tukey’s post hoc test comparing across groups; groups sharing letters are not significantly different (no superscripts if no differences were detected for that row). The cross, †, identifies differences between Healthy and NEC within a group, by student’s *t* test. An alpha level of 0.05 was used for all tests

### Morphology, histology, and inflammatory gene expression

Within the CSS-fed piglets, those animals that subsequently developed NEC had an average lower body weight at birth (926 vs. 1066 g) and at euthanasia for NEC or end of study (1203 vs. 1530 g) compared to those that did not develop NEC (Table 2). There were no differences in birth weight, end of study weights, or rate of weight gain among healthy pigs in any of the formula groups. In gut morphometry, the most pronounced effect of NEC occurred in the terminal ileum villus height and crypt depth, both measures being lower in the piglets with NEC compared to healthy, regardless of which formula they received. It is important to note that although villus height does tend to increase with feedings after birth, and indeed terminal ileum measurements for all healthy pigs at the end of study are increased compared to the average at birth, 411 ± 33 μm (data not shown), the effect of NEC is to decrease villus height regardless of diet or time of death (Additional file 1). Lower measures of jejunum villus height were also observed in NEC vs. healthy piglets. We observed decreased jejunum crypt depths for NEC vs. healthy within the MIX- and CSS-fed piglets. Colonic crypt depths of NEC piglets were greatest in the LAC group vs. MIX and CSS and somewhat surprisingly were also greater compared to the healthy LAC-fed piglets.

The expression of a set of inflammatory-related genes was not significantly different across the various groups in this study. However, there was a trend for greater expression of IL-1b, IL-6, and IL-8 in samples of distal ileum tissue from NEC piglets fed CSS formula (Additional file 2).

### Impact of formula carbohydrates on preterm piglet microbiome

**Fig. 2:**
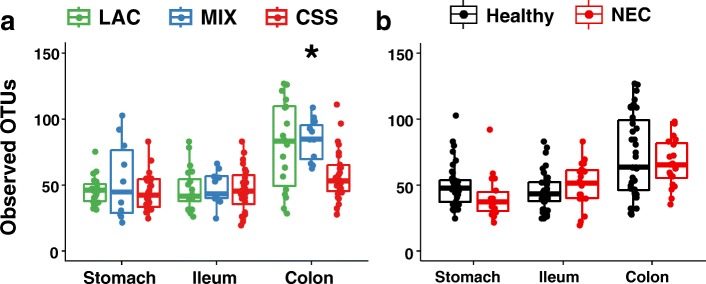
Microbiota richness in gastrointestinal contents. Counts of observed OTUs for luminal content samples from all pigs comparing the groups at each GI region in pigs by formula carbohydrate (**a**) or disease phenotype (**b**) Holm-Bonferroni adjusted Kruskal-Wallis and Mann-Whitney *U* tests

We collected samples from along the gastrointestinal tract from a subset of the pigs treated in order to assess the development of microbial communities in association with feeding these formulas. We observed the highest number of OTUs in the colons of the healthy LAC- and MIX-fed pigs, median 85 and 92 OTUs, respectively, compared to 51 in CSS-fed pigs (Fig. 2). We did not observe any differences in alpha diversity due to feeding different formulas (data not shown).

**Fig. 3:**
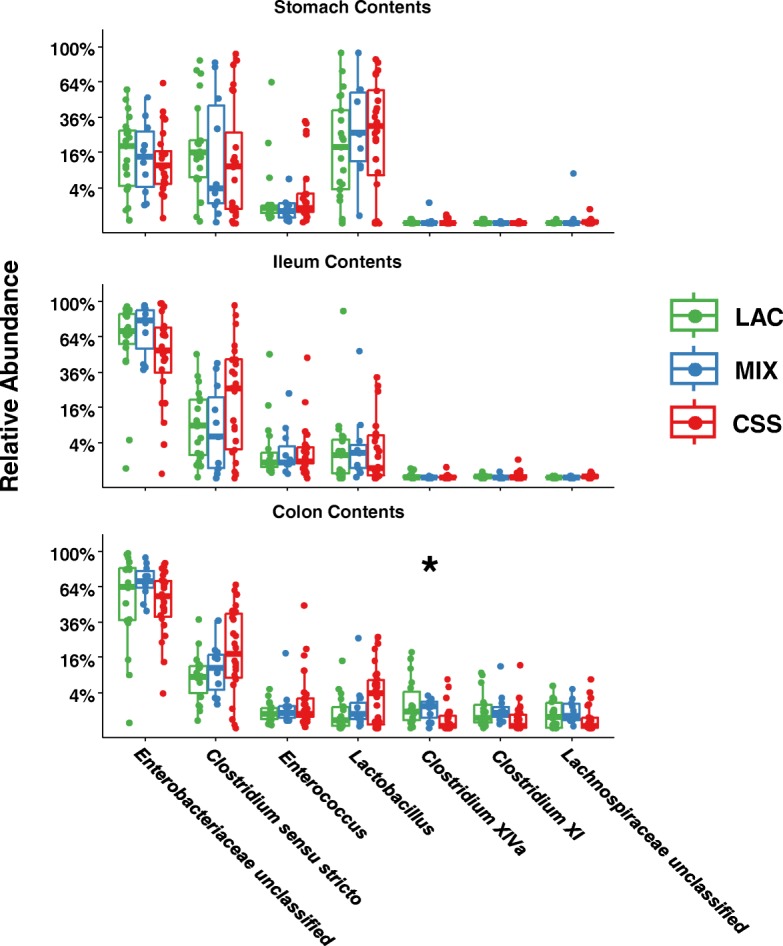
Relative abundance of the top 7 most abundant genera in gastrointestinal contents, comparing across the different formula groups. Box-and-whisker plots for genus-level comparisons of bacteria detected in piglets’ stomach, ileum, and colon luminal contents. Groups include all piglets which were fed one of the three different formulas; Holm-Bonferroni adjusted Kruskal-Wallis tests

Overall, most of the samples from both luminal contents as well as tissue mucosa were dominated by two classes of bacteria––*Gammaproteobacteria* and *Clostridia*, except for samples of stomach contents, where *Bacilli* make up a significant portion of the community. The relative abundance of Bacilli was significantly higher in the stomach (median 44%) than in either the ileum (7%) or colon contents (5%). In addition, the relative abundance of *Bacilli* in colon contents and tissue mucosa was an average of 15% higher in pigs fed CSS vs. LAC and MIX formulas (Additional files 3 and 4). The most abundant class in ileum and colon, *Gammaproteobacteria*, consisted mostly of a single group unclassified at the genus level but belonging to the family *Enterobacteriaceae*. The median relative abundance of this group was 66% across all pigs’ ileum and colon contents (Fig. 3). The largest genera in the *Clostridia* and *Bacilli* classes were *Clostridium* sensu stricto and *Lactobacillus*, respectively. The only other differences we observed across formula diets was an association with higher relative abundance of *Clostridium* from the clusters XIVa and XI in the colons of piglets receiving LAC-based formula (Fig. 3 and Additional file 5), suggesting these bacteria either benefit from lactose in the diet or are negatively impacted when corn syrup solids are the sole carbohydrate.

### Differences between healthy and NEC piglet microbiome

**Fig. 4:**
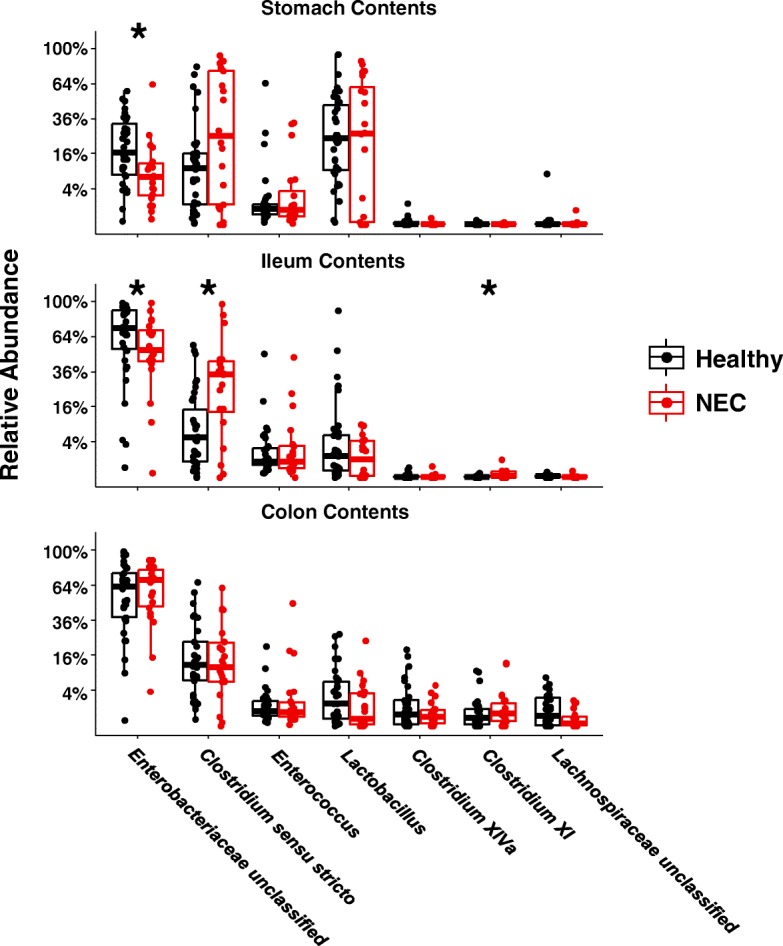
Relative abundance of the top 7 most abundant genera in gastrointestinal contents, comparing Healthy to NEC. Box-and-whisker plots for genus-level comparisons between piglets which developed NEC and those which did not develop NEC during the course of the experiment, using samples of stomach, ileum, and colon luminal contents from all piglets which were fed one of the three different formulas; Holm-Bonferroni adjusted Mann-Whitney *U* tests

We did not observe any difference in the number of observed OTUs between the healthy and NEC groups (Fig. 2). Likewise, at the class level, no differences between healthy and NEC pigs were observed in the mucosal samples (Additional file 6). However, NEC disease was associated with a lower median relative abundance of *Gammaproteobacteria* in stomach contents and ileum contents, as well as a higher relative abundance of *Clostridia* in stomach and ileum contents (Additional file 7). The decreased *Gammaproteobacteria* could be attributed at the family level to lower median relative abundance of *Enterobacteriaceae* in the stomach contents (6 vs. 19%) and ileum contents of NEC pigs (51 vs. 73% in healthy pigs) (Fig. 4). The increased *Clostridia* could be attributed at the genus level to higher median relative abundance of *Clostridium* sensu stricto in the ileum contents of NEC pigs (36 vs. 4% in healthy pigs) (Fig. 4). No major differences at the genus level were observed between healthy and NEC groups in mucosal samples (Additional file 8).

### Metabolomic profiles strongly correlate with formula carbohydrate and NEC disease

**Fig. 5:**
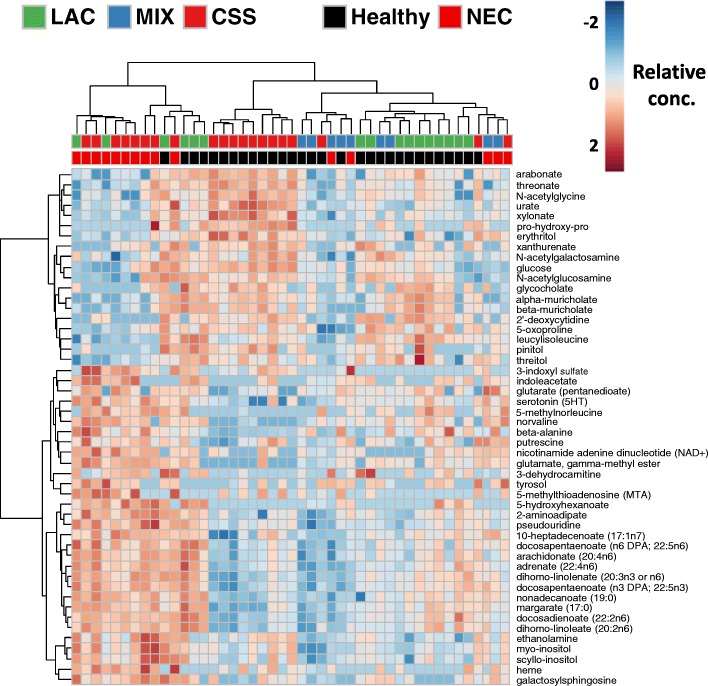
Heatmap of cecal contents metabolite profiles clustered by formula carbohydrate and disease phenotype. Shows the relative concentration of the top 50 metabolites with the largest differences between healthy and NEC piglets. All metabolites included are significantly different (FDR-adjusted *q* < 0.05) between healthy and NEC by two-way ANOVA (formula group × disease phenotype)

**Fig. 6:**
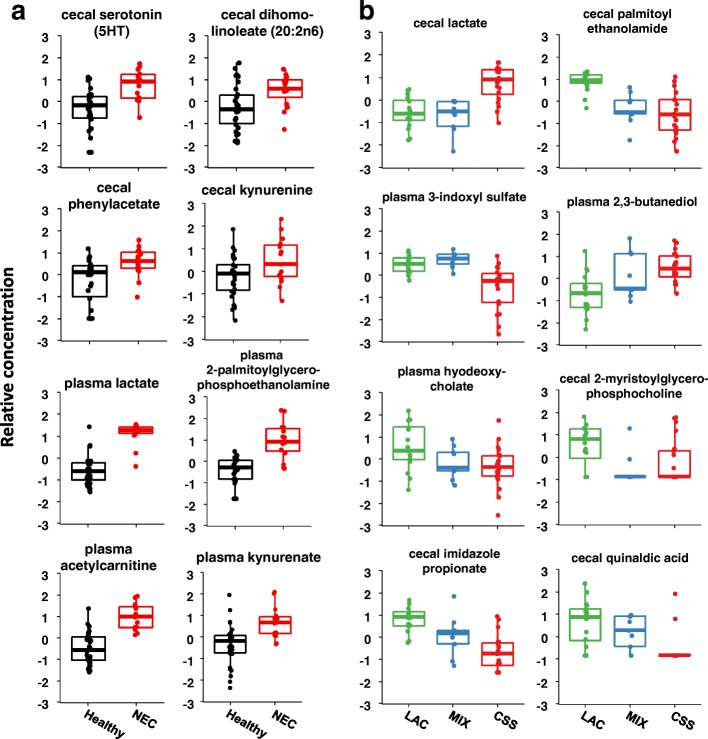
Selected plasma and cecal metabolites with differences by disease phenotype (**a**) or formula carbohydrate (**b**). Representative metabolites from the major pathways found to be differentially abundant in plasma and cecal content samples. Metabolite concentrations are shown on a mean-centered and standard deviation-scaled *y*-axis. Each metabolite shown was found to be significantly different (FDR-adjusted *q* < 0.01) either between healthy and NEC piglets or across formula groups by two-way ANOVA (formula group × disease phenotype)

We collected samples of plasma and cecal contents from a subset of the pigs treated in order to assess the metabolic profile as it relates to formula carbohydrates and NEC disease. A total of 425 unique compounds were measured in cecal content samples, and 381 were measured in plasma samples. For both sample types, we performed a two-way analysis of variance with formula carbohydrate and NEC disease phenotype as main effects. Of the 425 measured cecal metabolites, 105 were significantly different across the formula groups, while 52 differed between healthy and NEC. Of the 381 measured plasma metabolites, 4 were significantly different across the formula groups, while 205 differed between healthy and NEC. We did not detect any significant interaction between the two main effects, likely because the group sizes in the study were unequal and not powered sufficiently. Hierarchical clustering and heatmaps of plasma and cecal metabolite profiles helped identify patterns of metabolites that were enriched or depleted in the different groups of interest in this study. Several differences in cecal metabolites were observed between NEC and healthy piglets, including several aromatic amino acid metabolites, polyunsaturated fatty acids, and endocannabinoids (Figs. 5 and 6). Among the plasma metabolites that differed between NEC and healthy piglets were several involved in glycolysis, gluconeogenesis, and pyruvate metabolism pathways (e.g., glucose and lactate), fatty acid metabolism (e.g., acetylcarnitine), hydrolysis of phospholipids (e.g., 2-palmitoylglycerophosphoethanolamine), purine metabolism, aromatic amino acid metabolism, and bile acids (Additional file 9).

## Discussion

The aim of this study was to establish how dietary carbohydrate composition shapes the assembly of the gut microbiome and its metabolic products and whether these in turn determine the risk for NEC in premature piglets. Diet has a dominant influence in shaping the gut microbiome of the newborn infant given its role as a source of substrates for bacterial growth. However, the role of specific dietary components on the development of the microbiome in preterm infant is still not fully understood. Although there is great interest in the role of milk oligosaccharides in gut microbiota development, the current study focused on the simpler carbohydrates present in infant formula, lactose, and corn syrup solids. These are the primary carbohydrates in infant formula, yet there is a limited understanding about how they shape the infant gut microbial communities in the context of NEC.

The idea that maldigestion of dietary carbohydrates could lead to NEC goes back to early clinical observations as well as experiments in lactose-intolerant gnotobiotic quail showing that a lactose-based formula led to production of short-chain fatty acids by gut microbes and NEC lesions [7, 56–58]. Our previous studies comparing lactose and glucose polymers (maltodextrin) in neonatal premature piglets, which easily digest lactose but not glucose polymers, also supported the hypothesis that maldigestion can trigger NEC [50]. A few notable changes were made in the current study to increase the clinical relevance, namely continuing the provision of TPN after initiating enteral feeds and gradual increasing the feeding volumes. Importantly, these changes in the clinical protocol lowered the NEC incidence compared to our previous report (as we predicted), but the current study confirms the previous findings of a lower NEC incidence in the piglets fed a lactose- vs. corn syrup solid-based formula.

Despite the evidence that maldigestion of formula dietary carbohydrates can lead to NEC and that gut bacteria seem to mediate this effect, the mechanisms through which this occurs remain unclear. A consistent theme from the many published reports of gut microbiota in NEC is a trend of decreased diversity in NEC infants compared to healthy controls [18, 21, 23]. This is noteworthy considering the gut microbial communities in preterm infants are already very low in diversity compared to older children and adults [30, 31, 59, 60]. Likewise, we observed a similar situation in the current study––simple communities with a relatively low number of OTUs and low levels of diversity in all samples. This is also consistent with what has been shown in previous studies on neonatal piglet microbiota [61, 62]. We found no major differences in the inverse Simpson diversity index for Healthy vs. NEC pigs nor across formula groups. This is likely because there are comparatively few OTUs which dominate these young pigs, and diversity metrics are not particularly informative in conditions of just a few dominant species. On the other hand, the observed differences in the number of observed OTUs between CSS-fed piglets and the other diet groups suggest that the newborn piglet microbiota contains a number of low-abundance species which do not grow without lactose in the diet (Fig. 2). Further studies are needed to elucidate the potential significance of the loss of these low-abundance species and how it impacts the developing neonatal gut.

We designed the current study to identify changes in the infant gut microbiome driven by formula carbohydrate and/or NEC disease phenotype. In this study, all the piglets had microbiomes dominated by three classes of bacteria––*Gammaproteobacteria*, *Clostridia*, and *Bacilli*, similar to what has been reported in preterm infants [63–65]. Also consistent with studies in preterm human infants delivered by cesarean-section, our preterm piglets had similar low/undetectable levels of *Bacteroidetes* and *Actinobacteria* [32, 66–68]. The use of preterm piglets as a translational model allowed us to characterize distinct microbial communities in specific gastrointestinal regions. One of the most striking differences we observed was the increased relative abundance of *Bacilli* in stomach samples compared to samples from more distal intestinal regions. Given that we fed formula directly into the stomach, this suggests these are a major group associated with enteral nutrition, consistent with findings in stomach samples from human preterm infants [69, 70]. This relationship between available substrate and *Bacilli* may also partly explain why the CSS-fed piglets had higher amounts of *Bacilli* in their colons, where the maldigested carbohydrate may accumulate. Furthermore, several groups of facultative anaerobic *Bacilli*, e.g., *Lactobacillus*, ferment sugars to lactic acid, which may explain the association of cecal lactate with CSS-fed piglets. Overall, the dietary-associated bacterial signatures suggest that the carbohydrate composition of infant formula may affect the microbiota during the early days of development after birth. Relevant to this discussion is evidence in term infants that added dietary lactose can act as a prebiotic to alter the relative abundance of *Clostridium*, *Lactobacillus*, and other important genera [71–73]. The role of lactose in shaping the infant gut microbiota and its potential as a prebiotic in the context of low-lactase expressing preterm infants merits further investigation.

A dominant theory tested by multiple clinical studies is that specific groups of gut microbes drive NEC pathogenesis. Several groups of bacteria have been reported to correlate with NEC, yet no consensus pathogens have been confirmed. Microbial signatures associated with NEC include increased *Firmicutes* [13], for example *Clostridia* [15, 19, 20, 24], but also decreased *Firmicutes* [28, 74], such as *Clostridia* [18, 29], or *Negativicutes* [29]; increased *Gammaproteobacteria* [23, 29, 74], especially *Enterobacteriaceae* [14], for example *Klebsiella* [15], *Enterobacter* [22], or *Escherichia* [16, 20, 75]; decreased *Actinobacteria* [18], such as *Propionibacterium* [13] or *Bifidobacteria* [27]. Indeed, one recent study using a metagenomics approach providing strain-level resolution found that even within an outbreak of NEC at a single NICU and despite harboring similar NEC-associated species, no strains were shared among affected infants [17]. Despite the technical limitations in comparing results across studies, it is obvious that it remains difficult to agree on what might be the core NEC pathogenic taxa. One particularly intriguing idea put forth by some researchers is that although there may be no specific NEC pathogens, perhaps the absence of keystone species during early development may strongly influence the risk of disease. Likely candidates such as *Bifidobacteria* have been the focus of much research [27, 76]. But there may be other bacteria which can exert protective influence on the fragile infant. Our observation of higher relative abundance of *Clostridium* from clusters XIVa and XI in lactose vs. CSS-fed pigs could be another candidate, as species in these genus have been shown to be capable of inducing immune-modulating regulatory T cells [10]. In the current study using preterm piglets, we found significant differences between NEC and healthy in the relative abundance of bacteria within classes which have previously been reported to be associated with NEC such as *Clostridia* and *Gammaproteobacteria*. In particular, an NEC-associated increase in ileum *Clostridium* sensu stricto genus which includes the species *Clostridium butyricum* and *perfringens* previously linked to NEC [7, 15, 19, 24]. However, consistent with many reports found in the literature on the NEC microbiome, our results support the conclusion that the presence or absence of any specific taxa does not seem to drive NEC pathogenesis.

In contrast to the large number of studies focused on characterizing the preterm infant’s gut microbiome, fewer have used a metabolomic approach to identify NEC-associated metabolites, despite the promise of providing greater mechanistic insight into disease pathogenesis and potential for identifying clinical biomarkers. One of the first such studies looked at urinary metabolite profiles and identified changes in alanine, pyridoxine, and histidine [13]. Another analyzed serum of infants with NEC and found a network of metabolites linked to upregulated interleukin-1β, including many amino acids [26]. The only published report of metabolomic analysis comparing stool samples from healthy and NEC infants reported five metabolites positively correlated with NEC diagnosis, and these related to the C21-steroid hormone biosynthesis, linoleate metabolism, and leukotriene metabolism and prostaglandin formation from arachidonate pathways [27].

One of the strengths of our study is the combined power of this dual microbiome and metabolomics analysis on paired samples of plasma and cecal contents from the same subjects. Although we found few differences in the gut microbiome profiles associated NEC disease phenotype, there were significant differences in many metabolites between NEC and healthy piglets. Notably, the plasma samples from all NEC pigs, regardless of which formula they received, were enriched with numerous phospholipid metabolites that we suggest are indicative of platelet activation that occurs during disease pathogenesis [77, 78]. Another consistent finding was increased lactate in both plasma of all NEC pigs and cecal contents of CSS-fed pigs. This pattern was intriguing and may signal hypoxic metabolism in the plasma of the host during NEC pathogenesis, whereas increased cecal lactate may be driven by fermentation of corn syrup solids by *Lactobacillus* as mentioned above. Lactic acidosis, especially the D-isomer produced by bacteria, has been reported in infants with NEC and in conditions of bacterial overgrowth with short-bowel syndrome [79–82]. D-lactic acidosis has been linked to encephalopathy and other toxicities [83]. Among those compounds increased in the cecal contents of NEC piglets, we observed an increase in some products of linoleate, arachidonate, and other omega-6 fatty acid metabolism, similar to those reported by Stewart et al*.* The plasma samples from NEC piglets also had increased levels of many of the amino acids reported by Wilcock et al., including ornithine, glycine, serine, and phenylalanine. Among the cecal metabolites in NEC pigs were several metabolites of aromatic amino acids known to be involved in inflammation and metabolized by both the host and microbes, such as serotonin, phenylacetate, and kynurenine [84, 85].

## Conclusion

In summary, we conclude that dietary carbohydrate present in formula can cause specific microbiome changes, yet the association of these changes with disease phenotype was subtle in this study. However, our results suggest that the presence of corn syrup solids, an ingredient that represents over half of the carbohydrate in commercial preterm infant formulas, can lead to major changes in the metabolome that may predispose or even trigger onset of NEC pathology before substantial changes occur in the gut microbiome community. Our results highlight the importance of lactose, the major sugar in human milk, and how it may function as a prebiotic and select for greater microbiome diversity in the developing perinatal gut. More in-depth metagenomic metabolomic analysis is warranted to identify the specific species selected by these dietary carbohydrates and how their fermentation products impact host mucosal function in the developing neonate. Our findings also suggest that the type of carbohydrate in the diet may yield markedly different metabolomic profiles despite relative modest changes in the microbiome community structure.

## Methods

### Pig feeding experiment

The animal protocol was approved by the Animal Care and Use Committee of Baylor College of Medicine and was conducted in accordance with National Institutes of Health guidelines. Mixed-breed pregnant sows were brought to our facility a week before surgery to acclimate. Piglets were delivered by cesarean section at 103 days gestation (full term = 115 days) as described previously [53]. Following resuscitation and within a few hours after birth, piglets underwent surgery for the placement of a jugular venous catheter and an orogastric tube. Piglets received total parenteral nutrition via jugular catheter for the first 48 h, at which time enteral feeding via orogastric tube was initiated. Passive immunity was provided to the piglets with injections of sow plasma (collected at time of cesarean section) at 6, 12, and 24 h postnatal. The piglets also received injections (buprenorphine, 0.01 mg/kg per 12 h) for pain and were monitored for blood oxygen saturation level (SpO2) and rectal temperature at least twice daily. Over the next 5 days the piglets were fed enterally via orogastric tube every 3 h. The volume of enteral formula fed to the piglets increased each day based on their body weight as measured every other day. As the daily total volume of enteral nutrition increased, the volume of TPN was decreased (see Additional file 10). During the course of the experiment, and especially during the last 5 days when the piglets were receiving enteral formula, signs of NEC were monitored carefully. Initial signs of NEC, e.g., diarrhea and lethargy, were recorded when observed and piglets continued to receive enteral feeds. Upon observation of advanced clinical symptoms of NEC, e.g., abdominal distension, persistent recumbence, and rectal temperature > 40°°C, the piglets were administered subcutaneous buprenorphine (0.01 mg/kg every 12 h while symptoms persist), their SpO2 was monitored every 3 h, and their enteral feeds continued. Upon observation of these advanced clinical symptoms of NEC *and* respiratory distress, cyanosis, labored breathing, or persistent SpO2 < 80%, piglets were immediately euthanized with beuthanasia solution. At the end of the study period (7 days total), all remaining piglets were euthanized and their organs were measured and sampled. At the time of sacrifice, a NEC severity score ranging from 1 to 6 was assigned to the major GI regions (stomach, jejunum, ileum, and colon) of each piglet based on the presence of gross pathological signs of NEC disease. A score of 1–2 indicates normal, healthy tissue, 3–4 indicates moderate redness and inflammation, and 5–6 represents pneumatosis and necrosis.

### Sample collection and histology

Samples were collected from the contents of the stomach, distal ileum, and proximal colon (cecum). Tissue mucosal samples were also collected from the mucosa of the ileum and colon. Tissue samples for histology were fixed in 10% formalin then transferred to 70% ethanol before being embedded in paraffin, sectioned, and stained with hematoxylin and eosin. For each piglet, sections representing the major GI regions (stomach, jejunum, ileum, and colon) were analyzed under a light microscope by a blinded observer and assigned a histological NEC severity score. The scores ranged from a value of 0 (no damage), or a range of 1 to 4 based on extent of necrosis, villus blunting, and pneumatosis, as described previously [55].

### Gene expression

The expression of several inflammation-related genes was assessed in a subset (*n* = LAC-healthy:14, LAC-NEC:2, MIX-healthy:5, MIX-NEC:5, CSS-healthy:9, CSS-NEC:5) of the pigs treated in this study. RNA was extracted from samples of distal ileum using the RNEasy Mini kit (Qiagen). Reverse transcription using the High Capacity cDNA Reverse Transcription (Applied Biosystems) was followed by real-time PCR using Power SYBR Green (ThermoFisher) performed on a Bio-Rad CFX96 machine. The relative expression levels were determined using the delta-delta Ct method.

### Microbiome analysis

For microbiome analysis, we used methods adapted from those used in the NIH Human Microbiome Project [86]. A total of 279 samples from 58 pigs (*n* = LAC-healthy:18, LAC-NEC:3, MIX-healthy:6, MIX-NEC:5, CSS-healthy:12, CSS-NEC:14) were collected and processed for amplicon sequencing of the V4 region of the 16S ribosomal RNA gene. All samples were processed in two separate batches by the Alkek Center for Metagenomics and Microbiome Research at Baylor College of Medicine. The PowerSoil DNA isolation kit (Mo Bio Laboratories) was used to extract genomic DNA, followed by PCR amplification of the V4 region of the bacterial 16S rRNA gene and sequencing using the 2 × 250 paired-end protocol on an Illumina MiSeq. For technical processes, a set of quality checks are included in all steps from sample intake to data analysis. Projects and samples are inspected as they arrive in the laboratory, and the appropriate paperwork (metadata capture form and chain of custody form) is cross-referenced with the samples received. Samples are logged in and immediately stored in temperature controlled systems until the next step of processing. For extraction, a set of positive and negative controls are included in every processing plate and are used to track extraction efficiencies and cross-contamination. For positive and negative control, there is confirmation of the presence or absence, respectively, of amplicon band at the expected molecular weight after PCR amplification. Positive and negative controls are composed of previously characterized samples and non-template mock samples, respectively. For amplification, a control DNA (bacterial isolate) is included in every amplification plate in addition to a non-template blank. These sets of controls are carried into sequencing and data analysis. Minimum values are required for the following parameters, Q30-Score: > 75%, cluster density: > 700 K/mm^2^, clusters pass filter: > 75%, and estimated yield: >6GB. Sequence analysis definitions are > 1000 reads, 99% of reads mapping to reference, and < 1000 raw reads, 500 mapped reads for positive and negative controls, respectively. Negative controls were included in one of the two batches and sequence results confirmed the absence of contamination based on findings of 457 raw sequences, 48 mapped sequences, and 17 unmapped sequences.

For our 279 samples, we received back 12,026,340 reads from the sequencer. The raw sequence data was analyzed using the mothur software package (v.1.39.0) [87]. Sequence reads having 0 ambiguous bases, max length of 260, and max homopolymer length of 8 were retained. Reads were aligned to the V4 region of the SILVA database (v.123). Chimeras were removed using vsearch using the default parameters and singleton reads were also removed. Reads were then classified using the RDP training set (v.14), and OTUs were formed by clustering at the 0.03 level using opticlust [88]. Subsequent analysis using the mothur-outputted biom file was performed in R using, among others, the phyloseq package [89]. A total of 273 samples passed quality filtering and read processing. The average number of sequences per sample was 28,010, and the standard deviation was 9880. After calculating bacterial community richness (observed OTU counts) and alpha diversity (inverse Simpson’s index), the data were subsampled to the minimum sequence count by sample type, e.g., contents (11,790) or mucosal tissue (5315), and the OTU abundances were transformed to relative proportions by dividing each by the total counts per sample. All OTUs assigned to a given taxonomic ranking, e.g., class or genus, were combined together and subsequent comparisons were made using these taxonomic groupings.

### Metabolomic analysis

Metabolomic analysis of plasma and cecal contents were analyzed in collaboration with Metabolon, Inc. (Durham, NC). A total of 90 samples, 45 of each sample type, from 45 pigs (*n* = LAC-healthy:14, LAC-NEC:2, MIX-healthy:5, MIX-NEC:4, CSS-healthy:11, CSS-NEC:9) were collected and processed for non-targeted metabolomics profiling (Additional file 11). Briefly, small molecules were extracted from the samples using a combination of aqueous and organic solvents. Residual organic solvent was removed using a TurboVap (Zymark), and the extracts were lyophilized, then equal amounts were analyzed by GC/MS and UPLC-MS/MS in parallel. For UPLC-MS/MS, extracts were analyzed under both acidic and basic conditions using an ACQUITY (Waters) UPLC and an LTQ (Thermo-Finnigan) mass spectrometer. For GC/MS, extracts were derivatized with bistrimethyl-silyl-triflouroacetamide, then analyzed using a Trace DSQ (Thermo-Finnigan) mass spectrometer. Compounds were identified by comparison of the raw data with Metabolon’s curated library of standards. The values for compounds in the cecal contents samples were normalized by the dry mass of the sample. Missing values were imputed with half the compound minimum. Absolute compound intensity values were used to calculate fold differences between the groups, while for all other analyses, the values were transformed using the generalized log transformation then mean-centered and scaled by the standard deviation.

### Statistical analysis

All results were analyzed using the R software program for statistics and graphical presentation [90]. The following statistical techniques were used to determine significance of a given observation: For NEC incidence Fisher’s exact test was used. For the survival curve analysis, a log-rank test was used. For severity measurements, a linear model with birthweight and farm as covariates and Tukey’s post hoc comparisons was used. For relative gene expression, pairwise Mann-Whitney *U* tests were used. In analyzing relative abundance of bacterial classes and genera, a Kruskal-Wallis rank sum test was used to test for differences across the three formula groups, a Mann-Whitney *U* test was used to test for differences between healthy and NEC, and to control for significance tests involving multiple hypotheses, the Holm-Bonferroni correction was applied and significance was assessed at alpha < 0.05. For the analysis of the metabolomics profiles, a two-way analysis of variance (ANOVA) was used with formula group (LAC, MIX, and CSS) and disease phenotype (NEC and Healthy) as main effects. We initially performed analysis to test for an interaction and when the interaction was not significant main effects were reported. The Benjamini-Hochberg false discovery rate method, with *q* < 0.01, was used to prevent inflation of the type I error rate.

## Additional files

Additional file 1: Plot of terminal ileum villus height in individual pigs from newborn, healthy, and NEC groups over the entire study period. (PDF 80 kb)

Additional file 2: Gene expression of major inflammatory cytokines. Samples from distal ileum tissue from a subset of the pigs in this study were assessed for expression of inflammation-related genes using real-time reverse-transcription PCR (RT-PCR). Holm-Bonferroni adjusted Mann-Whitney *U* test. (PDF 64 kb)

Additional file 3: Relative abundance of the top 4 most abundant classes in intestinal mucosal tissue, comparing across the different formula groups. Box-and-whisker plots for class-level comparisons of bacteria detected in piglets’ ileum and colon mucosal tissue. Groups include all piglets which were fed one of the three different formulas; Holm-Bonferroni adjusted Kruskal-Wallis tests. (PDF 140 kb)

Additional file 4: Relative abundance of the top 4 most abundant classes in gastrointestinal contents, comparing across the different formula groups. Box-and-whisker plots for class-level comparisons of bacteria detected in piglets’ stomach, ileum, and colon luminal contents. Groups include all piglets which were fed one of the three different formulas; Holm-Bonferroni adjusted Kruskal-Wallis tests. (PDF 162 kb)

Additional file 5: Relative abundance of the seven most abundant genera in intestinal mucosal tissue, comparing across the different formula groups. Box-and-whisker plots for genus-level comparisons of bacteria detected in piglets’ ileum and colon mucosal tissue. Groups include all piglets which were fed one of the three different formulas; Holm-Bonferroni adjusted Kruskal-Wallis tests. (PDF 185 kb)

Additional file 6: Relative abundance of the four most abundant classes in intestinal mucosal tissue, comparing Healthy to NEC. Box-and-whisker plots for class-level comparisons between piglets which developed NEC and those which did not develop NEC during the course of the experiment, using samples of ileum and colon mucosal tissue from all piglets which were fed one of the three different formulas; Holm-Bonferroni adjusted Mann-Whitney *U* tests. (PDF 138 kb)

Additional file 7: Relative abundance of the four most abundant classes in gastrointestinal contents, comparing Healthy to NEC. Box-and-whisker plots for class-level comparisons between piglets which developed NEC and those which did not develop NEC during the course of the experiment, using samples of stomach, ileum, and colon luminal contents from all piglets which were fed one of the three different formulas; Holm-Bonferroni adjusted Mann-Whitney *U* tests. (PDF 159 kb)

Additional file 8: Relative abundance of the seven most abundant genera in intestinal mucosal tissue, comparing Healthy to NEC. Box-and-whisker plots for genus-level comparisons between piglets which developed NEC and those which did not develop NEC during the course 41 of the experiment, using samples of ileum and colon mucosal tissue from all piglets which were fed one of the three different formulas; Holm-Bonferroni adjusted Mann-Whitney *U* tests. (PDF 183 kb)

Additional file 9: Heatmap of plasma metabolite profiles clustered by formula carbohydrate and disease phenotype. Shows the relative concentration of the top 50 metabolites with the largest differences between healthy and NEC piglets. All metabolites included are significantly different (FDR-adjusted *q* < 0.05) between healthy and NEC by two-way ANOVA (formula group × disease phenotype). (PDF 117 kb)

Additional file 10: Overview of the study design and experimental groups. Diagram of the timing of major components of the experiment, including introduction and weaning of total parenteral nutrition (TPN), introduction and ramp-up of enteral formula feeding, and the time of euthanasia and tissue collection for the three experimental groups. (PDF 86 kb)

Additional file 11: Description of metabolomic platform, analysis pipeline, and compound identification. (DOCX 16 kb)

Additional file 12: Data, metadata files, and R scripts used to generate the figures. The Rmarkdown_data_metadata.zip is a compressed folder containing the Rmarkdown file, which details the analysis scripts (in both RMD and HTML format). The batch file used to process the sequence data in mothur is provided. Microbiome data is included here as a BIOM-formatted table. All other data (metabolomics and cytokine expression) along with metadata are CSV files. (ZIP 992 kb)

## Abbreviations

CSS: Corn syrup solids
LAC: Lactose
NEC: Necrotizing enterocolitis
OTU: Operational taxonomic unit
