# Supplementary figures and images for: Metabolomic signatures distinguish the impact of formula carbohydrates on disease outcome in a preterm piglet model of NEC
Source: Microbiome. 2018 Jun 19;6:111. doi: 10.1186/s40168-018-0498-0 (PMC6009052; doi:10.1186/s40168-018-0498-0)

## Additional File 1

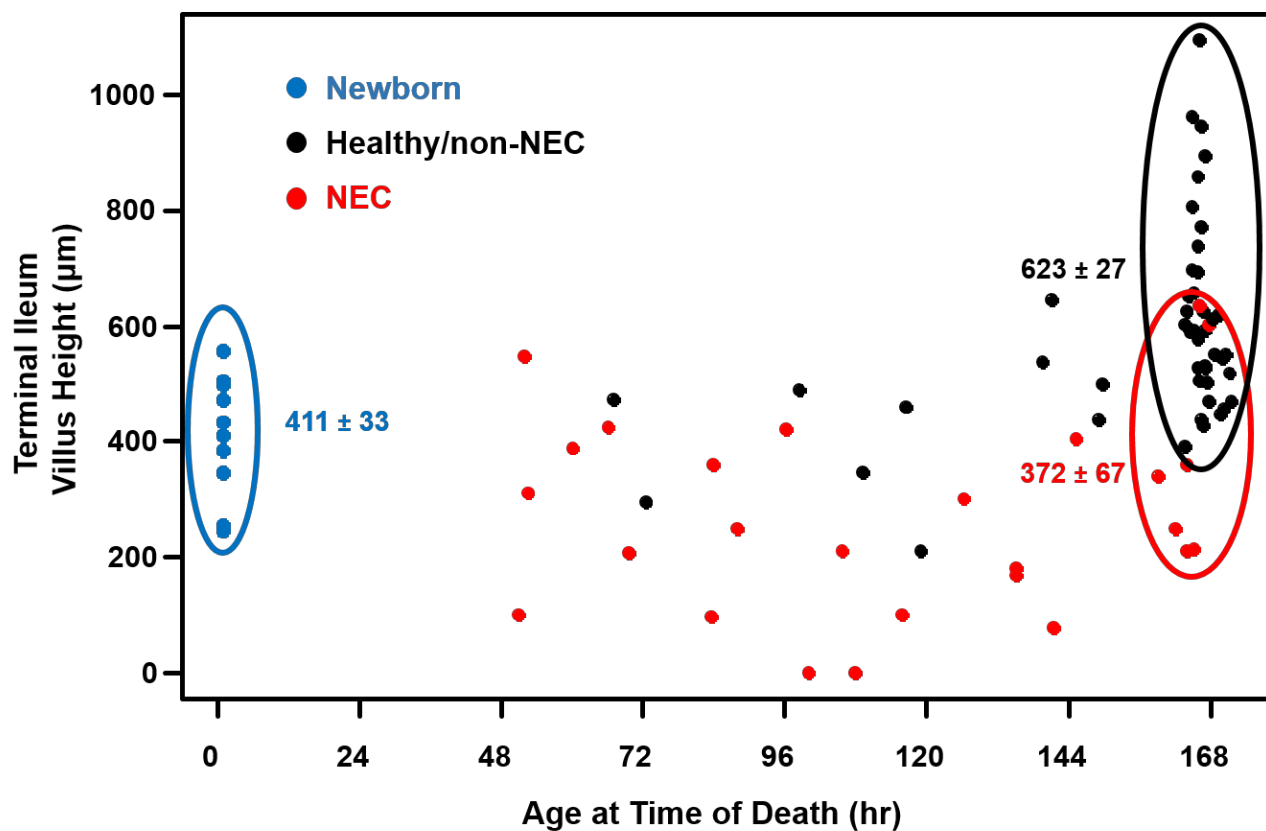

Supplement: Supplementary file 1 — Plot of terminal ileum villus height in individual pigs from newborn, healthy, and NEC groups over the entire study period. (PDF 80 kb) [file 40168_2018_498_MOESM1_ESM.pdf]

Additional File 2

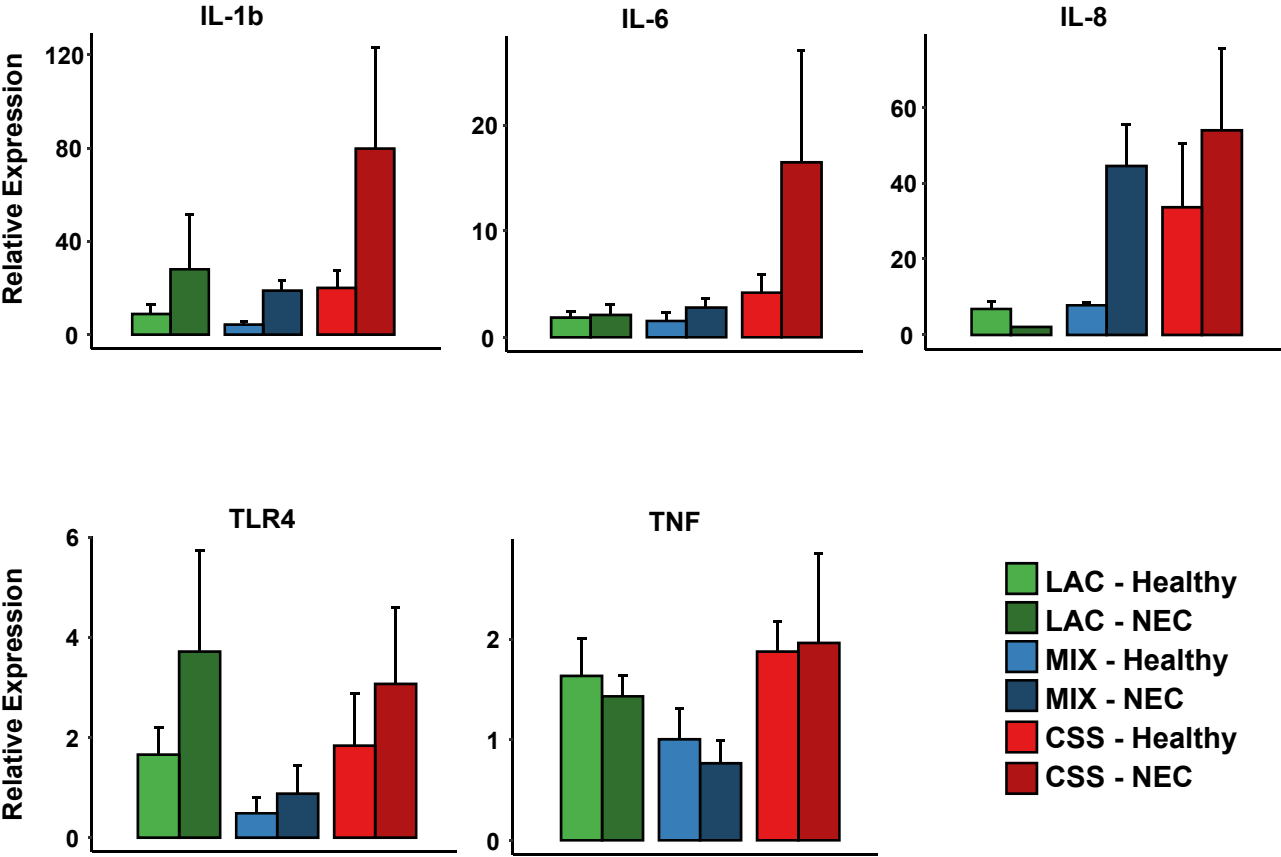

Supplement: Supplementary file 2 — Gene expression of major inflammatory cytokines. Samples from distal ileum tissue from a subset of the pigs in this study were assessed for expression of inflammation-related genes using real-time reverse-transcription PCR (RT-PCR). Holm-Bonferroni adjusted Mann-Whitney U test. (PDF 64 kb) [file 40168_2018_498_MOESM2_ESM.pdf]

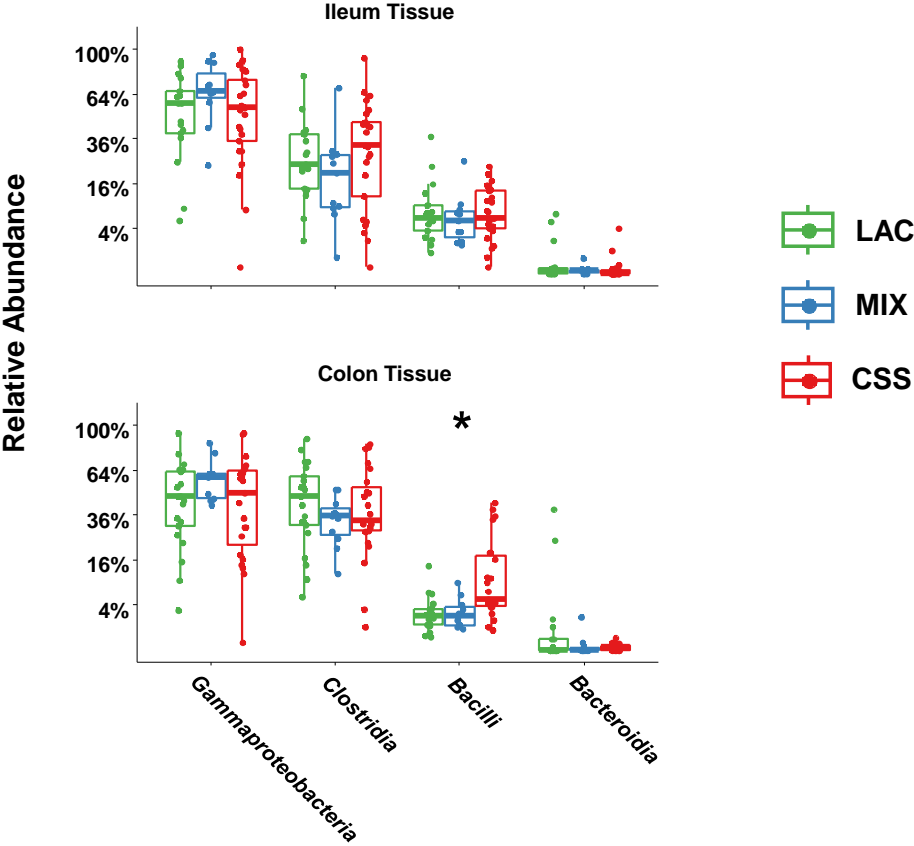

Supplement: Supplementary file 3 — Relative abundance of the top 4 most abundant classes in intestinal mucosal tissue, comparing across the different formula groups. Box-and-whisker plots for class-level comparisons of bacteria detected in piglets’ ileum and colon mucosal tissue. Groups include all piglets which were fed one of the three different formulas; Holm-Bonferroni adjusted Kruskal-Wallis tests. (PDF 140 kb) [file 40168_2018_498_MOESM3_ESM.pdf]

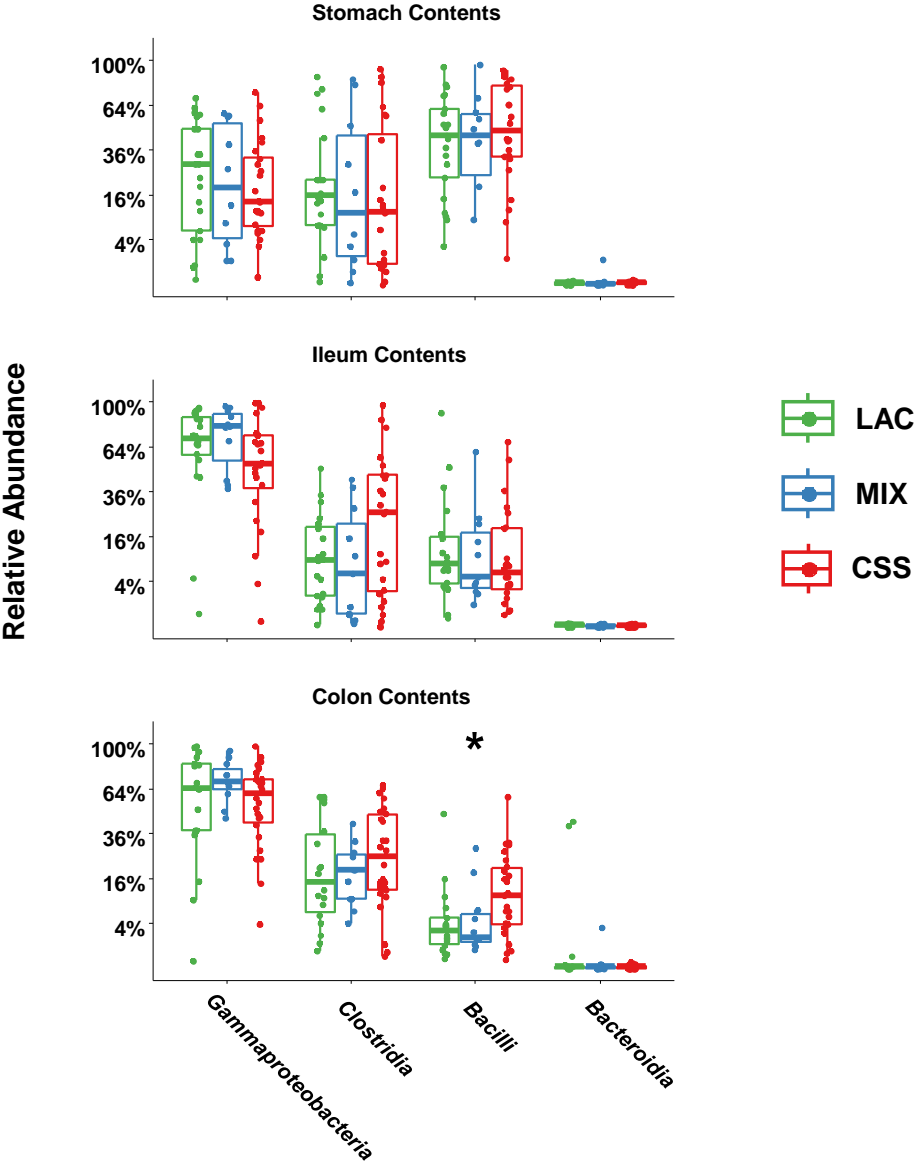

Supplement: Supplementary file 4 — Relative abundance of the top 4 most abundant classes in gastrointestinal contents, comparing across the different formula groups. Box-and-whisker plots for class-level comparisons of bacteria detected in piglets’ stomach, ileum, and colon luminal contents. Groups include all piglets which were fed one of the three different formulas; Holm-Bonferroni adjusted Kruskal-Wallis tests. (PDF 162 kb) [file 40168_2018_498_MOESM4_ESM.pdf]

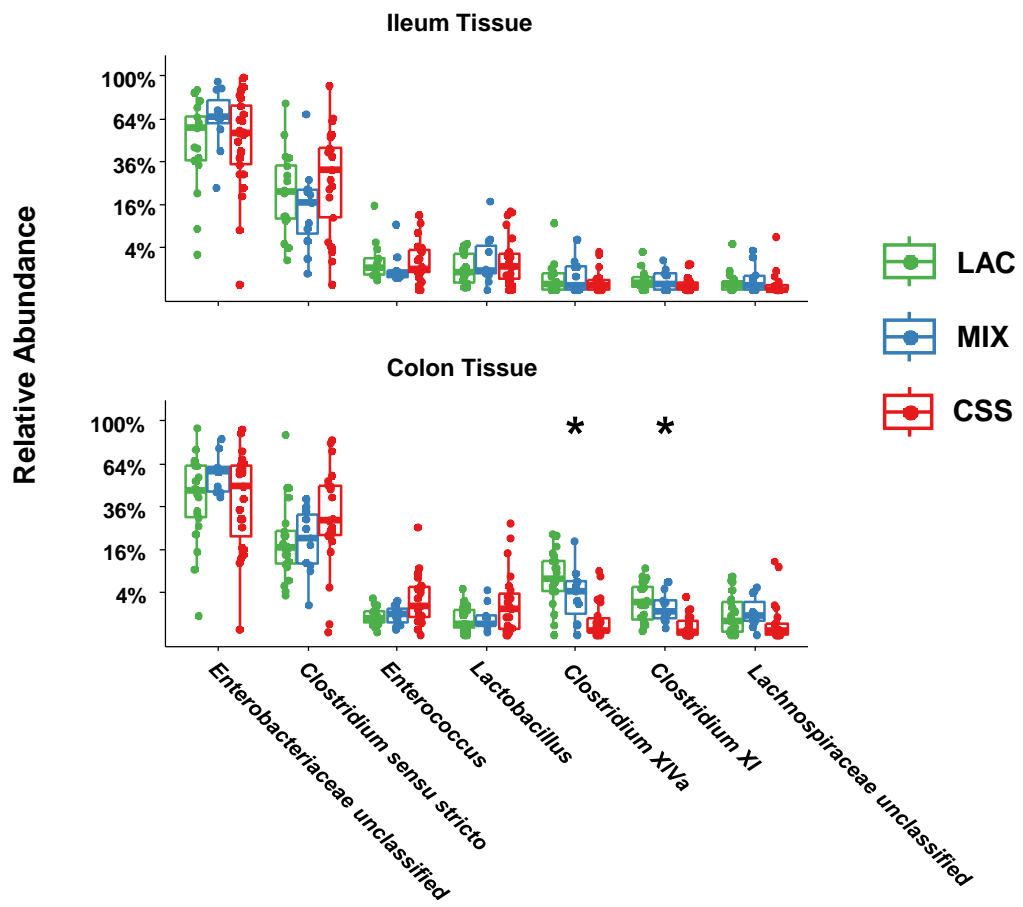

Supplement: Supplementary file 5 — Relative abundance of the seven most abundant genera in intestinal mucosal tissue, comparing across the different formula groups. Box-and-whisker plots for genus-level comparisons of bacteria detected in piglets’ ileum and colon mucosal tissue. Groups include all piglets which were fed one of the three different formulas; Holm-Bonferroni adjusted Kruskal-Wallis tests. (PDF 185 kb) [file 40168_2018_498_MOESM5_ESM.pdf]

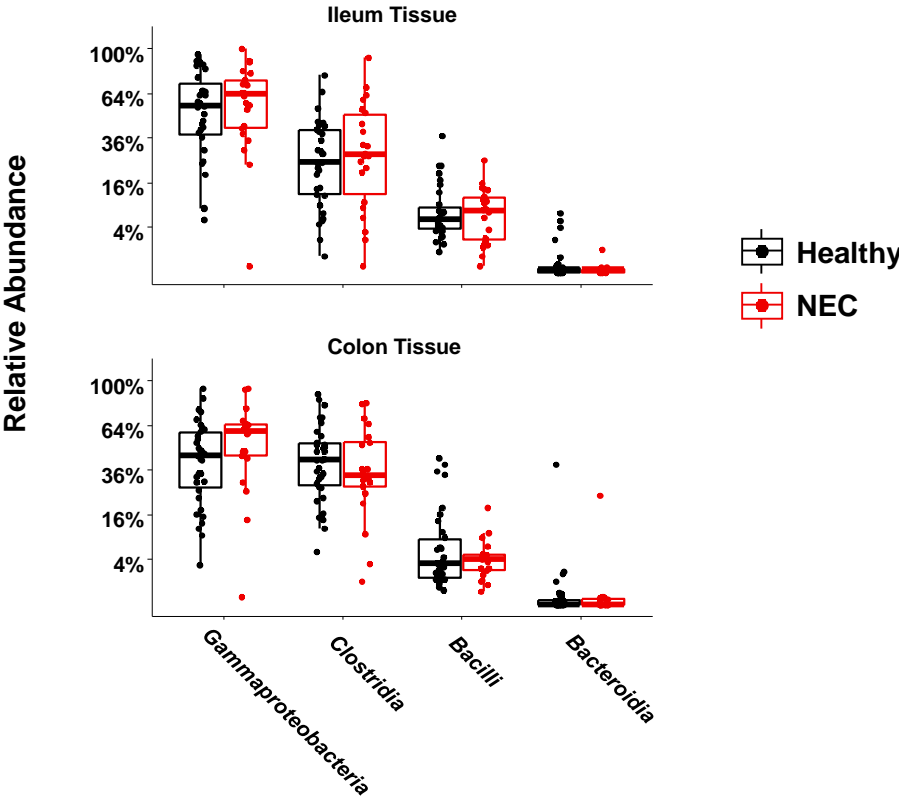

Supplement: Supplementary file 6 — Relative abundance of the four most abundant classes in intestinal mucosal tissue, comparing Healthy to NEC. Box-and-whisker plots for class-level comparisons between piglets which developed NEC and those which did not develop NEC during the course of the experiment, using samples of ileum and colon mucosal tissue from all piglets which were fed one of the three different formulas; Holm-Bonferroni adjusted Mann-Whitney U tests. (PDF 138 kb) [file 40168_2018_498_MOESM6_ESM.pdf]

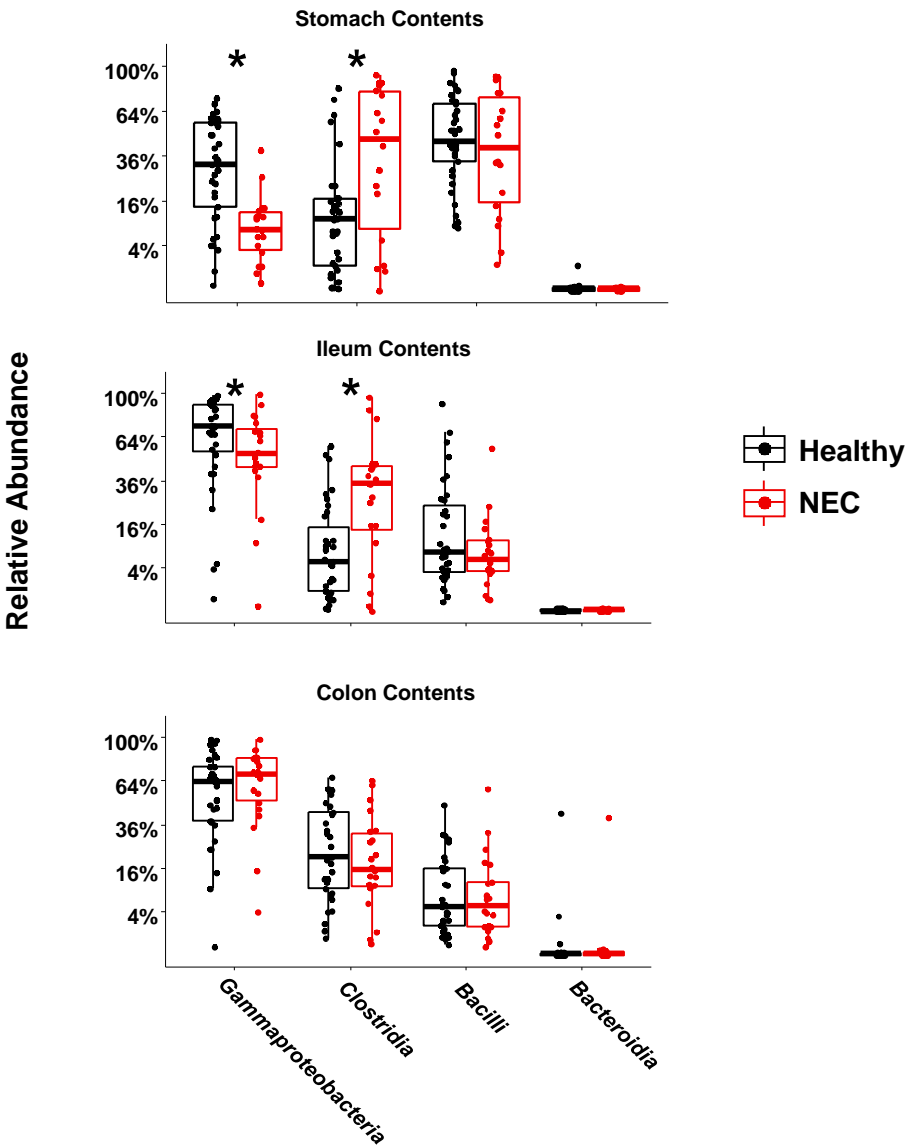

Supplement: Supplementary file 7 — Relative abundance of the four most abundant classes in gastrointestinal contents, comparing Healthy to NEC. Box-and-whisker plots for class-level comparisons between piglets which developed NEC and those which did not develop NEC during the course of the experiment, using samples of stomach, ileum, and colon luminal contents from all piglets which were fed one of the three different formulas; Holm-Bonferroni adjusted Mann-Whitney U tests. (PDF 159 kb) [file 40168_2018_498_MOESM7_ESM.pdf]

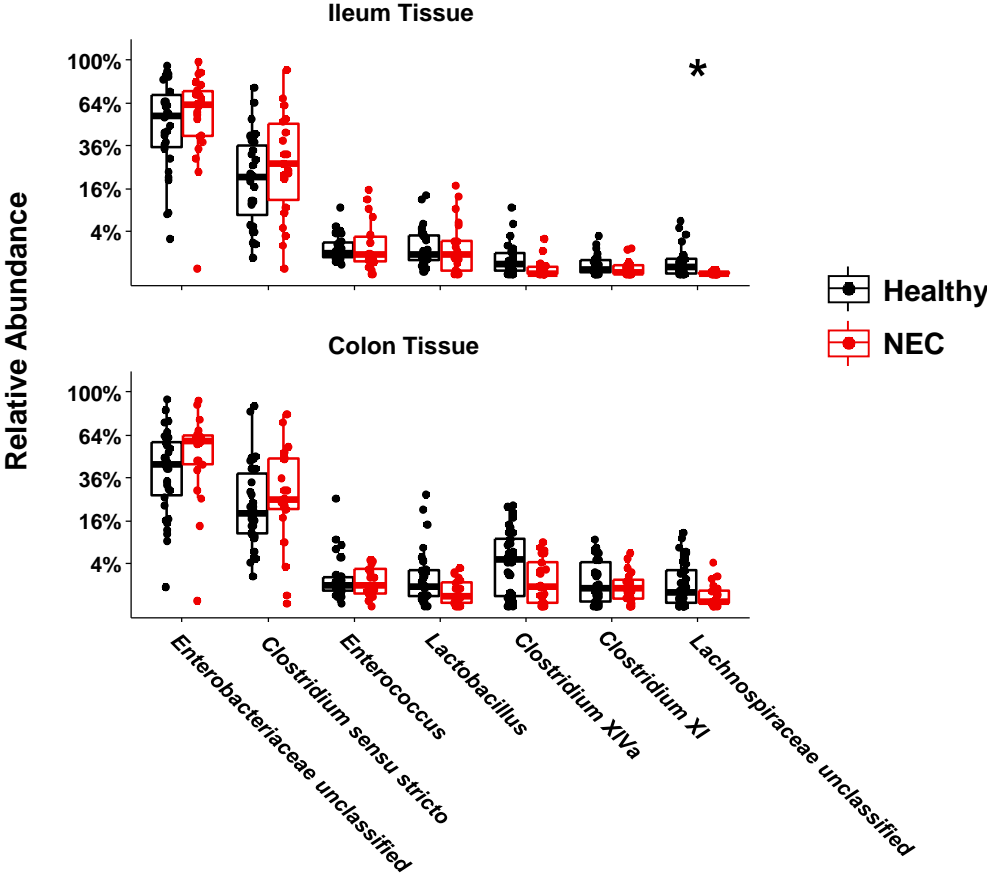

Supplement: Supplementary file 8 — Relative abundance of the seven most abundant genera in intestinal mucosal tissue, comparing Healthy to NEC. Box-and-whisker plots for genus-level comparisons between piglets which developed NEC and those which did not develop NEC during the course 41 of the experiment, using samples of ileum and colon mucosal tissue from all piglets which were fed one of the three different formulas; Holm-Bonferroni adjusted Mann-Whitney U tests. (PDF 183 kb) [file 40168_2018_498_MOESM8_ESM.pdf]

Additional File 9

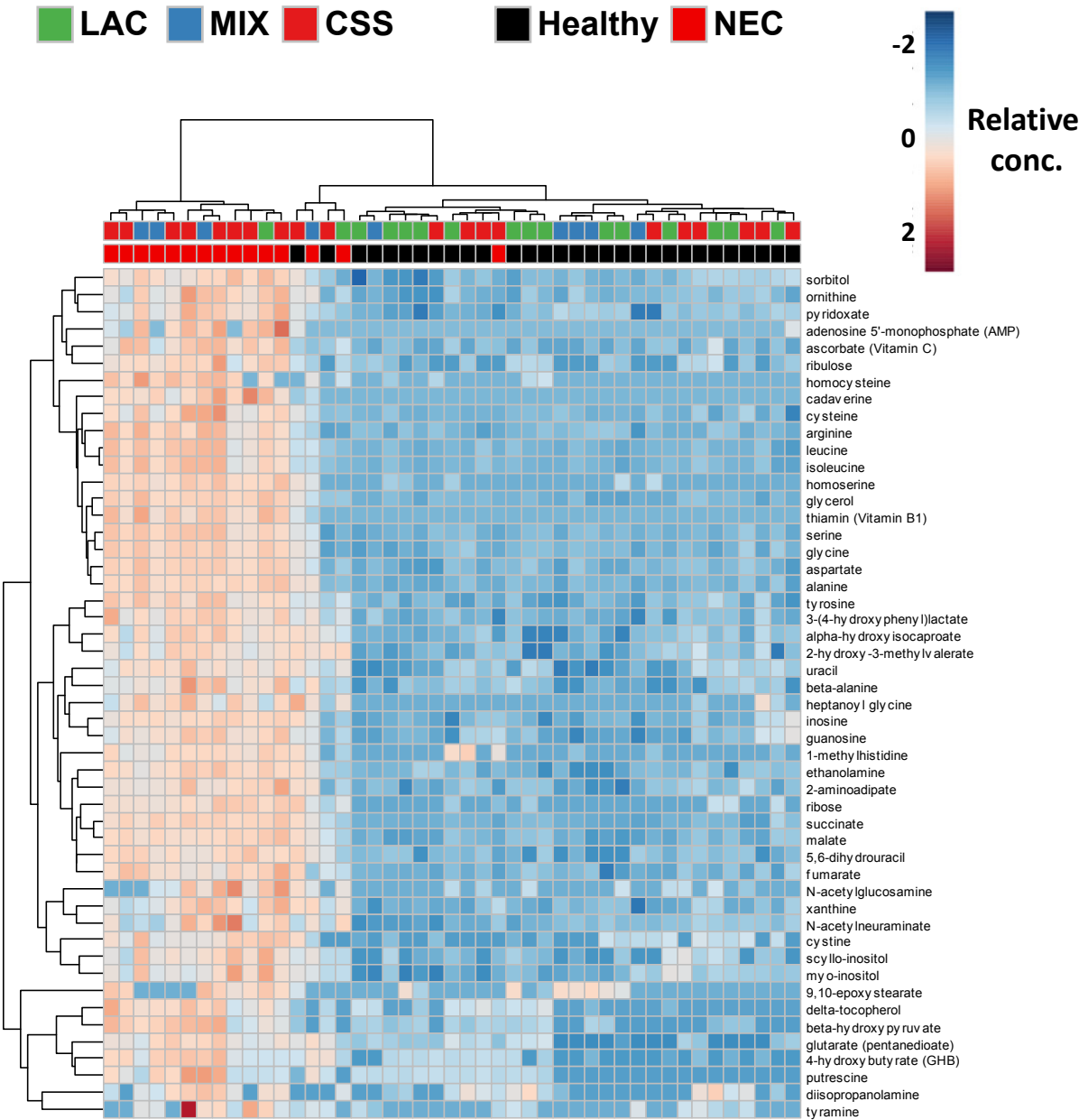

Supplement: Supplementary file 9 — Heatmap of plasma metabolite profiles clustered by formula carbohydrate and disease phenotype. Shows the relative concentration of the top 50 metabolites with the largest differences between healthy and NEC piglets. All metabolites included are significantly different (FDR-adjusted q < 0.05) between healthy and NEC by two-way ANOVA (formula group × disease phenotype). (PDF 117 kb) [file 40168_2018_498_MOESM9_ESM.pdf]

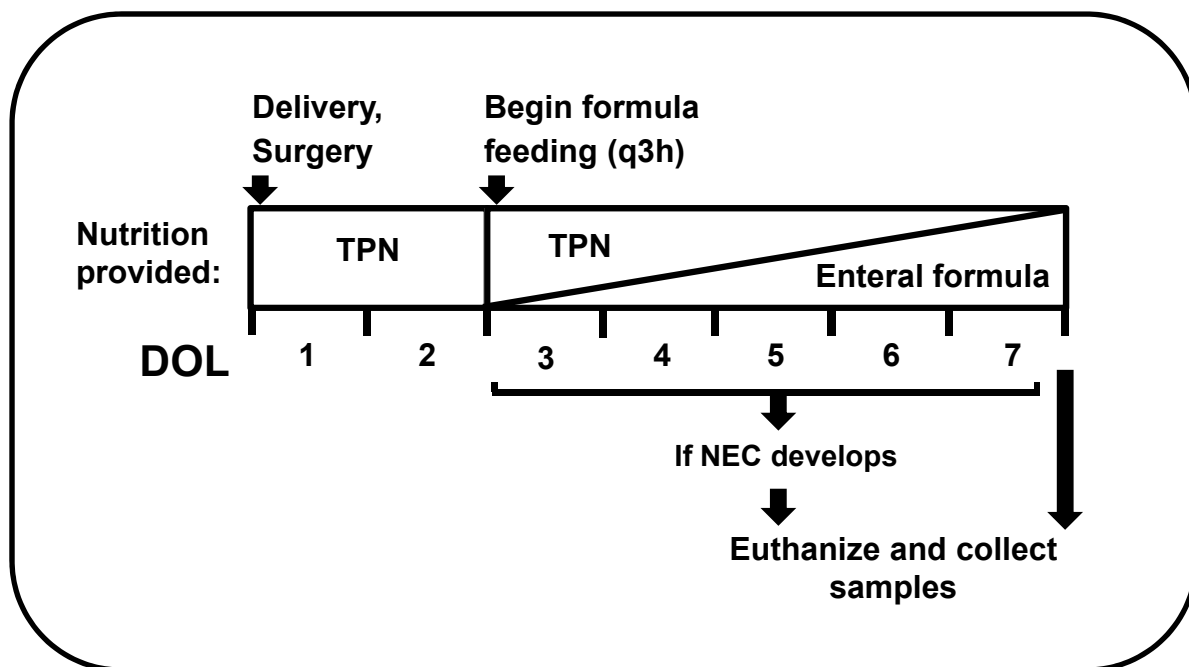

Supplement: Supplementary file 10 — Overview of the study design and experimental groups. Diagram of the timing of major components of the experiment, including introduction and weaning of total parenteral nutrition (TPN), introduction and ramp-up of enteral formula feeding, and the time of euthanasia and tissue collection for the three experimental groups. (PDF 86 kb) [file 40168_2018_498_MOESM10_ESM.pdf]
