# Supplementary material for: Metabolomic signatures distinguish the impact of formula carbohydrates on disease outcome in a preterm piglet model of NEC
Source: Microbiome. 2018 Jun 19;6:111. doi: 10.1186/s40168-018-0498-0 (PMC6009052; doi:10.1186/s40168-018-0498-0)
Supplement: Supplementary file 12 — Data, metadata files, and R scripts used to generate the figures. The Rmarkdown_data_metadata.zip is a compressed folder containing the Rmarkdown file, which details the analysis scripts (in both RMD and HTML format). The batch file used to process the sequence data in mothur is provided. Microbiome data is included here as a BIOM-formatted table. All other data (metabolomics and cytokine expression) along with metadata are CSV files. (ZIP 992 kb) [file 40168_2018_498_MOESM12_ESM.zip › cho_nec_manuscript_all_analysis.nb.html]

Metabolomic Signatures Distinguish the Impact of Formula Carbohydrates on Disease Outcome in a Preterm Piglet Model of NEC


Code 

- Show All Code
- Hide All Code
- Download Rmd

# Metabolomic Signatures Distinguish the Impact of Formula Carbohydrates on Disease Outcome in a Preterm Piglet Model of NEC

This document is a record of all analysis steps required to reproduce the work reported in the manuscript. Although some formatting differences may exist from the final published figures, the main results and conclusions are equivalent. Data files used for this analysis should be placed in the same folder as this .Rmd file.


```
knitr::opts_chunk$set(warning=FALSE, message=FALSE)
```


```
library(multcomp)
```


```
library(ggplot2)
```


```
library(reshape2)
```


```
library(plyr)
```


```
library(scales)
```


```
library(ggthemes)
```


```
library(phyloseq)
```


```
library(gridExtra)
```


```
library(RColorBrewer)
```


```
library(pheatmap)
```


```
library(ggrepel)
```


```
colors=c('#4daf4a', '#377eb8', '#e41a1c')
group_colors=c('#4daf4a', '#377eb8', '#e41a1c')
diet_colors <- c('#4daf4a', '#377eb8', '#e41a1c')
hn_colors <- c('black', 'red2') 
group_NEC_colors <- c('#4daf4a', '#316f2f', '#377eb8', '#1e4666', '#e41a1c', '#b01414')  
# set theme
theme_set(theme_classic() + theme(text = element_text(family = 'Arial', face = 'bold', color = 1),
                                  axis.text = element_text(color = 1, size = rel(1.1))))
theme_update(axis.text = element_text(color = 'black'),
             strip.text = element_text(color = 'black'),
             axis.ticks = element_line(color = 'black'))
windowsFonts(Arial=windowsFont("TT Arial"))
morph.written = FALSE  
# utility functions to be used in analysis of piglet NEC carbohydrate study
getMJNPigs <- function(incl.new = FALSE) {
  # read in data
  pigs <- read.csv('pigs_anthropometrics_morphometry_severity.csv', strip.white=T)
  
  # parse 'pigID' into separate columns for litter, letter
  pigs <- cbind(factor(unlist(lapply(pigs[, 'pigID'], function(x){substr(x, 4, 4)}))), pigs)
  pigs <- cbind(factor(unlist(lapply(pigs[, 'pigID'], function(x){as.numeric(substr(x, 1, 3))}))), pigs)
  names(pigs)[1:2] <- c('litter', 'letter')
  
  # add in calculated columns to pigs
  pigs$clin.tot <- pigs$clin.st + pigs$clin.je + pigs$clin.il + pigs$clin.co
  pigs$hist.tot <- pigs$hist.pj + pigs$hist.di + pigs$hist.co
  pigs$clin.max <- apply(pigs[, c('clin.st', 'clin.je', 'clin.il', 'clin.co')], 1, 
                         function(x){suppressWarnings(max(x, na.rm=T))})
  pigs$hist.max <- apply(pigs[, c('hist.pj', 'hist.di', 'hist.co')], 1,
                         function(x){suppressWarnings(max(x, na.rm=T))})
  pigs$clin.nec <- pigs$clin.max > 2
  pigs$clin.nec <- factor(pigs$clin.nec, labels=c('Healthy', 'NEC'))
  pigs$hist.nec <- pigs$hist.max > 1
  pigs$hist.nec <- factor(pigs$hist.nec, labels=c('Healthy', 'NEC'))
  
  pigs$dob <- as.Date(as.character(pigs$dob), '%Y%m%d')
  pigs$dod <- as.Date(as.character(pigs$dod), '%Y%m%d')
  
  # convert time of death (tod) to hours in decimal form
  pigs$tod.d <- sapply(strsplit(as.character(pigs[, 'tod']), ':'), function(x){
    x <- as.numeric(x)
    x[1]+x[2]/60
  })
  
  # 24 hrs for each day of feeding (first 2 days of life are TPN only) plus the time of death 
  #   on final day minus 12 hrs b/c feeding begins at 12:00
  pigs$hrs.after.feed <- as.numeric((pigs$dod-pigs$dob-2)*24 + pigs$tod.d - 12) 
  
  # weight.gain expressed as:  g / (kg * day)  from birthweight to deathweight
  pigs$weight.gain <- (pigs$weight.d - pigs$weight.b) / 
    ((pigs$weight.b / 1000) * (pigs$hrs.after.feed / 24))
  
  # save new file w/ added columns (only first time this function is called)
  if(!morph.written) {
    write.csv(pigs, 'pigs_anthropometrics_morphometry_severity_processed.csv', row.names = F)
    morph.written = TRUE
  }
  
  # according to preference passed in function call, include or remove all newborn
  if(incl.new) {
    # force use of this order L -> R
    pigs$group <- factor(pigs$group, levels=c('NEW', 'LAC', 'MIX', 'CSS'), ordered=T)   
  }
  else {
    pigs <- pigs[which(pigs[, 'group'] != 'NEW'), ]  
    pigs$group <- droplevels(pigs$group)
    # force use of this order L -> R
    pigs$group <- factor(pigs$group, levels=c('LAC', 'MIX', 'CSS'), ordered=T)  
  }
  
  # remove pigs from litters 228 (no amplification), 234 (early deaths), 
  #   and 248-249 (fridge died - used different formula)
  pigs <- pigs[!c(pigs$litter %in% c(228, 234, 248, 249)), ]
  
  # remove pigs found with perforated stomachs from orogastric tube
  pigs <- pigs[!c(pigs$pigID %in% c('215E','215I','215L')), ]
  
  # remove pigs which received lactose or mix diet from litters 212, 213 
  #   (bad batch of lactose/mix formulas)
  pigs <- pigs[!c(pigs$litter %in% c(212, 213) & pigs$group %in% c('LAC', 'MIX')), ]
  pigs$litter <- droplevels(pigs$litter)
  
  return(pigs)
}
se <- function(x) {
  sd(x) / sqrt(length(x))
}
## Gives count, mean, standard deviation, standard error of the mean, and 
##    confidence interval (default 95%).
##   data: a data frame.
##   measurevar: the name of a column that contains the variable to be summariezed
##   groupvars: a vector containing names of columns that contain grouping variables
##   na.rm: a boolean that indicates whether to ignore NA's
##   conf.interval: the percent range of the confidence interval (default is 95%)
#####  from http://www.cookbook-r.com/Graphs/Plotting_means_and_error_bars_(ggplot2)
summarySE <- function(data=NULL, measurevar, groupvars=NULL, na.rm=FALSE,
                      conf.interval=.95, .drop=TRUE) {
  
  # New version of length which can handle NA's: if na.rm==T, don't count them
  length2 <- function (x, na.rm=FALSE) {
    if (na.rm) sum(!is.na(x))
    else       length(x)
  }
  
  # This does the summary. For each group's data frame, return a vector with
  # N, mean, and sd
  datac <- ddply(data, groupvars, .drop=.drop,
                 .fun = function(xx, col) {
                   c(N    = length2(xx[[col]], na.rm=na.rm),
                     mean = mean   (xx[[col]], na.rm=na.rm),
                     sd   = sd     (xx[[col]], na.rm=na.rm)
                   )
                 },
                 measurevar
  )
  
  # Rename the "mean" column    
  datac <- rename(datac, c("mean" = measurevar))
  
  datac$se <- datac$sd / sqrt(datac$N)  # Calculate standard error of the mean
  
  # Confidence interval multiplier for standard error
  # Calculate t-statistic for confidence interval: 
  # e.g., if conf.interval is .95, use .975 (above/below), and use df=N-1
  ciMult <- qt(conf.interval/2 + .5, datac$N-1)
  datac$ci <- datac$se * ciMult
  
  return(datac)
}
brightness <- function(rgbcol, v) {
  conv <- as.list(as.data.frame(t(rgb2hsv(col2rgb(rgbcol)))))
  conv[[3]] <- v
  do.call(hsv, conv)
}
```


## Table 2. Body weights and gut morphometry


```
pigs <- getMJNPigs(incl.new = F)
# descriptive summaries
with(pigs, table(group, hist.nec))
```


```
     hist.nec
group Healthy NEC
  LAC      18   3
  MIX       6   6
  CSS      25  20
```


```
with(pigs, aggregate(hrs.after.feed ~ group + hist.nec, FUN=mean))
```


```
with(pigs, aggregate(hrs.after.feed ~ group + hist.nec, FUN=se))
```


```
with(pigs, aggregate(weight.b ~ group + hist.nec, FUN=mean))
```


```
with(pigs, aggregate(weight.b ~ group + hist.nec, FUN=se))
```


```
with(pigs, aggregate(weight.d ~ group + hist.nec, FUN=mean))
```


```
with(pigs, aggregate(weight.d ~ group + hist.nec, FUN=se))
```


```
with(pigs, aggregate(weight.gain ~ group + hist.nec, FUN=mean))
```


```
with(pigs, aggregate(weight.gain ~ group + hist.nec, FUN=se))
```


```
with(pigs, aggregate(pj.vh ~ group + hist.nec, FUN=mean))
```


```
with(pigs, aggregate(pj.vh ~ group + hist.nec, FUN=se))
```


```
with(pigs, aggregate(pj.cd ~ group + hist.nec, FUN=mean))
```


```
with(pigs, aggregate(pj.cd ~ group + hist.nec, FUN=se))
```


```
with(pigs, aggregate(di.vh ~ group + hist.nec, FUN=mean))
```


```
with(pigs, aggregate(di.vh ~ group + hist.nec, FUN=se))
```


```
with(pigs, aggregate(di.cd ~ group + hist.nec, FUN=mean))
```


```
with(pigs, aggregate(di.cd ~ group + hist.nec, FUN=se))
```


```
with(pigs, aggregate(co.cd ~ group + hist.nec, FUN=mean))
```


```
with(pigs, aggregate(co.cd ~ group + hist.nec, FUN=se))
```


```
# test for differences between healthy groups
pigs.h <- pigs[which(pigs[, 'hist.nec'] == 'Healthy'), ]
summary(glht(glm(hrs.after.feed ~ group, family=gaussian, data = pigs.h), linfct = mcp(group='Tukey')))
```


```
     Simultaneous Tests for General Linear Hypotheses

Multiple Comparisons of Means: Tukey Contrasts


Fit: glm(formula = hrs.after.feed ~ group, family = gaussian, data = pigs.h)

Linear Hypotheses:
               Estimate Std. Error z value Pr(>|z|)
MIX - LAC == 0   14.997     12.308   1.218    0.436
CSS - LAC == 0   -1.629      8.071  -0.202    0.977
CSS - MIX == 0  -16.627     11.870  -1.401    0.334
(Adjusted p values reported -- single-step method)
```


```
summary(glht(glm(weight.b ~ group, family=gaussian, data=pigs.h), linfct = mcp(group='Tukey')))
```


```
     Simultaneous Tests for General Linear Hypotheses

Multiple Comparisons of Means: Tukey Contrasts


Fit: glm(formula = weight.b ~ group, family = gaussian, data = pigs.h)

Linear Hypotheses:
               Estimate Std. Error z value Pr(>|z|)  
MIX - LAC == 0    76.50      96.29   0.794   0.7015  
CSS - LAC == 0   131.68      63.14   2.085   0.0897 .
CSS - MIX == 0    55.18      92.86   0.594   0.8199  
---
Signif. codes:  0 ‘***’ 0.001 ‘**’ 0.01 ‘*’ 0.05 ‘.’ 0.1 ‘ ’ 1
(Adjusted p values reported -- single-step method)
```


```
summary(glht(glm(weight.d ~ group, family=gaussian, data=pigs.h), linfct = mcp(group='Tukey')))
```


```
     Simultaneous Tests for General Linear Hypotheses

Multiple Comparisons of Means: Tukey Contrasts


Fit: glm(formula = weight.d ~ group, family = gaussian, data = pigs.h)

Linear Hypotheses:
               Estimate Std. Error z value Pr(>|z|)
MIX - LAC == 0   128.67     169.60   0.759    0.724
CSS - LAC == 0   147.52     111.21   1.326    0.374
CSS - MIX == 0    18.85     163.56   0.115    0.993
(Adjusted p values reported -- single-step method)
```


```
summary(glht(glm(weight.gain ~ group, family=gaussian, data=pigs.h), linfct = mcp(group='Tukey')))
```


```
     Simultaneous Tests for General Linear Hypotheses

Multiple Comparisons of Means: Tukey Contrasts


Fit: glm(formula = weight.gain ~ group, family = gaussian, data = pigs.h)

Linear Hypotheses:
               Estimate Std. Error z value Pr(>|z|)
MIX - LAC == 0   -5.158     12.495  -0.413    0.909
CSS - LAC == 0   -9.939      8.193  -1.213    0.439
CSS - MIX == 0   -4.782     12.049  -0.397    0.915
(Adjusted p values reported -- single-step method)
```


```
summary(glht(glm(pj.vh ~ group, family=gaussian, data=pigs.h), linfct = mcp(group='Tukey')))
```


```
     Simultaneous Tests for General Linear Hypotheses

Multiple Comparisons of Means: Tukey Contrasts


Fit: glm(formula = pj.vh ~ group, family = gaussian, data = pigs.h)

Linear Hypotheses:
               Estimate Std. Error z value Pr(>|z|)  
MIX - LAC == 0   -40.75      70.50  -0.578   0.8290  
CSS - LAC == 0   101.51      47.07   2.157   0.0764 .
CSS - MIX == 0   142.25      67.77   2.099   0.0874 .
---
Signif. codes:  0 ‘***’ 0.001 ‘**’ 0.01 ‘*’ 0.05 ‘.’ 0.1 ‘ ’ 1
(Adjusted p values reported -- single-step method)
```


```
summary(glht(glm(pj.cd ~ group, family=gaussian, data=pigs.h), linfct = mcp(group='Tukey')))
```


```
     Simultaneous Tests for General Linear Hypotheses

Multiple Comparisons of Means: Tukey Contrasts


Fit: glm(formula = pj.cd ~ group, family = gaussian, data = pigs.h)

Linear Hypotheses:
               Estimate Std. Error z value Pr(>|z|)
MIX - LAC == 0   13.245      9.627   1.376    0.348
CSS - LAC == 0    8.397      6.426   1.307    0.385
CSS - MIX == 0   -4.848      9.253  -0.524    0.857
(Adjusted p values reported -- single-step method)
```


```
summary(glht(glm(di.vh ~ group, family=gaussian, data=pigs.h), linfct = mcp(group='Tukey')))
```


```
     Simultaneous Tests for General Linear Hypotheses

Multiple Comparisons of Means: Tukey Contrasts


Fit: glm(formula = di.vh ~ group, family = gaussian, data = pigs.h)

Linear Hypotheses:
               Estimate Std. Error z value Pr(>|z|)
MIX - LAC == 0   -18.67      80.07  -0.233    0.970
CSS - LAC == 0    96.72      53.45   1.810    0.162
CSS - MIX == 0   115.39      76.96   1.499    0.286
(Adjusted p values reported -- single-step method)
```


```
summary(glht(glm(di.cd ~ group, family=gaussian, data=pigs.h), linfct = mcp(group='Tukey')))
```


```
     Simultaneous Tests for General Linear Hypotheses

Multiple Comparisons of Means: Tukey Contrasts


Fit: glm(formula = di.cd ~ group, family = gaussian, data = pigs.h)

Linear Hypotheses:
               Estimate Std. Error z value Pr(>|z|)
MIX - LAC == 0   17.578     13.095   1.342    0.366
CSS - LAC == 0    7.636      8.742   0.874    0.652
CSS - MIX == 0   -9.942     12.587  -0.790    0.705
(Adjusted p values reported -- single-step method)
```


```
summary(glht(glm(co.cd ~ group, family=gaussian, data=pigs.h), linfct = mcp(group='Tukey')))
```


```
     Simultaneous Tests for General Linear Hypotheses

Multiple Comparisons of Means: Tukey Contrasts


Fit: glm(formula = co.cd ~ group, family = gaussian, data = pigs.h)

Linear Hypotheses:
               Estimate Std. Error z value Pr(>|z|)  
MIX - LAC == 0   -48.54      22.94  -2.116    0.084 .
CSS - LAC == 0   -15.99      15.46  -1.034    0.550  
CSS - MIX == 0    32.55      21.87   1.488    0.291  
---
Signif. codes:  0 ‘***’ 0.001 ‘**’ 0.01 ‘*’ 0.05 ‘.’ 0.1 ‘ ’ 1
(Adjusted p values reported -- single-step method)
```


```
# test for differences between NEC groups
pigs.n <- pigs[which(pigs[, 'hist.nec'] == 'NEC'), ]
summary(glht(glm(hrs.after.feed ~ group, family=gaussian, data = pigs.n), linfct = mcp(group='Tukey')))
```


```
     Simultaneous Tests for General Linear Hypotheses

Multiple Comparisons of Means: Tukey Contrasts


Fit: glm(formula = hrs.after.feed ~ group, family = gaussian, data = pigs.n)

Linear Hypotheses:
               Estimate Std. Error z value Pr(>|z|)
MIX - LAC == 0   -7.653     27.975  -0.274    0.959
CSS - LAC == 0  -21.429     24.494  -0.875    0.649
CSS - MIX == 0  -13.776     18.415  -0.748    0.729
(Adjusted p values reported -- single-step method)
```


```
summary(glht(glm(weight.b ~ group, family=gaussian, data=pigs.n), linfct = mcp(group='Tukey')))
```


```
     Simultaneous Tests for General Linear Hypotheses

Multiple Comparisons of Means: Tukey Contrasts


Fit: glm(formula = weight.b ~ group, family = gaussian, data = pigs.n)

Linear Hypotheses:
               Estimate Std. Error z value Pr(>|z|)
MIX - LAC == 0   137.83     157.27   0.876    0.648
CSS - LAC == 0    53.22     137.70   0.386    0.919
CSS - MIX == 0   -84.62     103.53  -0.817    0.686
(Adjusted p values reported -- single-step method)
```


```
summary(glht(glm(weight.d ~ group, family=gaussian, data=pigs.n), linfct = mcp(group='Tukey')))
```


```
     Simultaneous Tests for General Linear Hypotheses

Multiple Comparisons of Means: Tukey Contrasts


Fit: glm(formula = weight.d ~ group, family = gaussian, data = pigs.n)

Linear Hypotheses:
                Estimate Std. Error z value Pr(>|z|)
MIX - LAC == 0  107.0000   281.6659   0.380    0.922
CSS - LAC == 0    0.4833   246.6253   0.002    1.000
CSS - MIX == 0 -106.5167   185.4152  -0.574    0.830
(Adjusted p values reported -- single-step method)
```


```
summary(glht(glm(weight.gain ~ group, family=gaussian, data=pigs.n), linfct = mcp(group='Tukey')))
```


```
     Simultaneous Tests for General Linear Hypotheses

Multiple Comparisons of Means: Tukey Contrasts


Fit: glm(formula = weight.gain ~ group, family = gaussian, data = pigs.n)

Linear Hypotheses:
               Estimate Std. Error z value Pr(>|z|)
MIX - LAC == 0   -19.84      58.48  -0.339    0.937
CSS - LAC == 0   -45.14      51.20  -0.882    0.645
CSS - MIX == 0   -25.30      38.49  -0.657    0.783
(Adjusted p values reported -- single-step method)
```


```
summary(glht(glm(pj.vh ~ group, family=gaussian, data=pigs.n), linfct = mcp(group='Tukey')))
```


```
     Simultaneous Tests for General Linear Hypotheses

Multiple Comparisons of Means: Tukey Contrasts


Fit: glm(formula = pj.vh ~ group, family = gaussian, data = pigs.n)

Linear Hypotheses:
               Estimate Std. Error z value Pr(>|z|)
MIX - LAC == 0    -9.00     106.47  -0.085    0.996
CSS - LAC == 0    16.41      93.22   0.176    0.983
CSS - MIX == 0    25.41      70.08   0.363    0.928
(Adjusted p values reported -- single-step method)
```


```
summary(glht(glm(pj.cd ~ group, family=gaussian, data=pigs.n), linfct = mcp(group='Tukey')))
```


```
     Simultaneous Tests for General Linear Hypotheses

Multiple Comparisons of Means: Tukey Contrasts


Fit: glm(formula = pj.cd ~ group, family = gaussian, data = pigs.n)

Linear Hypotheses:
               Estimate Std. Error z value Pr(>|z|)
MIX - LAC == 0    4.167     13.581   0.307    0.948
CSS - LAC == 0    8.262     11.892   0.695    0.761
CSS - MIX == 0    4.096      8.940   0.458    0.888
(Adjusted p values reported -- single-step method)
```


```
summary(glht(glm(di.vh ~ group, family=gaussian, data=pigs.n), linfct = mcp(group='Tukey')))
```


```
     Simultaneous Tests for General Linear Hypotheses

Multiple Comparisons of Means: Tukey Contrasts


Fit: glm(formula = di.vh ~ group, family = gaussian, data = pigs.n)

Linear Hypotheses:
               Estimate Std. Error z value Pr(>|z|)
MIX - LAC == 0    98.00     122.71   0.799    0.698
CSS - LAC == 0   112.35     108.68   1.034    0.549
CSS - MIX == 0    14.35      82.41   0.174    0.983
(Adjusted p values reported -- single-step method)
```


```
summary(glht(glm(di.cd ~ group, family=gaussian, data=pigs.n), linfct = mcp(group='Tukey')))
```


```
     Simultaneous Tests for General Linear Hypotheses

Multiple Comparisons of Means: Tukey Contrasts


Fit: glm(formula = di.cd ~ group, family = gaussian, data = pigs.n)

Linear Hypotheses:
               Estimate Std. Error z value Pr(>|z|)
MIX - LAC == 0  -22.500     18.211  -1.236    0.424
CSS - LAC == 0  -15.057     15.945  -0.944    0.605
CSS - MIX == 0    7.442     11.988   0.621    0.804
(Adjusted p values reported -- single-step method)
```


```
summary(glht(glm(co.cd ~ group, family=gaussian, data=pigs.n), linfct = mcp(group='Tukey')))
```


```
     Simultaneous Tests for General Linear Hypotheses

Multiple Comparisons of Means: Tukey Contrasts


Fit: glm(formula = co.cd ~ group, family = gaussian, data = pigs.n)

Linear Hypotheses:
               Estimate Std. Error z value Pr(>|z|)    
MIX - LAC == 0  -165.67      42.35  -3.912 0.000251 ***
CSS - LAC == 0  -101.29      38.56  -2.627 0.021986 *  
CSS - MIX == 0    64.38      24.29   2.651 0.020530 *  
---
Signif. codes:  0 ‘***’ 0.001 ‘**’ 0.01 ‘*’ 0.05 ‘.’ 0.1 ‘ ’ 1
(Adjusted p values reported -- single-step method)
```


```
# test for differences between healthy vs NEC within groups
with(pigs[which(pigs$group == 'LAC'), ], t.test(hrs.after.feed ~ hist.nec))
```


```
    Welch Two Sample t-test

data:  hrs.after.feed by hist.nec
t = 0.96692, df = 2.3512, p-value = 0.4222
alternative hypothesis: true difference in means is not equal to 0
95 percent confidence interval:
 -65.08199 110.43755
sample estimates:
mean in group Healthy     mean in group NEC 
            105.81667              83.13889
```


```
with(pigs[which(pigs$group == 'LAC'), ], t.test(weight.b ~ hist.nec))
```


```
    Welch Two Sample t-test

data:  weight.b by hist.nec
t = 1.1643, df = 14.072, p-value = 0.2637
alternative hypothesis: true difference in means is not equal to 0
95 percent confidence interval:
 -51.87773 175.21107
sample estimates:
mean in group Healthy     mean in group NEC 
             934.0000              872.3333
```


```
with(pigs[which(pigs$group == 'LAC'), ], t.test(weight.d ~ hist.nec))
```


```
    Welch Two Sample t-test

data:  weight.d by hist.nec
t = 1.2699, df = 4.3218, p-value = 0.2682
alternative hypothesis: true difference in means is not equal to 0
95 percent confidence interval:
 -201.4950  560.1617
sample estimates:
mean in group Healthy     mean in group NEC 
             1382.000              1202.667
```


```
with(pigs[which(pigs$group == 'LAC'), ], t.test(weight.gain ~ hist.nec))
```


```
    Welch Two Sample t-test

data:  weight.gain by hist.nec
t = -0.10222, df = 2.5644, p-value = 0.926
alternative hypothesis: true difference in means is not equal to 0
95 percent confidence interval:
 -67.15958  63.35972
sample estimates:
mean in group Healthy     mean in group NEC 
             104.2771              106.1771
```


```
with(pigs[which(pigs$group == 'LAC'), ], t.test(pj.vh ~ hist.nec))
```


```
    Welch Two Sample t-test

data:  pj.vh by hist.nec
t = 3.5965, df = 11.495, p-value = 0.003919
alternative hypothesis: true difference in means is not equal to 0
95 percent confidence interval:
  60.01719 246.80634
sample estimates:
mean in group Healthy     mean in group NEC 
             467.4118              314.0000
```


```
with(pigs[which(pigs$group == 'LAC'), ], t.test(pj.cd ~ hist.nec))
```


```
    Welch Two Sample t-test

data:  pj.cd by hist.nec
t = 2.1432, df = 2.7247, p-value = 0.1306
alternative hypothesis: true difference in means is not equal to 0
95 percent confidence interval:
 -10.66067  47.83714
sample estimates:
mean in group Healthy     mean in group NEC 
             93.58824              75.00000
```


```
with(pigs[which(pigs$group == 'LAC'), ], t.test(di.vh ~ hist.nec))
```


```
    Welch Two Sample t-test

data:  di.vh by hist.nec
t = 6.4123, df = 2.6763, p-value = 0.01076
alternative hypothesis: true difference in means is not equal to 0
95 percent confidence interval:
 167.5261 548.4739
sample estimates:
mean in group Healthy     mean in group NEC 
                  537                   179
```


```
with(pigs[which(pigs$group == 'LAC'), ], t.test(di.cd ~ hist.nec))
```


```
    Welch Two Sample t-test

data:  di.cd by hist.nec
t = 2.321, df = 17.26, p-value = 0.03277
alternative hypothesis: true difference in means is not equal to 0
95 percent confidence interval:
  1.526408 31.650063
sample estimates:
mean in group Healthy     mean in group NEC 
             113.5882               97.0000
```


```
with(pigs[which(pigs$group == 'LAC'), ], t.test(co.cd ~ hist.nec))
```


```
    Welch Two Sample t-test

data:  co.cd by hist.nec
t = -5.8213, df = 15.524, p-value = 2.935e-05
alternative hypothesis: true difference in means is not equal to 0
95 percent confidence interval:
 -112.1065  -52.1435
sample estimates:
mean in group Healthy     mean in group NEC 
              231.875               314.000
```


```
with(pigs[which(pigs$group == 'MIX'), ], t.test(hrs.after.feed ~ hist.nec))
```


```
    Welch Two Sample t-test

data:  hrs.after.feed by hist.nec
t = 3.6226, df = 5.0339, p-value = 0.015
alternative hypothesis: true difference in means is not equal to 0
95 percent confidence interval:
 13.22872 77.42684
sample estimates:
mean in group Healthy     mean in group NEC 
            120.81389              75.48611
```


```
with(pigs[which(pigs$group == 'MIX'), ], t.test(weight.b ~ hist.nec))
```


```
    Welch Two Sample t-test

data:  weight.b by hist.nec
t = 0.00268, df = 7.9961, p-value = 0.9979
alternative hypothesis: true difference in means is not equal to 0
95 percent confidence interval:
 -286.5130  287.1796
sample estimates:
mean in group Healthy     mean in group NEC 
             1010.500              1010.167
```


```
with(pigs[which(pigs$group == 'MIX'), ], t.test(weight.d ~ hist.nec))
```


```
    Welch Two Sample t-test

data:  weight.d by hist.nec
t = 1.3486, df = 8.9841, p-value = 0.2105
alternative hypothesis: true difference in means is not equal to 0
95 percent confidence interval:
 -136.2621  538.2621
sample estimates:
mean in group Healthy     mean in group NEC 
             1510.667              1309.667
```


```
with(pigs[which(pigs$group == 'MIX'), ], t.test(weight.gain ~ hist.nec))
```


```
    Welch Two Sample t-test

data:  weight.gain by hist.nec
t = 0.42382, df = 5.3927, p-value = 0.6881
alternative hypothesis: true difference in means is not equal to 0
95 percent confidence interval:
 -63.10247  88.67687
sample estimates:
mean in group Healthy     mean in group NEC 
              99.1196               86.3324
```


```
with(pigs[which(pigs$group == 'MIX'), ], t.test(pj.vh ~ hist.nec))
```


```
    Welch Two Sample t-test

data:  pj.vh by hist.nec
t = 2.4122, df = 9.6673, p-value = 0.03733
alternative hypothesis: true difference in means is not equal to 0
95 percent confidence interval:
   8.759161 234.574172
sample estimates:
mean in group Healthy     mean in group NEC 
             426.6667              305.0000
```


```
with(pigs[which(pigs$group == 'MIX'), ], t.test(pj.cd ~ hist.nec))
```


```
    Welch Two Sample t-test

data:  pj.cd by hist.nec
t = 2.3434, df = 9.9198, p-value = 0.0413
alternative hypothesis: true difference in means is not equal to 0
95 percent confidence interval:
  1.331718 54.001616
sample estimates:
mean in group Healthy     mean in group NEC 
            106.83333              79.16667
```


```
with(pigs[which(pigs$group == 'MIX'), ], t.test(di.vh ~ hist.nec))
```


```
    Welch Two Sample t-test

data:  di.vh by hist.nec
t = 4.2707, df = 5.4946, p-value = 0.00642
alternative hypothesis: true difference in means is not equal to 0
95 percent confidence interval:
  99.91738 382.74929
sample estimates:
mean in group Healthy     mean in group NEC 
             518.3333              277.0000
```


```
with(pigs[which(pigs$group == 'MIX'), ], t.test(di.cd ~ hist.nec))
```


```
    Welch Two Sample t-test

data:  di.cd by hist.nec
t = 3.7117, df = 9.0288, p-value = 0.004805
alternative hypothesis: true difference in means is not equal to 0
95 percent confidence interval:
 22.14735 91.18599
sample estimates:
mean in group Healthy     mean in group NEC 
             131.1667               74.5000
```


```
with(pigs[which(pigs$group == 'MIX'), ], t.test(co.cd ~ hist.nec))
```


```
    Welch Two Sample t-test

data:  co.cd by hist.nec
t = 2.1024, df = 6.1779, p-value = 0.07888
alternative hypothesis: true difference in means is not equal to 0
95 percent confidence interval:
 -5.453535 75.453535
sample estimates:
mean in group Healthy     mean in group NEC 
             183.3333              148.3333
```


```
with(pigs[which(pigs$group == 'CSS'), ], t.test(hrs.after.feed ~ hist.nec))
```


```
    Welch Two Sample t-test

data:  hrs.after.feed by hist.nec
t = 3.9254, df = 31.554, p-value = 0.0004394
alternative hypothesis: true difference in means is not equal to 0
95 percent confidence interval:
 20.42309 64.53158
sample estimates:
mean in group Healthy     mean in group NEC 
             104.1873               61.7100
```


```
with(pigs[which(pigs$group == 'CSS'), ], t.test(weight.b ~ hist.nec))
```


```
    Welch Two Sample t-test

data:  weight.b by hist.nec
t = 2.1028, df = 40.951, p-value = 0.04167
alternative hypothesis: true difference in means is not equal to 0
95 percent confidence interval:
   5.544424 274.715576
sample estimates:
mean in group Healthy     mean in group NEC 
              1065.68                925.55
```


```
with(pigs[which(pigs$group == 'CSS'), ], t.test(weight.d ~ hist.nec))
```


```
    Welch Two Sample t-test

data:  weight.d by hist.nec
t = 2.6063, df = 38.851, p-value = 0.01291
alternative hypothesis: true difference in means is not equal to 0
95 percent confidence interval:
  73.05244 579.68756
sample estimates:
mean in group Healthy     mean in group NEC 
              1529.52               1203.15
```


```
with(pigs[which(pigs$group == 'CSS'), ], t.test(weight.gain ~ hist.nec))
```


```
    Welch Two Sample t-test

data:  weight.gain by hist.nec
t = 1.6147, df = 22.037, p-value = 0.1206
alternative hypothesis: true difference in means is not equal to 0
95 percent confidence interval:
 -9.466894 76.069733
sample estimates:
mean in group Healthy     mean in group NEC 
             94.33767              61.03625
```


```
with(pigs[which(pigs$group == 'CSS'), ], t.test(pj.vh ~ hist.nec))
```


```
    Welch Two Sample t-test

data:  pj.vh by hist.nec
t = 4.7236, df = 39.692, p-value = 2.886e-05
alternative hypothesis: true difference in means is not equal to 0
95 percent confidence interval:
 136.4352 340.5837
sample estimates:
mean in group Healthy     mean in group NEC 
             568.9188              330.4093
```


```
with(pigs[which(pigs$group == 'CSS'), ], t.test(pj.cd ~ hist.nec))
```


```
    Welch Two Sample t-test

data:  pj.cd by hist.nec
t = 2.8831, df = 41.931, p-value = 0.006188
alternative hypothesis: true difference in means is not equal to 0
95 percent confidence interval:
  5.616764 31.829069
sample estimates:
mean in group Healthy     mean in group NEC 
             101.9854               83.2625
```


```
with(pigs[which(pigs$group == 'CSS'), ], t.test(di.vh ~ hist.nec))
```


```
    Welch Two Sample t-test

data:  di.vh by hist.nec
t = 5.2833, df = 37.388, p-value = 5.702e-06
alternative hypothesis: true difference in means is not equal to 0
95 percent confidence interval:
 211.1124 473.6254
sample estimates:
mean in group Healthy     mean in group NEC 
             633.7218              291.3529
```


```
with(pigs[which(pigs$group == 'CSS'), ], t.test(di.cd ~ hist.nec))
```


```
    Welch Two Sample t-test

data:  di.cd by hist.nec
t = 4.6774, df = 40.327, p-value = 3.241e-05
alternative hypothesis: true difference in means is not equal to 0
95 percent confidence interval:
 22.31294 56.25114
sample estimates:
mean in group Healthy     mean in group NEC 
             121.2245               81.9425
```


```
with(pigs[which(pigs$group == 'CSS'), ], t.test(co.cd ~ hist.nec))
```


```
    Welch Two Sample t-test

data:  co.cd by hist.nec
t = 0.1941, df = 32.23, p-value = 0.8473
alternative hypothesis: true difference in means is not equal to 0
95 percent confidence interval:
 -30.08941  36.42976
sample estimates:
mean in group Healthy     mean in group NEC 
             215.8833              212.7132
```


```
rm(pigs.h, pigs.n, pigs)
```

## Figure 1. Phenotypic outcomes


```
pigs <- getMJNPigs(incl.new = F)
# incidence plot
t<-with(pigs,table(group, hist.nec))
pigs$hist.nec.logical <- pigs$hist.nec == 'NEC'
clin_nec_incidence_summary <- summarySE(pigs, measurevar = 'hist.nec.logical', groupvars = 'group')
ggplot(clin_nec_incidence_summary, aes(x = group, y = hist.nec.logical, fill = group)) + 
  scale_fill_manual(values = colors) + geom_bar(stat = 'identity', width = 0.8) +
  annotate('text', x = 1:3, y = 0.05, label = paste(t[,2], clin_nec_incidence_summary$N, sep = '/'),
           fontface = 'bold', color = 'white', cex = 6) +
  labs(list(title = 'NEC Incidence', x = '', y = '')) + scale_y_continuous(element_blank()) +
  theme(panel.grid.major = element_blank(), panel.grid.minor = element_blank(),
        axis.line = element_line(colour = 'black'), axis.title.x = element_text(vjust = -0.5),
        axis.title.y = element_text(vjust = 1.5), text = element_text(face = 'bold', size = 18),
        axis.text = element_text(color = 'black'), legend.title = element_blank(),
        legend.position = 'none')
rm(t, clin_nec_incidence_summary)
# incidence stats
addmargins(table(pigs$group, pigs$hist.nec, useNA='ifany'))
```


```
      Healthy NEC Sum
  LAC      18   3  21
  MIX       6   6  12
  CSS      25  20  45
  Sum      49  29  78
```


```
fisher.test(pigs$group, pigs$hist.nec)
```


```
    Fisher's Exact Test for Count Data

data:  pigs$group and pigs$hist.nec
p-value = 0.03052
alternative hypothesis: two.sided
```


```
# survival curve plot
# count any NEC pig sacrificed before 9am (beginning of collection day)
#   (aka 117 = 3 hrs less than 120) as an event
surv_model <- with(pigs, Surv(hrs.after.feed, as.numeric(hrs.after.feed < 119 & hist.nec == 'NEC')))
sf  <-  survfit(surv_model ~ group, conf.type="log", 
                conf.int=0.95, type="kaplan-meier", error="greenwood", data=pigs)
op <- par(font=2, cex.axis=1.5, cex.lab=1.8, mar = c(5, 7, 4, 2) + 0.1)
```


```
plot(sf, lty=1, lwd=4, font=2, font.lab=2, mark.time=TRUE, xlab='Hours of enteral feeding',
     frame.plot=F, xaxt='n', yaxt='n', col=colors)
rect(0, 0, 124, 1, col = 'white', lty=0)
```


```
axis(1, lwd=2, font=2, font.lab=2, at=c(0, 140), lwd.ticks=0, pos=c(0, 0))
axis(1, at=seq(0, 130, by=24), lwd=0, lwd.ticks=1, font=2, pos=c(0, 0))
```


```
axis(2, lwd=2, las=2, font=2, font.lab=2, at=seq(0, 1, 0.2), labels=paste(seq(0, 100, 20), "%", sep=""))
lines(sf, lty=1, lwd=4, mark.time=F, col=colors)
```


```
title(ylab='Non-NEC Survival', font.lab=2, cex.lab=1.8, line=4.5)
legend(text.width=14, x=22, y=0.35, legend=c('LAC', 'MIX', 'CSS'), col=colors, lty=1, lwd=4,
       bty="y", bg='white')
```


```
par(op)
```


```
# survival curve stats (Log-rank test)
survdiff(surv_model~group, data=pigs, rho=0)  ## overall
```


```
Call:
survdiff(formula = surv_model ~ group, data = pigs, rho = 0)

           N Observed Expected (O-E)^2/E (O-E)^2/V
group=LAC 21        3     8.52     3.579     5.164
group=MIX 12        6     4.72     0.344     0.416
group=CSS 45       19    14.75     1.223     2.597

 Chisq= 5.2  on 2 degrees of freedom, p= 0.0756
```


```
survdiff(surv_model~group, data=pigs, subset=group %in% c('LAC', 'CSS'))  ## pairwise
```


```
Call:
survdiff(formula = surv_model ~ group, data = pigs, subset = group %in% 
    c("LAC", "CSS"))

           N Observed Expected (O-E)^2/E (O-E)^2/V
group=LAC 21        3     7.98      3.11       4.9
group=CSS 45       19    14.02      1.77       4.9

 Chisq= 4.9  on 1 degrees of freedom, p= 0.0268
```


```
survdiff(surv_model~group, data=pigs, subset=group%in%c('LAC', 'MIX'))  ## pairwise
```


```
Call:
survdiff(formula = surv_model ~ group, data = pigs, subset = group %in% 
    c("LAC", "MIX"))

           N Observed Expected (O-E)^2/E (O-E)^2/V
group=LAC 21        3     5.81      1.36      3.83
group=MIX 12        6     3.19      2.47      3.83

 Chisq= 3.8  on 1 degrees of freedom, p= 0.0502
```


```
survdiff(surv_model~group, data=pigs, subset=group%in%c('CSS', 'MIX'))  ## pairwise
```


```
Call:
survdiff(formula = surv_model ~ group, data = pigs, subset = group %in% 
    c("CSS", "MIX"))

           N Observed Expected (O-E)^2/E (O-E)^2/V
group=MIX 12        6     6.05  0.000442  0.000587
group=CSS 45       19    18.95  0.000141  0.000587

 Chisq= 0  on 1 degrees of freedom, p= 0.981
```


```
rm(surv_model, sf, op)
# severity plots
idvars <- c('pigID', 'litter', 'farm', 'group', 'sex', 'hist.nec')
givars <- c('clin.st','clin.je','clin.il','clin.co')
mpigs <- melt(pigs[, c(idvars, givars)], id.vars=idvars, variable.name='site')
tmp <- summarySE(mpigs, measurevar = 'value', groupvars = c('group', 'site'), na.rm=TRUE)
with(tmp, ggplot(data=tmp, aes(x=site, fill=group, y=value)) + 
       geom_errorbar(aes(ymin = value - se, ymax = value + se), width = 0.2, size = 1.5,
                     position = position_dodge(0.9)) + 
       geom_bar(stat='identity', position=position_dodge()) + 
       scale_fill_manual(values=colors) + 
       labs(y='mean severity score'))
```


```
givars <- c('hist.pj', 'hist.di', 'hist.co')
mpigs <- melt(pigs[, c(idvars, givars)], id.vars=idvars, variable.name='site')
tmp <- summarySE(mpigs, measurevar = 'value', groupvars = c('group', 'site'), na.rm=TRUE)
with(tmp, ggplot(data=tmp, aes(x=site, fill=group, y=value)) + 
       geom_errorbar(aes(ymin = value - se, ymax = value + se), width = 0.2, size = 1.5,
                     position = position_dodge(0.9)) + 
       geom_bar(stat='identity', position=position_dodge()) + 
       scale_fill_manual(values=colors) + 
       labs(y='mean severity score'))
```


```
rm(idvars, givars, mpigs, tmp)
# severity stats
sevreg <- glm(clin.st ~ group + weight.b + farm, family=gaussian, data=pigs)
summary(glht(sevreg, linfct = mcp(group='Tukey')))
```


```
     Simultaneous Tests for General Linear Hypotheses

Multiple Comparisons of Means: Tukey Contrasts


Fit: glm(formula = clin.st ~ group + weight.b + farm, family = gaussian, 
    data = pigs)

Linear Hypotheses:
               Estimate Std. Error z value Pr(>|z|)
MIX - LAC == 0  0.57527    0.49689   1.158    0.473
CSS - LAC == 0  0.52611    0.35778   1.470    0.300
CSS - MIX == 0 -0.04916    0.44260  -0.111    0.993
(Adjusted p values reported -- single-step method)
```


```
sevreg <- glm(clin.je ~ group + weight.b + farm, family=gaussian, data=pigs)
summary(glht(sevreg, linfct = mcp(group='Tukey')))
```


```
     Simultaneous Tests for General Linear Hypotheses

Multiple Comparisons of Means: Tukey Contrasts


Fit: glm(formula = clin.je ~ group + weight.b + farm, family = gaussian, 
    data = pigs)

Linear Hypotheses:
               Estimate Std. Error z value Pr(>|z|)  
MIX - LAC == 0   0.8016     0.4892   1.639   0.2253  
CSS - LAC == 0   0.9623     0.3523   2.732   0.0168 *
CSS - MIX == 0   0.1607     0.4358   0.369   0.9266  
---
Signif. codes:  0 ‘***’ 0.001 ‘**’ 0.01 ‘*’ 0.05 ‘.’ 0.1 ‘ ’ 1
(Adjusted p values reported -- single-step method)
```


```
sevreg <- glm(clin.il ~ group + weight.b + farm, family=gaussian, data=pigs)
summary(glht(sevreg, linfct = mcp(group='Tukey')))
```


```
     Simultaneous Tests for General Linear Hypotheses

Multiple Comparisons of Means: Tukey Contrasts


Fit: glm(formula = clin.il ~ group + weight.b + farm, family = gaussian, 
    data = pigs)

Linear Hypotheses:
               Estimate Std. Error z value Pr(>|z|)  
MIX - LAC == 0   1.4771     0.5983   2.469   0.0354 *
CSS - LAC == 0   1.0860     0.4308   2.521   0.0304 *
CSS - MIX == 0  -0.3911     0.5329  -0.734   0.7398  
---
Signif. codes:  0 ‘***’ 0.001 ‘**’ 0.01 ‘*’ 0.05 ‘.’ 0.1 ‘ ’ 1
(Adjusted p values reported -- single-step method)
```


```
sevreg <- glm(clin.co ~ group + weight.b + farm, family=gaussian, data=pigs)
summary(glht(sevreg, linfct = mcp(group='Tukey')))
```


```
     Simultaneous Tests for General Linear Hypotheses

Multiple Comparisons of Means: Tukey Contrasts


Fit: glm(formula = clin.co ~ group + weight.b + farm, family = gaussian, 
    data = pigs)

Linear Hypotheses:
               Estimate Std. Error z value Pr(>|z|)  
MIX - LAC == 0   0.9039     0.5999   1.507   0.2831  
CSS - LAC == 0   1.1212     0.4320   2.596   0.0248 *
CSS - MIX == 0   0.2173     0.5344   0.407   0.9114  
---
Signif. codes:  0 ‘***’ 0.001 ‘**’ 0.01 ‘*’ 0.05 ‘.’ 0.1 ‘ ’ 1
(Adjusted p values reported -- single-step method)
```


```
sevreg <- glm(hist.pj ~ group + weight.b + farm, family=gaussian, data=pigs)
summary(glht(sevreg, linfct = mcp(group='Tukey')))
```


```
     Simultaneous Tests for General Linear Hypotheses

Multiple Comparisons of Means: Tukey Contrasts


Fit: glm(formula = hist.pj ~ group + weight.b + farm, family = gaussian, 
    data = pigs)

Linear Hypotheses:
               Estimate Std. Error z value Pr(>|z|)
MIX - LAC == 0  0.41239    0.39680   1.039    0.547
CSS - LAC == 0  0.50651    0.28614   1.770    0.176
CSS - MIX == 0  0.09413    0.35444   0.266    0.961
(Adjusted p values reported -- single-step method)
```


```
sevreg <- glm(hist.di ~ group + weight.b + farm, family=gaussian, data=pigs)
summary(glht(sevreg, linfct = mcp(group='Tukey')))
```


```
     Simultaneous Tests for General Linear Hypotheses

Multiple Comparisons of Means: Tukey Contrasts


Fit: glm(formula = hist.di ~ group + weight.b + farm, family = gaussian, 
    data = pigs)

Linear Hypotheses:
               Estimate Std. Error z value Pr(>|z|)  
MIX - LAC == 0  0.76309    0.48042   1.588   0.2464  
CSS - LAC == 0  0.73417    0.34644   2.119   0.0839 .
CSS - MIX == 0 -0.02892    0.42914  -0.067   0.9975  
---
Signif. codes:  0 ‘***’ 0.001 ‘**’ 0.01 ‘*’ 0.05 ‘.’ 0.1 ‘ ’ 1
(Adjusted p values reported -- single-step method)
```


```
sevreg <- glm(hist.co ~ group + weight.b + farm, family=gaussian, data=pigs)
summary(glht(sevreg, linfct = mcp(group='Tukey')))
```


```
     Simultaneous Tests for General Linear Hypotheses

Multiple Comparisons of Means: Tukey Contrasts


Fit: glm(formula = hist.co ~ group + weight.b + farm, family = gaussian, 
    data = pigs)

Linear Hypotheses:
               Estimate Std. Error z value Pr(>|z|)  
MIX - LAC == 0   0.4799     0.4208   1.140   0.4842  
CSS - LAC == 0   0.8374     0.3082   2.717   0.0176 *
CSS - MIX == 0   0.3576     0.3690   0.969   0.5918  
---
Signif. codes:  0 ‘***’ 0.001 ‘**’ 0.01 ‘*’ 0.05 ‘.’ 0.1 ‘ ’ 1
(Adjusted p values reported -- single-step method)
```


```
rm(sevreg, pigs)
```

## Figure 2. Microbiota richness and diversity


```
# define file locations
biom_file <- 'mothur_final.biom' 
mapping_file <- 'mappingfile_phyloseq.csv'
# create phyloseq object from input files
ps <- import_biom(biom_file)
sample_names(ps) <- make.names(sample_names(ps))
sdata <- sample_data(merge(read.csv(mapping_file),
                           getMJNPigs(incl.new = F)[, c('pigID', 'group', 'hist.nec')],
                           by = 'pigID'))
row.names(sdata) <- make.names(sdata$sampleID)
ps <- merge_phyloseq(ps, sdata)
colnames(tax_table(ps)) <- c('Domain', 'Phylum', 'Class', 'Order', 'Family', 'Genus')
sample_data(ps)$group <- factor(sample_data(ps)$group, levels = c('LAC', 'MIX', 'CSS'))
sample_data(ps)$site <- factor(sample_data(ps)$site, levels = c('Stomach', 'Ileum', 'Colon'))
# clean up taxa names
tax_table(ps) <- gsub('_', ' ', gsub('__', '', tax_table(ps)))
# remove any spurious taxa
ps <- subset_taxa(ps, Domain == 'Bacteria')
# Make a data frame with a column for the read counts of each sample
sample_sum_df <- data.frame(sum = sample_sums(ps)) 
# Histogram of sample read counts
#   by sample type (tissue v contents)
ggplot(merge(sample_sum_df, sample_data(ps), by = 'row.names'), aes(x = sum)) +  facet_grid( ~ type) +
  geom_histogram(binwidth = 500) +
  ggtitle('Distribution of sample sequencing depth (red line = 2000)') + 
  xlab('Read counts (bin=500)') +
  xlim(-500, NA) +  # -500 to ensure all data are displayed on chart; NA to set limit to data max
  geom_vline(xintercept = 2000, linetype = 'dashed', color = 'red') +
  theme(axis.title.y = element_blank())
```


```
#   by all categories (site, type, group, hist.nec)
ggplot(merge(sample_sum_df, sample_data(ps), by = 'row.names'), aes(x = sum)) +
  facet_grid(type + group ~ site + hist.nec) +
  geom_histogram(binwidth = 500) + theme_bw() +
  ggtitle('Distribution of sample sequencing depth (red line = 2000)') + 
  xlab('Read counts (bin=500)') +
  xlim(-500, NA) +  # -500 to ensure all data are displayed on chart; NA to set limit to data max
  geom_vline(xintercept = 2000, linetype = 'dashed', color = 'red') +
  theme(axis.title.y = element_blank())
```


```
# remove samples with low read counts
ps <- prune_samples(sample_sums(ps) >= 2000, ps)  # there aren't any in this data
# subset into tissue and contents
ps.t <- subset_samples(ps, type == 'Tissue')
ps.c <- subset_samples(ps, type == 'Contents')
# subset into Healthy and NEC (hist.nec)
ps.t.h <- subset_samples(ps.t, hist.nec == 'Healthy')
ps.t.n <- subset_samples(ps.t, hist.nec == 'NEC')
ps.c.h <- subset_samples(ps.c, hist.nec == 'Healthy')
ps.c.n <- subset_samples(ps.c, hist.nec == 'NEC')
# remove OTUs which are present in 0 samples
ps <- prune_taxa(taxa_sums(ps) > 0, ps)
ps.t <- prune_taxa(taxa_sums(ps.t) > 0, ps.t)
ps.t.h <- prune_taxa(taxa_sums(ps.t.h) > 0, ps.t.h)
ps.t.n <- prune_taxa(taxa_sums(ps.t.n) > 0, ps.t.n)
ps.c <- prune_taxa(taxa_sums(ps.c) > 0, ps.c)
ps.c.h <- prune_taxa(taxa_sums(ps.c.h) > 0, ps.c.h)
ps.c.n <- prune_taxa(taxa_sums(ps.c.n) > 0, ps.c.n)
rich.c <- merge(estimate_richness(ps.c, measures = c('Observed', 'InvSimpson')),
                sample_data(ps.c), by = 'row.names')
rich.t <- merge(estimate_richness(ps.t, measures = c('Observed', 'InvSimpson')),
                sample_data(ps.t), by = 'row.names')
rich.c.h <- merge(estimate_richness(ps.c.h, measures = c('Observed', 'InvSimpson')),
                  sample_data(ps.c.h), by = 'row.names')
rich.c.n <- merge(estimate_richness(ps.c.n, measures = c('Observed', 'InvSimpson')),
                  sample_data(ps.c.n), by = 'row.names')
rich.t.h <- merge(estimate_richness(ps.t.h, measures = c('Observed', 'InvSimpson')),
                  sample_data(ps.t.h), by = 'row.names')
rich.t.n <- merge(estimate_richness(ps.t.n, measures = c('Observed', 'InvSimpson')),
                  sample_data(ps.t.n), by = 'row.names')
# test for significant differences in richness
lapply(split.data.frame(rich.c.h, rich.c.h$site, drop = T),
       function(x) { summary(glht(glm(
         Observed ~ group, family = gaussian, data = x), linfct = mcp(group = 'Tukey'))) } )
```


```
$Stomach

     Simultaneous Tests for General Linear Hypotheses

Multiple Comparisons of Means: Tukey Contrasts


Fit: glm(formula = Observed ~ group, family = gaussian, data = x)

Linear Hypotheses:
               Estimate Std. Error z value Pr(>|z|)  
MIX - LAC == 0   20.933      7.798   2.685   0.0194 *
CSS - LAC == 0    3.548      5.497   0.645   0.7918  
CSS - MIX == 0  -17.386      8.036  -2.163   0.0755 .
---
Signif. codes:  0 ‘***’ 0.001 ‘**’ 0.01 ‘*’ 0.05 ‘.’ 0.1 ‘ ’ 1
(Adjusted p values reported -- single-step method)


$Ileum

     Simultaneous Tests for General Linear Hypotheses

Multiple Comparisons of Means: Tukey Contrasts


Fit: glm(formula = Observed ~ group, family = gaussian, data = x)

Linear Hypotheses:
               Estimate Std. Error z value Pr(>|z|)
MIX - LAC == 0  -2.6569     7.0435  -0.377    0.924
CSS - LAC == 0  -2.4664     5.3533  -0.461    0.888
CSS - MIX == 0   0.1905     7.2377   0.026    1.000
(Adjusted p values reported -- single-step method)


$Colon

     Simultaneous Tests for General Linear Hypotheses

Multiple Comparisons of Means: Tukey Contrasts


Fit: glm(formula = Observed ~ group, family = gaussian, data = x)

Linear Hypotheses:
               Estimate Std. Error z value Pr(>|z|)   
MIX - LAC == 0    6.733     13.174   0.511  0.86464   
CSS - LAC == 0  -30.171     10.135  -2.977  0.00797 **
CSS - MIX == 0  -36.905     13.308  -2.773  0.01501 * 
---
Signif. codes:  0 ‘***’ 0.001 ‘**’ 0.01 ‘*’ 0.05 ‘.’ 0.1 ‘ ’ 1
(Adjusted p values reported -- single-step method)
```


```
lapply(split.data.frame(rich.c.n, rich.c.n$site, drop = T),
       function(x) { summary(glht(glm(
         Observed ~ group, family = gaussian, data = x), linfct = mcp(group = 'Tukey'))) } )
```


```
$Stomach

     Simultaneous Tests for General Linear Hypotheses

Multiple Comparisons of Means: Tukey Contrasts


Fit: glm(formula = Observed ~ group, family = gaussian, data = x)

Linear Hypotheses:
               Estimate Std. Error z value Pr(>|z|)
MIX - LAC == 0   -6.467     12.378  -0.522    0.859
CSS - LAC == 0   -8.467     11.157  -0.759    0.725
CSS - MIX == 0   -2.000      9.283  -0.215    0.974
(Adjusted p values reported -- single-step method)


$Ileum

     Simultaneous Tests for General Linear Hypotheses

Multiple Comparisons of Means: Tukey Contrasts


Fit: glm(formula = Observed ~ group, family = gaussian, data = x)

Linear Hypotheses:
               Estimate Std. Error z value Pr(>|z|)
MIX - LAC == 0     5.00      13.36   0.374    0.925
CSS - LAC == 0     3.60      12.04   0.299    0.951
CSS - MIX == 0    -1.40      10.02  -0.140    0.989
(Adjusted p values reported -- single-step method)


$Colon

     Simultaneous Tests for General Linear Hypotheses

Multiple Comparisons of Means: Tukey Contrasts


Fit: glm(formula = Observed ~ group, family = gaussian, data = x)

Linear Hypotheses:
               Estimate Std. Error z value Pr(>|z|)
MIX - LAC == 0  13.4000    12.9046   1.038    0.548
CSS - LAC == 0  -0.9167    11.4062  -0.080    0.996
CSS - MIX == 0 -14.3167     9.4058  -1.522    0.276
(Adjusted p values reported -- single-step method)
```


```
lapply(split.data.frame(rich.t.h, rich.t.h$site, drop = T),
       function(x) { summary(glht(glm(
         Observed ~ group, family = gaussian, data = x), linfct = mcp(group = 'Tukey'))) } )
```


```
$Ileum

     Simultaneous Tests for General Linear Hypotheses

Multiple Comparisons of Means: Tukey Contrasts


Fit: glm(formula = Observed ~ group, family = gaussian, data = x)

Linear Hypotheses:
               Estimate Std. Error z value Pr(>|z|)
MIX - LAC == 0   -4.071      8.421  -0.484    0.878
CSS - LAC == 0   -5.110      6.647  -0.769    0.720
CSS - MIX == 0   -1.038      8.517  -0.122    0.992
(Adjusted p values reported -- single-step method)


$Colon

     Simultaneous Tests for General Linear Hypotheses

Multiple Comparisons of Means: Tukey Contrasts


Fit: glm(formula = Observed ~ group, family = gaussian, data = x)

Linear Hypotheses:
               Estimate Std. Error z value Pr(>|z|)  
MIX - LAC == 0   -15.39      19.70  -0.781   0.7120  
CSS - LAC == 0   -34.64      15.58  -2.224   0.0659 .
CSS - MIX == 0   -19.25      20.90  -0.921   0.6237  
---
Signif. codes:  0 ‘***’ 0.001 ‘**’ 0.01 ‘*’ 0.05 ‘.’ 0.1 ‘ ’ 1
(Adjusted p values reported -- single-step method)
```


```
lapply(split.data.frame(rich.t.n, rich.t.n$site, drop = T),
       function(x) { summary(glht(glm(
         Observed ~ group, family = gaussian, data = x), linfct = mcp(group = 'Tukey'))) } )
```


```
$Ileum

     Simultaneous Tests for General Linear Hypotheses

Multiple Comparisons of Means: Tukey Contrasts


Fit: glm(formula = Observed ~ group, family = gaussian, data = x)

Linear Hypotheses:
               Estimate Std. Error z value Pr(>|z|)
MIX - LAC == 0     0.80      12.09   0.066    0.998
CSS - LAC == 0     8.25      10.68   0.772    0.716
CSS - MIX == 0     7.45       8.81   0.846    0.671
(Adjusted p values reported -- single-step method)


$Colon

     Simultaneous Tests for General Linear Hypotheses

Multiple Comparisons of Means: Tukey Contrasts


Fit: glm(formula = Observed ~ group, family = gaussian, data = x)

Linear Hypotheses:
               Estimate Std. Error z value Pr(>|z|)  
MIX - LAC == 0    32.27      14.61   2.208   0.0683 .
CSS - LAC == 0    13.12      13.03   1.007   0.5684  
CSS - MIX == 0   -19.15      10.79  -1.774   0.1753  
---
Signif. codes:  0 ‘***’ 0.001 ‘**’ 0.01 ‘*’ 0.05 ‘.’ 0.1 ‘ ’ 1
(Adjusted p values reported -- single-step method)
```


```
# test for significant differences in alpha diversity
lapply(split.data.frame(rich.c.h, rich.c.h$site, drop = T),
       function(x) { summary(glht(glm(InvSimpson ~ group, family = gaussian, data = x),
                                  linfct = mcp(group = 'Tukey'))) } )
```


```
$Stomach

     Simultaneous Tests for General Linear Hypotheses

Multiple Comparisons of Means: Tukey Contrasts


Fit: glm(formula = InvSimpson ~ group, family = gaussian, data = x)

Linear Hypotheses:
               Estimate Std. Error z value Pr(>|z|)
MIX - LAC == 0   0.1562     0.8018   0.195    0.979
CSS - LAC == 0  -0.2259     0.5652  -0.400    0.914
CSS - MIX == 0  -0.3821     0.8263  -0.462    0.887
(Adjusted p values reported -- single-step method)


$Ileum

     Simultaneous Tests for General Linear Hypotheses

Multiple Comparisons of Means: Tukey Contrasts


Fit: glm(formula = InvSimpson ~ group, family = gaussian, data = x)

Linear Hypotheses:
                Estimate Std. Error z value Pr(>|z|)
MIX - LAC == 0  0.068477   0.419920   0.163    0.985
CSS - LAC == 0  0.009993   0.319150   0.031    0.999
CSS - MIX == 0 -0.058483   0.431497  -0.136    0.990
(Adjusted p values reported -- single-step method)


$Colon

     Simultaneous Tests for General Linear Hypotheses

Multiple Comparisons of Means: Tukey Contrasts


Fit: glm(formula = InvSimpson ~ group, family = gaussian, data = x)

Linear Hypotheses:
               Estimate Std. Error z value Pr(>|z|)
MIX - LAC == 0  -1.3375     0.9592  -1.394    0.341
CSS - LAC == 0  -1.2239     0.7379  -1.659    0.219
CSS - MIX == 0   0.1136     0.9690   0.117    0.992
(Adjusted p values reported -- single-step method)
```


```
lapply(split.data.frame(rich.c.n, rich.c.n$site, drop = T),
       function(x) { summary(glht(glm(InvSimpson ~ group, family = gaussian, data = x),
                                  linfct = mcp(group = 'Tukey'))) } )
```


```
$Stomach

     Simultaneous Tests for General Linear Hypotheses

Multiple Comparisons of Means: Tukey Contrasts


Fit: glm(formula = InvSimpson ~ group, family = gaussian, data = x)

Linear Hypotheses:
               Estimate Std. Error z value Pr(>|z|)
MIX - LAC == 0   0.4667     1.0867   0.429    0.902
CSS - LAC == 0  -0.2782     0.9795  -0.284    0.956
CSS - MIX == 0  -0.7449     0.8150  -0.914    0.628
(Adjusted p values reported -- single-step method)


$Ileum

     Simultaneous Tests for General Linear Hypotheses

Multiple Comparisons of Means: Tukey Contrasts


Fit: glm(formula = InvSimpson ~ group, family = gaussian, data = x)

Linear Hypotheses:
               Estimate Std. Error z value Pr(>|z|)
MIX - LAC == 0 -0.04436    0.62372  -0.071    0.997
CSS - LAC == 0  0.11509    0.56222   0.205    0.977
CSS - MIX == 0  0.15944    0.46779   0.341    0.937
(Adjusted p values reported -- single-step method)


$Colon

     Simultaneous Tests for General Linear Hypotheses

Multiple Comparisons of Means: Tukey Contrasts


Fit: glm(formula = InvSimpson ~ group, family = gaussian, data = x)

Linear Hypotheses:
               Estimate Std. Error z value Pr(>|z|)
MIX - LAC == 0   0.4647     0.8794   0.528    0.855
CSS - LAC == 0   0.7437     0.7773   0.957    0.600
CSS - MIX == 0   0.2790     0.6410   0.435    0.899
(Adjusted p values reported -- single-step method)
```


```
lapply(split.data.frame(rich.t.h, rich.t.h$site, drop = T),
       function(x) { summary(glht(glm(InvSimpson ~ group, family = gaussian, data = x),
                                  linfct = mcp(group = 'Tukey'))) } )
```


```
$Ileum

     Simultaneous Tests for General Linear Hypotheses

Multiple Comparisons of Means: Tukey Contrasts


Fit: glm(formula = InvSimpson ~ group, family = gaussian, data = x)

Linear Hypotheses:
               Estimate Std. Error z value Pr(>|z|)
MIX - LAC == 0  -0.8404     0.8700  -0.966    0.596
CSS - LAC == 0  -0.7066     0.6868  -1.029    0.556
CSS - MIX == 0   0.1338     0.8800   0.152    0.987
(Adjusted p values reported -- single-step method)


$Colon

     Simultaneous Tests for General Linear Hypotheses

Multiple Comparisons of Means: Tukey Contrasts


Fit: glm(formula = InvSimpson ~ group, family = gaussian, data = x)

Linear Hypotheses:
               Estimate Std. Error z value Pr(>|z|)
MIX - LAC == 0  -1.5774     1.3292  -1.187    0.457
CSS - LAC == 0  -2.1215     1.0508  -2.019    0.106
CSS - MIX == 0  -0.5441     1.4098  -0.386    0.920
(Adjusted p values reported -- single-step method)
```


```
lapply(split.data.frame(rich.t.n, rich.t.n$site, drop = T),
       function(x) { summary(glht(glm(InvSimpson ~ group, family = gaussian, data = x),
                                  linfct = mcp(group = 'Tukey'))) } )
```


```
$Ileum

     Simultaneous Tests for General Linear Hypotheses

Multiple Comparisons of Means: Tukey Contrasts


Fit: glm(formula = InvSimpson ~ group, family = gaussian, data = x)

Linear Hypotheses:
               Estimate Std. Error z value Pr(>|z|)
MIX - LAC == 0  -0.3234     0.6888  -0.470    0.884
CSS - LAC == 0   0.5729     0.6089   0.941    0.610
CSS - MIX == 0   0.8963     0.5021   1.785    0.171
(Adjusted p values reported -- single-step method)


$Colon

     Simultaneous Tests for General Linear Hypotheses

Multiple Comparisons of Means: Tukey Contrasts


Fit: glm(formula = InvSimpson ~ group, family = gaussian, data = x)

Linear Hypotheses:
               Estimate Std. Error z value Pr(>|z|)
MIX - LAC == 0  0.29333    0.83623   0.351    0.933
CSS - LAC == 0  0.20769    0.74582   0.278    0.958
CSS - MIX == 0 -0.08564    0.61760  -0.139    0.989
(Adjusted p values reported -- single-step method)
```


```
# plot Observed OTUs (All-fed pigs together) by formula group and by NEC/healthy
#   contents
gg1 <- ggplot(rich.c, aes(x = site, y = Observed, color = group)) +
  geom_boxplot(lwd = 1, position = position_dodge(width = 0.85), width = 0.7, outlier.shape = NA) +
  geom_point(shape = 19, position = position_jitterdodge(jitter.width = 0.15, dodge.width = 0.85)) +
  scale_colour_manual(values = group_colors) +
  labs(list(title = 'Obs, All-fed, Contents', x = element_blank(), y = element_blank())) +
  ylim(0, 150)
gg2 <- ggplot(rich.c, aes(x = site, y = Observed, color = hist.nec)) +
  geom_boxplot(lwd = 1, position = position_dodge(width = 0.85), width = 0.7, outlier.shape = NA) +
  geom_point(shape = 19, position = position_jitterdodge(jitter.width = 0.15, dodge.width = 0.85)) +
  scale_colour_manual(values = hn_colors) + ylim(0, 150) +
  labs(list(title = 'Obs, All-fed, Contents', x = element_blank(), y = element_blank())) 
grid.arrange(gg1, gg2, ncol = 2)
```


```
#   tissue
gg1 <- ggplot(rich.t, aes(x = site, y = Observed, color = group)) +
  geom_boxplot(lwd = 1, position = position_dodge(width = 0.85), width = 0.7, outlier.shape = NA) +
  geom_point(shape = 19, position = position_jitterdodge(jitter.width = 0.15, dodge.width = 0.85)) +
  scale_colour_manual(values = group_colors) +
  labs(list(title = 'Obs, All-fed, Tissue Mucosa', x = element_blank(), y = element_blank())) +
  ylim(0, 150)
gg2 <- ggplot(rich.t, aes(x = site, y = Observed, color = hist.nec)) +
  geom_boxplot(lwd = 1, position = position_dodge(width = 0.85), width = 0.7, outlier.shape = NA) +
  geom_point(shape = 19, position = position_jitterdodge(jitter.width = 0.15, dodge.width = 0.85)) +
  scale_colour_manual(values = hn_colors) + ylim(0, 150) +
  labs(list(title = 'Obs, All-fed, Tissue Mucosa', x = element_blank(), y = element_blank())) 
grid.arrange(gg1, gg2, ncol = 2)
```


```
# test Observed OTUs (All-fed pigs together) by formula group and by NEC/healthy
#   contents
lapply(split.data.frame(rich.c, rich.c$site, drop = T),
       function(x) { summary(glht(glm(
         Observed ~ group, family = gaussian, data = x), linfct = mcp(group = 'Tukey'))) } )
```


```
$Stomach

     Simultaneous Tests for General Linear Hypotheses

Multiple Comparisons of Means: Tukey Contrasts


Fit: glm(formula = Observed ~ group, family = gaussian, data = x)

Linear Hypotheses:
               Estimate Std. Error z value Pr(>|z|)
MIX - LAC == 0   7.9476     6.4394   1.234    0.430
CSS - LAC == 0  -0.9107     5.0080  -0.182    0.982
CSS - MIX == 0  -8.8583     6.3083  -1.404    0.336
(Adjusted p values reported -- single-step method)


$Ileum

     Simultaneous Tests for General Linear Hypotheses

Multiple Comparisons of Means: Tukey Contrasts


Fit: glm(formula = Observed ~ group, family = gaussian, data = x)

Linear Hypotheses:
               Estimate Std. Error z value Pr(>|z|)
MIX - LAC == 0   0.8773     5.9105   0.148    0.988
CSS - LAC == 0   0.1083     4.7672   0.023    1.000
CSS - MIX == 0  -0.7689     5.7330  -0.134    0.990
(Adjusted p values reported -- single-step method)


$Colon

     Simultaneous Tests for General Linear Hypotheses

Multiple Comparisons of Means: Tukey Contrasts


Fit: glm(formula = Observed ~ group, family = gaussian, data = x)

Linear Hypotheses:
               Estimate Std. Error z value Pr(>|z|)   
MIX - LAC == 0    4.121      9.364   0.440  0.89805   
CSS - LAC == 0  -22.449      7.502  -2.992  0.00777 **
CSS - MIX == 0  -26.570      8.800  -3.019  0.00719 **
---
Signif. codes:  0 ‘***’ 0.001 ‘**’ 0.01 ‘*’ 0.05 ‘.’ 0.1 ‘ ’ 1
(Adjusted p values reported -- single-step method)
```


```
lapply(split.data.frame(rich.c, rich.c$site, drop = T),
       function(x) { summary(glht(glm(
         Observed ~ hist.nec, family = gaussian, data = x), linfct = mcp(hist.nec = 'Tukey'))) } )
```


```
$Stomach

     Simultaneous Tests for General Linear Hypotheses

Multiple Comparisons of Means: Tukey Contrasts


Fit: glm(formula = Observed ~ hist.nec, family = gaussian, data = x)

Linear Hypotheses:
                   Estimate Std. Error z value Pr(>|z|)  
NEC - Healthy == 0   -8.671      4.718  -1.838   0.0661 .
---
Signif. codes:  0 ‘***’ 0.001 ‘**’ 0.01 ‘*’ 0.05 ‘.’ 0.1 ‘ ’ 1
(Adjusted p values reported -- single-step method)


$Ileum

     Simultaneous Tests for General Linear Hypotheses

Multiple Comparisons of Means: Tukey Contrasts


Fit: glm(formula = Observed ~ hist.nec, family = gaussian, data = x)

Linear Hypotheses:
                   Estimate Std. Error z value Pr(>|z|)
NEC - Healthy == 0    4.929      4.432   1.112    0.266
(Adjusted p values reported -- single-step method)


$Colon

     Simultaneous Tests for General Linear Hypotheses

Multiple Comparisons of Means: Tukey Contrasts


Fit: glm(formula = Observed ~ hist.nec, family = gaussian, data = x)

Linear Hypotheses:
                   Estimate Std. Error z value Pr(>|z|)
NEC - Healthy == 0   -5.886      7.575  -0.777    0.437
(Adjusted p values reported -- single-step method)
```


```
#   tissue
lapply(split.data.frame(rich.t, rich.t$site, drop = T),
       function(x) { summary(glht(glm(
         Observed ~ group, family = gaussian, data = x), linfct = mcp(group = 'Tukey'))) } )
```


```
$Ileum

     Simultaneous Tests for General Linear Hypotheses

Multiple Comparisons of Means: Tukey Contrasts


Fit: glm(formula = Observed ~ group, family = gaussian, data = x)

Linear Hypotheses:
               Estimate Std. Error z value Pr(>|z|)
MIX - LAC == 0   -4.797      6.476  -0.741    0.738
CSS - LAC == 0   -1.906      5.261  -0.362    0.930
CSS - MIX == 0    2.891      6.055   0.477    0.881
(Adjusted p values reported -- single-step method)


$Colon

     Simultaneous Tests for General Linear Hypotheses

Multiple Comparisons of Means: Tukey Contrasts


Fit: glm(formula = Observed ~ group, family = gaussian, data = x)

Linear Hypotheses:
               Estimate Std. Error z value Pr(>|z|)  
MIX - LAC == 0   -7.615     13.582  -0.561   0.8400  
CSS - LAC == 0  -26.741     11.014  -2.428   0.0399 *
CSS - MIX == 0  -19.126     13.378  -1.430   0.3236  
---
Signif. codes:  0 ‘***’ 0.001 ‘**’ 0.01 ‘*’ 0.05 ‘.’ 0.1 ‘ ’ 1
(Adjusted p values reported -- single-step method)
```


```
lapply(split.data.frame(rich.t, rich.t$site, drop = T),
       function(x) { summary(glht(glm(
         Observed ~ hist.nec, family = gaussian, data = x), linfct = mcp(hist.nec = 'Tukey'))) } )
```


```
$Ileum

     Simultaneous Tests for General Linear Hypotheses

Multiple Comparisons of Means: Tukey Contrasts


Fit: glm(formula = Observed ~ hist.nec, family = gaussian, data = x)

Linear Hypotheses:
                   Estimate Std. Error z value Pr(>|z|)
NEC - Healthy == 0   -2.668      4.707  -0.567    0.571
(Adjusted p values reported -- single-step method)


$Colon

     Simultaneous Tests for General Linear Hypotheses

Multiple Comparisons of Means: Tukey Contrasts


Fit: glm(formula = Observed ~ hist.nec, family = gaussian, data = x)

Linear Hypotheses:
                   Estimate Std. Error z value Pr(>|z|)
NEC - Healthy == 0   -14.36      10.66  -1.347    0.178
(Adjusted p values reported -- single-step method)
```


```
# get median values of observed OTUs for colon samples
with(rich.c[rich.c$site == 'Colon', ], aggregate(Observed ~ hist.nec:group, FUN=median))
```


```
rm(rich.c, rich.c.h, rich.c.n, rich.t, rich.t.h, rich.t.n, gg1, gg2, sample_sum_df, 
   sdata, biom_file, mapping_file)
```

## Overview - class level


```
# subsample to even read depth
ps.t.r <- rarefy_even_depth(ps.t, rngseed = 2501, replace = FALSE)
```


```
`set.seed(2501)` was used to initialize repeatable random subsampling.
Please record this for your records so others can reproduce.
Try `set.seed(2501); .Random.seed` for the full vector
...
480OTUs were removed because they are no longer 
present in any sample after random subsampling

...
```


```
ps.c.r <- rarefy_even_depth(ps.c, rngseed = 2501, replace = FALSE)
```


```
`set.seed(2501)` was used to initialize repeatable random subsampling.
Please record this for your records so others can reproduce.
Try `set.seed(2501); .Random.seed` for the full vector
...
205OTUs were removed because they are no longer 
present in any sample after random subsampling

...
```


```
# subset by GI tract site
ps.list <- list('Ileum Tissue' = subset_samples(ps.t.r, site == 'Ileum'),
                'Colon Tissue' = subset_samples(ps.t.r, site == 'Colon'),
                'Stomach Contents' = subset_samples(ps.c.r, site == 'Stomach'),
                'Ileum Contents' = subset_samples(ps.c.r, site == 'Ileum'),
                'Colon Contents' = subset_samples(ps.c.r, site == 'Colon'))
# transform abundances from absolute to relative (proportions)
ps.list <- llply(ps.list, function(x) {
  transform_sample_counts(x, function(y) y / sum(y)) })
ps.t.r <- transform_sample_counts(ps.t.r, function(x) x / sum(x))
ps.c.r <- transform_sample_counts(ps.c.r, function(x) x / sum(x))
# agglomerate OTUs by class
ps.list.class <- llply(ps.list, function(x) { tax_glom(x, 'Class') })
otus <- llply(ps.list.class, function(x) { row.names(tax_table(x)) })
keep <- llply(names(ps.list.class), function(x) {
  row.names(tax_table(ps.list.class[[x]]))[which(tax_table(ps.list.class[[x]])[ , 'Class'] 
                                                 %in% c('Gammaproteobacteria', 'Clostridia', 'Bacilli'))] })
# combine small taxa into 'Other'
names(keep) <- names(ps.list.class)
ps.list.class.o <- llply(names(ps.list.class), function(x) { 
  merge_taxa(ps.list.class[[x]], setdiff(otus[[x]], keep[[x]])) })
names(ps.list.class.o) <- names(ps.list.class)
for(x in seq_along(ps.list.class.o)) {
  tax_table(ps.list.class.o[[x]])[is.na(tax_table(ps.list.class.o[[x]])[ , 'Class']), 2:3] <- c('Other', 'Other')
}
rm(group.orders, keep, otus, x, ps.list.class.o)
```


```
object 'group.orders' not found
```

## Figures S3 and S4. Formula groups (all fed) - class level


```
# top N number of taxa to compare
topN <- 4
# prepare to plot top N "CLASS" in boxplots
psm.list.class <- lapply(ps.list.class, psmelt)
# get top N taxa ranked by decreasing mean abundance
means <- lapply(psm.list.class, function(x){ ddply(x, ~ Class,
                                                   function(y){ c(mean = mean(y$Abundance)) }) })
means <- lapply(means, function(x){ x[rev(order(x$mean)), ] })
topN.taxa <- lapply(means, function(x){ x[1:topN, 'Class'] })
topN.taxa <- lapply(topN.taxa, function(x) { topN.taxa[['Colon Contents']] }) # force same order
psm.list.class <- mapply(function(x, i){ x[x$Class %in% topN.taxa[[i]], ] }, psm.list.class, seq_along(psm.list.class),
                         SIMPLIFY = FALSE, USE.NAMES = TRUE)
# determine if any differences across groups
kw.pvals <- lapply(psm.list.class, function(x){ ddply(x, ~ Class, function(y) {
  c(prob = kruskal.test(data = y, Abundance ~ group)$p.value) }) })
for(i in seq_along(kw.pvals)) {
  kw.pvals[[i]]$prob <- p.adjust(kw.pvals[[i]]$prob, 'holm') # only care about top 3
}
# get labels for taxa which are different
sigs <- llply(names(kw.pvals), function(x) {kw.pvals[[x]]$prob <= 0.05})
names(sigs) <- names(kw.pvals)
sigs <- llply(names(sigs), function(x) { if(any(sigs[[x]])) kw.pvals[[x]][sigs[[x]], 'Class'] else NA })
names(sigs) <- names(kw.pvals)
for(i in seq_along(psm.list.class)) {
  plot <- ggplot(data.frame(psm.list.class[[i]]), aes(x = Class, y = Abundance, color = group)) + 
    geom_boxplot(lwd = 1, position = position_dodge(width = 0.75), width = 0.7,
                 outlier.shape = NA) +
    geom_point(shape = 19, position = position_jitterdodge(jitter.width = 0.15,
                                                           dodge.width = 0.75)) +
    scale_x_discrete(limits = unlist(topN.taxa[i])) +
    scale_y_sqrt(breaks = c(1, 0.64, 0.36, 0.16, 0.04, 0.0), limits = c(0, 1.1), labels = percent) +
    scale_colour_manual(values = group_colors) + 
    annotate('text', x = unlist(sigs[i]), na.rm = TRUE, y = 1.05, label = '*', size = 12) +
    theme(axis.text.x = element_text(angle = -45, hjust = 0, vjust = 1)) +
    labs(list(title = names(psm.list.class)[i], x = element_blank(), y = element_text('Relative Abundance'))) +
    theme(legend.position = 'right', legend.title = element_blank(), plot.margin = margin(10, 10, 10, 25))
  print(plot)
}
```


```
# test for difference in Bacilli between GI contents sites
sc <- psm.list.class$'Stomach Contents'
ic <- psm.list.class$'Ileum Contents'
cc <- psm.list.class$'Colon Contents'
bacilli.list <- list(sc[sc$Class == 'Bacilli', 'Abundance'], ic[ic$Class == 'Bacilli', 'Abundance'],
                     cc[cc$Class == 'Bacilli', 'Abundance'])
lapply(bacilli.list, median)
```


```
[[1]]
[1] 0.4404681

[[2]]
[1] 0.06911465

[[3]]
[1] 0.04901628
```


```
kruskal.test(bacilli.list)
```


```
    Kruskal-Wallis rank sum test

data:  bacilli.list
Kruskal-Wallis chi-squared = 65.042, df = 2, p-value = 7.522e-15
```


```
rm(bacilli.list, kw.pvals, means, plot, psm.list.class, sigs, topN, topN.taxa,
   sc, ic, cc, i)
```

## Figures S5 and S6. Healthy vs NEC (all fed) - class level


```
# top N number of taxa to compare
topN <- 4
# prepare to plot top N "CLASS" in boxplots
psm.list.class <- lapply(ps.list.class, psmelt)
# get top N taxa ranked by decreasing mean abundance
means <- lapply(psm.list.class, function(x){ ddply(x, ~ Class,
                                                   function(y){ c(mean = mean(y$Abundance)) }) })
means <- lapply(means, function(x){ x[rev(order(x$mean)), ] })
topN.taxa <- lapply(means, function(x){ x[1:topN, 'Class'] })
topN.taxa <- lapply(topN.taxa, function(x) { topN.taxa[['Colon Contents']] }) # force same order
psm.list.class <- mapply(function(x, i){ x[x$Class %in% topN.taxa[[i]], ] }, psm.list.class, seq_along(psm.list.class),
                         SIMPLIFY = FALSE, USE.NAMES = TRUE)
# determine if any differences across groups
mwu.pvals <- lapply(psm.list.class, function(x){ ddply(x, ~ Class, function(y) {
  c(prob = wilcox.test(data = y, Abundance ~ hist.nec, exact = FALSE)$p.value) }) })
for(i in seq_along(mwu.pvals)) {
  mwu.pvals[[i]]$prob <- p.adjust(mwu.pvals[[i]]$prob, 'holm')
}
# get labels for taxa which are different
sigs <- llply(names(mwu.pvals), function(x) {mwu.pvals[[x]]$prob <= 0.05})
names(sigs) <- names(mwu.pvals)
sigs <- llply(names(sigs), function(x) { if(any(sigs[[x]])) mwu.pvals[[x]][sigs[[x]], 'Class'] else NA })
names(sigs) <- names(mwu.pvals)
for(i in seq_along(psm.list.class)) {
  plot <- ggplot(data.frame(psm.list.class[[i]]), aes(x = Class, y = Abundance, color = hist.nec)) + 
    geom_boxplot(lwd = 1, position = position_dodge(width = 0.75), width = 0.7,
                 outlier.shape = NA) +
    geom_point(shape = 19, position = position_jitterdodge(jitter.width = 0.15,
                                                           dodge.width = 0.75)) +
    scale_x_discrete(limits = unlist(topN.taxa[i])) +
    scale_y_sqrt(breaks = c(1, 0.64, 0.36, 0.16, 0.04, 0.0), limits = c(0, 1.1), labels = percent) +
    scale_colour_manual(values = hn_colors) + 
    annotate('text', x = sigs[[i]], na.rm = TRUE, y = 1.05, label = '*', size = 12) +
    theme(axis.text.x = element_text(angle = -45, hjust = 0, vjust = 1)) +
    labs(list(title = names(psm.list.class)[i], x = element_blank(), y = element_text('Relative Abundance'))) +
    theme(legend.position = 'right', legend.title = element_blank(), plot.margin = margin(10, 10, 10, 25))
  print(plot)
}
```


```
rm(means, mwu.pvals, plot, psm.list.class, sigs, topN, topN.taxa, i)
```

## Figures 3 and S7. Diet groups (all fed) - genus level


```
# top N number of taxa to compare
topN <- 7
# agglomerate OTUs by genus
ps.list.genus <- llply(ps.list, function(x) { tax_glom(x, 'Genus') })
# melt data to long format
psm.list.genus <- llply(ps.list.genus, function(x) psmelt(x))
# get top N taxa ranked by decreasing median abundance
medians <- lapply(psm.list.genus, function(x){ ddply(x, ~ Genus,
                                                     function(y){ c(median = median(y$Abundance)) }) })
medians <- lapply(medians, function(x){ x[rev(order(x$median)), ] })
topN.taxa <- lapply(medians, function(x){ x[1:topN, 'Genus'] })
topN.taxa <- lapply(topN.taxa, function(x) { topN.taxa[['Colon Contents']] }) # force same order
psm.list.genus <- mapply(function(x, i){ x[x$Genus %in% topN.taxa[[i]], ] }, psm.list.genus, seq_along(psm.list.genus),
                         SIMPLIFY = FALSE, USE.NAMES = TRUE)
# determine if any differences across groups
kw.pvals <- lapply(psm.list.genus, function(x){ ddply(x, ~ Genus, function(y) {
  c(prob = kruskal.test(data = y, Abundance ~ group)$p.value) }) })
for(i in seq_along(kw.pvals)) {
  kw.pvals[[i]]$prob <- p.adjust(kw.pvals[[i]]$prob, 'holm')
}
# get labels for taxa which are different
sigs <- llply(names(kw.pvals), function(x) {kw.pvals[[x]]$prob <= 0.05})
names(sigs) <- names(kw.pvals)
sigs <- llply(names(sigs), function(x) { if(any(sigs[[x]])) kw.pvals[[x]][sigs[[x]], 'Genus'] else NA })
names(sigs) <- names(kw.pvals)
# make boxplots
for(i in seq_along(psm.list.genus)) {
  plot <- ggplot(data.frame(psm.list.genus[[i]]), aes(x = Genus, y = Abundance, color = group)) + 
    geom_boxplot(lwd = 1, position = position_dodge(width = 0.75), width = 0.7,
                 outlier.shape = NA) +
    geom_point(shape = 19, position = position_jitterdodge(jitter.width = 0.15,
                                                           dodge.width = 0.75)) +
    scale_x_discrete(limits = topN.taxa[[i]]) +
    scale_y_sqrt(breaks = c(1, 0.64, 0.36, 0.16, 0.04, 0.0), limits = c(0, 1.1), labels = percent) +
    scale_colour_manual(values = diet_colors) + 
    annotate('text', x = unlist(sigs[i]), na.rm = TRUE, y = 1.05, label = '*', size = 12) +
    theme(axis.text.x = element_text(angle = -45, hjust = 0, vjust = 1)) +
    labs(list(title = names(psm.list.genus)[i], x = element_blank(), y = element_text('Relative Abundance'))) +
    theme(legend.position = 'right', legend.title = element_blank(), plot.margin = margin(10, 10, 10, 25))
  print(plot)
}
```


```
# find median relative abundance of Enterobacteriaceae
ic <- psm.list.genus$'Ileum Contents'
cc <- psm.list.genus$'Colon Contents'
ent.list <- list(ic[ic$Genus == 'Enterobacteriaceae unclassified', 'Abundance'],
                 cc[cc$Genus == 'Enterobacteriaceae unclassified', 'Abundance'])
median(unlist(ent.list))
```


```
[1] 0.6613806
```


```
rm(ent.list, i, kw.pvals, medians, plot, psm.list.genus, sigs, topN, topN.taxa, ic, cc)
```

## Figures 4 and S8. Healthy vs NEC (all fed) - Genus level


```
# top N number of taxa to compare
topN <- 7
# prepare to plot top N "GENUS" in boxplots
psm.list.genus <- lapply(ps.list.genus, psmelt)
# get top N taxa ranked by decreasing median abundance
medians <- lapply(psm.list.genus, function(x){ ddply(x, ~ Genus,
                                                     function(y){ c(median = median(y$Abundance)) }) })
medians <- lapply(medians, function(x){ x[rev(order(x$median)), ] })
topN.taxa <- lapply(medians, function(x){ x[1:topN, 'Genus'] })
topN.taxa <- lapply(topN.taxa, function(x) { topN.taxa[['Colon Contents']] }) # force same order
psm.list.genus <- mapply(function(x, i){ x[x$Genus %in% topN.taxa[[i]], ] }, psm.list.genus, seq_along(psm.list.genus),
                         SIMPLIFY = FALSE, USE.NAMES = TRUE)
# determine if any differences across groups
mwu.pvals <- lapply(psm.list.genus, function(x){ ddply(x, ~ Genus, function(y) {
  c(prob = wilcox.test(data = y, Abundance ~ hist.nec, exact = FALSE)$p.value) }) })
for(i in seq_along(mwu.pvals)) {
  mwu.pvals[[i]]$prob <- p.adjust(mwu.pvals[[i]]$prob, 'holm')
}
# get labels for taxa which are different
sigs <- llply(names(mwu.pvals), function(x) {mwu.pvals[[x]]$prob <= 0.05})
names(sigs) <- names(mwu.pvals)
sigs <- llply(names(sigs), function(x) { if(any(sigs[[x]])) mwu.pvals[[x]][sigs[[x]], 'Genus'] else NA })
names(sigs) <- names(mwu.pvals)
for(i in seq_along(psm.list.genus)) {
  plot <- ggplot(data.frame(psm.list.genus[[i]]), aes(x = Genus, y = Abundance, color = hist.nec)) + 
    geom_boxplot(lwd = 1, position = position_dodge(width = 0.75), width = 0.7,
                 outlier.shape = NA) +
    geom_point(shape = 19, position = position_jitterdodge(jitter.width = 0.15,
                                                           dodge.width = 0.75)) +
    scale_x_discrete(limits = unlist(topN.taxa[i])) +
    scale_y_sqrt(breaks = c(1, 0.64, 0.36, 0.16, 0.04, 0.0), limits = c(0, 1.1), labels = percent) +
    scale_colour_manual(values = hn_colors) + 
    annotate('text', x = sigs[[i]], na.rm = TRUE, y = 1.05, label = '*', size = 12) +
    theme(axis.text.x = element_text(angle = -45, hjust = 0, vjust = 1)) +
    labs(list(title = names(psm.list.genus)[i], x = element_blank(), y = element_text('Relative Abundance'))) +
    theme(legend.position = 'right', legend.title = element_blank(), plot.margin = margin(10, 10, 10, 25))
  print(plot)
}
```


```
# measure difference in Clostridium sensu stricto between healthy/NEC
ic <- psm.list.genus$'Ileum Contents'
with(ic[ic$Genus == 'Clostridium sensu stricto', c('Abundance', 'hist.nec')], 
     aggregate(Abundance ~ hist.nec, FUN=median))
```


```
# measure difference in Enterobacteriaceae between healthy/NEC
sc <- psm.list.genus$'Stomach Contents'
with(sc[sc$Genus == 'Enterobacteriaceae unclassified', c('Abundance', 'hist.nec')],
     aggregate(Abundance ~ hist.nec, FUN=median))
```


```
with(ic[ic$Genus == 'Enterobacteriaceae unclassified', c('Abundance', 'hist.nec')],
     aggregate(Abundance ~ hist.nec, FUN=median))
```


```
rm(i, medians, mwu.pvals, plot, psm.list.genus, sigs, topN, topN.taxa, ic)
```

## Metabolites overview (all fed)


```
# load data
c.cmpds <- read.csv('cecal_compounds.csv', strip.white = T)
cecal <- read.csv('cecal.csv', strip.white = T)  # "COMP ID" uniquely identifies compounds
p.cmpds <- read.csv('plasma_compounds.csv', strip.white = T)
plasma <- read.csv('plasma.csv', strip.white = T)
pigs <- getMJNPigs(incl.new = F)
cecal <- merge(pigs, cecal)
plasma <- merge(pigs, plasma)
# get column names for compounds
c.compIDs <- names(cecal)[!(names(cecal) %in% c(names(pigs), 'mass.dry.g', 'bradford.protein'))]
p.compIDs <- names(plasma)[!(names(plasma) %in% names(pigs))]
# remove compounds that have no values (i.e. were only found in 'NEW' pigs)
#  cecal
c.compIDs.toFilter <- names(which(colSums(cecal[, c.compIDs], na.rm = T) == 0))
cecal <- cecal[!names(cecal) %in% c.compIDs.toFilter]
c.compIDs <- setdiff(c.compIDs, c.compIDs.toFilter)
#  plasma
p.compIDs.toFilter <- names(which(colSums(plasma[, p.compIDs], na.rm = T) == 0))
plasma <- plasma[!names(plasma) %in% p.compIDs.toFilter]
p.compIDs <- setdiff(p.compIDs, p.compIDs.toFilter)
# normalize concentration measurements for each cecal sample - divide by dry mass of sample
cecal[, c.compIDs] <- sweep(cecal[, c.compIDs], 1, cecal[, 'mass.dry.g'], '/')
# replace 0s and NA (missing values) w/ 1/2 column minimum
#  cecal
c.colmins <- apply(cecal[, c.compIDs], 2, function(x){ min(x, na.rm = T) })
c.nas <- which(is.na(cecal), arr.ind = T)
c.nas <- c.nas[which(c.nas[, 2] > ncol(cecal) - length(c.compIDs)), ]
cecal[c.nas] <- c.colmins[c.nas[, 2] - (ncol(cecal) - length(c.compIDs))] / 2
#  plasma
p.colmins <- apply(plasma[, p.compIDs], 2, function(x){ min(x, na.rm = T) })
p.nas <- which(is.na(plasma), arr.ind = T)
p.nas <- p.nas[which(p.nas[, 2] > ncol(plasma) - length(p.compIDs)), ]
plasma[p.nas] <- p.colmins[p.nas[, 2] - (ncol(plasma) - length(p.compIDs))] / 2
# save absolute values to use for calculating fold-change
c.abs <- cecal
p.abs <- plasma
# transform values by generalized log
cecal[, c.compIDs] <- apply(cecal[, c.compIDs], 2, function(x) { log2((x + sqrt(x^2 + 1)) / 2) })
plasma[, p.compIDs] <- apply(plasma[, p.compIDs], 2, function(x) { log2((x + sqrt(x^2 + 1)) / 2) })
# scale values by autoscaling (force each variable to have mean = 0 and sd = 1)
cecal[, c.compIDs] <- apply(cecal[, c.compIDs], 2, function(x) { (x - mean(x)) / sd(x) })
plasma[, p.compIDs] <- apply(plasma[, p.compIDs], 2, function(x) { (x - mean(x)) / sd(x) })
# change column names from 'c.#####' or 'p.#####' format to biochemical name
#  cecal
c.cmpds$colnamesID <- paste('c.', c.cmpds$comp.id, sep='')
c.comp.names <- as.character(c.cmpds$biochemical[na.omit(match(colnames(cecal), c.cmpds$colnamesID))])
colnames(cecal)[colnames(cecal) %in% c.compIDs] <- c.comp.names
colnames(c.abs)[colnames(c.abs) %in% c.compIDs] <- c.comp.names
# plasma
p.cmpds$colnamesID <- paste('p.', p.cmpds$comp.id, sep='')
p.comp.names <- as.character(p.cmpds$biochemical[na.omit(match(colnames(plasma), p.cmpds$colnamesID))])
colnames(plasma)[colnames(plasma) %in% p.compIDs] <- p.comp.names
colnames(p.abs)[colnames(p.abs) %in% p.compIDs] <- p.comp.names
c.fed <- droplevels(cecal[cecal$group %in% c('LAC', 'MIX', 'CSS'), ])
p.fed <- droplevels(plasma[plasma$group %in% c('LAC', 'MIX', 'CSS'), ])
# plasma
# build dataframe of from type II SS ANOVA for group and hist.nec
# this call to anova returns p-vals for group term
#   i.e. return the p-val for the group term after controlling for hist.nec
p.anova <- as.data.frame(apply(p.fed[, as.character(p.comp.names)], 2, 
                               function(x) anova(lm(x ~ p.fed$hist.nec * p.fed$group))$'Pr(>F)'[2]))
# this call to anova returns p-vals for hist.nec term and group:hist.nec interaction term
#   i.e. return the p-val for the hist.nec term and group:hist.nec interaction term 
#   after controlling for group
p.anova <- cbind(p.anova, t(apply(p.fed[, as.character(p.comp.names)], 2,
                                  function(x) anova(lm(x ~ p.fed$group * p.fed$hist.nec))$'Pr(>F)'[2:3])))
colnames(p.anova) <- c('group.p', 'hist.nec.p', 'itx.p')
# add median fold differences between Healthy and NEC
median.h <- apply(p.abs[p.abs$hist.nec == 'Healthy', row.names(p.anova)], 2, median, na.rm = T)
median.n <- apply(p.abs[p.abs$hist.nec == 'NEC', row.names(p.anova)], 2, median, na.rm = T)
p.anova$nec.fold.diff <- median.h / median.n
# add median fold differences between LAC and CSS
median.lac <- apply(p.abs[p.abs$group == 'LAC', row.names(p.anova)], 2, median, na.rm = T)
median.css <- apply(p.abs[p.abs$group == 'CSS', row.names(p.anova)], 2, median, na.rm = T)
p.anova$group.fold.diff <- median.lac / median.css
# control family-wise error rate for group and hist.nec using FDR
p.anova[ , c('group.p', 'hist.nec.p', 'itx.p')] <- apply(p.anova[ , c('group.p', 'hist.nec.p', 'itx.p')], 2, 
                                                         function(x) p.adjust(x, method = 'fdr'))
# count how many metabolites were found to be different at q < 0.05
#   count only metabolites significant for the main effect AND no interaction
count(p.anova$group.p < 0.01 & p.anova$itx.p > 0.01)
```


```
count(p.anova$hist.nec.p < 0.01 & p.anova$itx.p > 0.01)
```


```
count(p.anova$itx.p < 0.01)
```


```
# cecal
# build dataframe of from type II SS ANOVA for group and hist.nec
# this call to anova returns p-vals for group term
#   i.e. return the p-val for the group term after controlling for hist.nec
c.anova <- as.data.frame(apply(c.fed[, as.character(c.comp.names)], 2, 
                               function(x) anova(lm(x ~ c.fed$hist.nec * c.fed$group))$'Pr(>F)'[2]))
# this call to anova returns p-vals for hist.nec term and group:hist.nec interaction term
#   i.e. return the p-val for the hist.nec term and group:hist.nec interaction term 
#   after controlling for group
c.anova <- cbind(c.anova, t(apply(c.fed[, as.character(c.comp.names)], 2,
                                  function(x) anova(lm(x ~ c.fed$group * c.fed$hist.nec))$'Pr(>F)'[2:3])))
colnames(c.anova) <- c('group.p', 'hist.nec.p', 'itx.p')
# add median fold differences between Healthy and NEC
median.h <- apply(c.abs[c.abs$hist.nec == 'Healthy', row.names(c.anova)], 2, median, na.rm = T)
median.n <- apply(c.abs[c.abs$hist.nec == 'NEC', row.names(c.anova)], 2, median, na.rm = T)
c.anova$nec.fold.diff <- median.h / median.n
# add median fold differences between LAC and CSS
median.lac <- apply(c.abs[c.abs$group == 'LAC', row.names(c.anova)], 2, median, na.rm = T)
median.css <- apply(c.abs[c.abs$group == 'CSS', row.names(c.anova)], 2, median, na.rm = T)
c.anova$group.fold.diff <- median.lac / median.css
# control family-wise error rate for group and hist.nec using FDR
c.anova[ , c('group.p', 'hist.nec.p', 'itx.p')] <- apply(c.anova[ , c('group.p', 'hist.nec.p', 'itx.p')], 2, 
                                                         function(x) p.adjust(x, method = 'fdr'))
# count how many metabolites were found to be different at q < 0.05
#   count only metabolites significant for the main effect AND no interaction
count(c.anova$group.p < 0.01 & c.anova$itx.p > 0.01)
```


```
count(c.anova$hist.nec.p < 0.01 & c.anova$itx.p > 0.01)
```


```
count(c.anova$itx.p < 0.01)
```


```
rm(c.abs, p.abs, c.cmpds, p.cmpds, c.fed, p.fed, c.nas, p.nas, pigs, median.css,
   median.lac, median.h, median.n, p.colmins, c.colmins, c.comp.names, p.comp.names,
   c.compIDs, p.compIDs, c.compIDs.toFilter, p.compIDs.toFilter)
```


```
# Figure 6.  Cecal metabolites heatmap
# get metabolites to use for heatmap - use p-val cutoff and rank those within
#   the cutoff by decreasing effect size, then take top 50 (or all if < 50)
tmp <- c.anova[which(c.anova$hist.nec.p < 0.05), ]
c.metabs.plot <- row.names(tmp[order(abs(log2(tmp$nec.fold.diff)), decreasing = T), ]) # by effect size
c.metabs.plot <- c.metabs.plot[1 : ifelse(length(c.metabs.plot) < 50, length(c.metabs.plot), 50)]
rm(tmp)
# plot heatmap
c.mat <- t(as.matrix.data.frame(cecal[order(cecal$hist.nec, cecal$group), c.metabs.plot]))
colnames(c.mat) <- cecal[order(cecal$hist.nec, cecal$group), 'pigID']
rownames(c.mat) <- c.metabs.plot
annot.df <- cecal[, c('hist.nec', 'group')]
annot.df[, 'hist.nec'] <- as.character(annot.df[, 'hist.nec'])
annot.df[, 'group'] <- as.character(annot.df[, 'group'])
rownames(annot.df) <- cecal$pigID
ann.colors <- list(hist.nec = c('Healthy' = hn_colors[1], 'NEC' = hn_colors[2]),
                   group = c('LAC' = diet_colors[1], 'MIX' = diet_colors[2], 'CSS' = diet_colors[3]))
pheatmap(c.mat,
         cluster_rows = T, 
         cluster_cols = T,
         color = rev(colorRampPalette(brewer.pal(8, "RdBu"))(256)),
         fontsize_row = 7,
         annotation = annot.df,
         annotation_colors = ann.colors,
         clustering_distance_rows = 'euclidean',
         clustering_method = 'ward.D2',
         border_color = 'gray',
         scale = 'none',
         gaps_col = length(which(annot.df[, 'hist.nec'] == 'Healthy')),
         show_colnames = F,
         #main = 'Cecal',
         legend = F, annotation_legend = F,
         annotation_names_col = F)
```


```
rm(annot.df, c.mat, ann.colors, c.metabs.plot)
```


```
# Figure S9.  Plasma metabolites heatmap
# get metabolites to use for heatmap - use p-val cutoff and rank those within
#   the cutoff by decreasing effect size, then take top 50 (or all if < 50)
tmp <- p.anova[which(p.anova$hist.nec.p < 0.05), ]
p.metabs.plot <- row.names(tmp[order(abs(log2(tmp$nec.fold.diff)), decreasing = T), ]) # by effect size
p.metabs.plot <- p.metabs.plot[1 : ifelse(length(p.metabs.plot) < 50, length(p.metabs.plot), 50)]
rm(tmp)
# plot heatmap
p.mat <- t(as.matrix.data.frame(plasma[order(plasma$hist.nec, plasma$group), p.metabs.plot]))
colnames(p.mat) <- plasma[order(plasma$hist.nec, plasma$group), 'pigID']
rownames(p.mat) <- p.metabs.plot
annot.df <- plasma[, c('hist.nec', 'group')]
annot.df[, 'hist.nec'] <- as.character(annot.df[, 'hist.nec'])
annot.df[, 'group'] <- as.character(annot.df[, 'group'])
rownames(annot.df) <- plasma$pigID
ann.colors <- list(hist.nec = c('Healthy' = hn_colors[1], 'NEC' = hn_colors[2]),
                   group = c('LAC' = diet_colors[1], 'MIX' = diet_colors[2], 'CSS' = diet_colors[3]))
pheatmap(p.mat,
         cluster_rows = T, 
         cluster_cols = T,
         color = rev(colorRampPalette(brewer.pal(8, "RdBu"))(256)),
         fontsize_row = 7,
         annotation = annot.df,
         annotation_colors = ann.colors,
         clustering_distance_rows = 'euclidean',
         clustering_method = 'ward.D2',
         border_color = 'gray',
         scale = 'none',
         gaps_col = length(which(annot.df[, 'hist.nec'] == 'Healthy')),
         show_colnames = F,
         #main = 'plasma',
         legend = F, annotation_legend = F,
         annotation_names_col = F)
```


```
rm(annot.df, p.mat, ann.colors, p.metabs.plot)
```

## Figure 6. Selected plasma metabolites


```
# create plotting function
plotMetabolites <- function(plot.df, metabs, plot_groups) {
  for (plot.cmpd in metabs) {
    if(plot_groups == 'hist.nec') {
      plot_colors <- hn_colors 
    } else if(plot_groups == 'group') {
      plot_colors <- group_colors
    } else {
      plot_colors <- group_NEC_colors
    }
    plot <- ggplot(plot.df, aes(x = plot.df[ , plot_groups], y = plot.df[, plot.cmpd],
                                color = plot.df[ , plot_groups])) +
      geom_boxplot(lwd = 1, position = position_dodge(width = 0.85), width = 0.7,
                   outlier.shape = NA) +
      geom_point(shape = 19, position = position_jitterdodge(jitter.width = 0.15,
                                                             dodge.width = 0.85)) +
      scale_colour_manual(values = plot_colors) + theme(legend.position = 'none') +
      labs(list(title = plot.cmpd, x = element_blank(), y = 'Relative Concentration')) +
      theme(axis.text.x = element_text(angle = -30, hjust = 0)) + ylim(-2.75, 2.75)
    print(plot)
  }
}
# add factor to allow plotting by group-NEC
plasma <- cbind(plasma, 'group-NEC' = paste(plasma[, 'group'], plasma[, 'hist.nec'], sep = '-'))
plasma$'group-NEC' <- factor(plasma$'group-NEC',
                             levels = c('LAC-Healthy', 'LAC-NEC', 'MIX-Healthy', 'MIX-NEC',
                                        'CSS-Healthy', 'CSS-NEC'))
plot.cmpds <- c('lactate', '2-palmitoylglycerophosphoethanolamine', 
                'acetylcarnitine', 'kynurenate')
# plots of metabolites which differ by NEC
plotMetabolites(plasma, plot.cmpds, 'hist.nec')
```


```
plot.cmpds <- c('2,3-butanediol', '3-indoxyl sulfate', 'hyodeoxycholate')
# plots of metabolites which differ by diet
plotMetabolites(plasma, plot.cmpds, 'group')
```


```
rm(plot.cmpds)
```

## Figure 6 (also). Selected cecal metabolites


```
# add factor to allow plotting by group-NEC
cecal <- cbind(cecal, 'group-NEC' = paste(cecal[, 'group'], cecal[, 'hist.nec'], sep = '-'))
cecal$'group-NEC' <- factor(cecal$'group-NEC',
                            levels = c('LAC-Healthy', 'LAC-NEC', 'MIX-Healthy', 'MIX-NEC',
                                       'CSS-Healthy', 'CSS-NEC'))
plot.cmpds <- c('phenylacetate', 'serotonin (5HT)', 'dihomo-linoleate (20:2n6)',
                'kynurenine')
# plots of metabolites which differ by NEC
plotMetabolites(cecal, plot.cmpds, 'hist.nec')
```


```
plot.cmpds <- c('lactate', 'imidazole propionate', 'palmitoyl ethanolamide',
                'quinaldic acid', '2-myristoylglycerophosphocholine')
plotMetabolites(cecal, plot.cmpds, 'group')
```


```
rm(plot.cmpds)
```

## Figure S2. Cytokine expression


```
pigs <- getMJNPigs(incl.new = F)
exp = read.csv('DI_cytokines.csv', strip.white=T)
exp <- merge(pigs[, c('pigID', 'group', 'hist.nec')], exp)
# x=gene, dodge by groups and/or NEC
exp$group.nec <- paste(exp$group, exp$hist.nec, sep='.')
exp$group.nec <- factor(exp$group.nec,
                        levels = c('LAC.Healthy', 'LAC.NEC','MIX.Healthy', 'MIX.NEC',
                                   'CSS.Healthy', 'CSS.NEC'), ordered = T)
sum_c <- summarySE(exp, measurevar = 'rel.exp', groupvars = c('group.nec', 'hist.nec', 'gene'), na.rm=T)
```


```
NaNs produced
```


```
genes <- levels(sum_c$gene)
for (g in genes) {
  plot <- ggplot(sum_c[sum_c$gene == g, ], aes(x=gene, y=rel.exp, fill = group.nec)) +
    scale_fill_manual(values = group_NEC_colors) + 
    scale_colour_manual(values = group_NEC_colors) +
    geom_errorbar(aes(ymin = rel.exp - se, ymax = rel.exp + se), width = 0.2, 
                  position = position_dodge(width = 0.9)) +
    geom_bar(stat = 'identity', aes(colour = group.nec), position = position_dodge(width = 0.9)) +
    labs(list(title = element_blank(), x = element_blank(), y = 'Relative Expression')) +
    theme(panel.background = element_blank(), panel.grid.major = element_blank(),
          panel.grid.minor = element_blank(), axis.line = element_line(colour='black'),
          axis.title.x = element_text(vjust=-0.5), axis.title.y = element_text(vjust = 1.5),
          text = element_text(face = 'bold', size = 18),
          axis.text = element_text(color = 'black'),
          legend.title = element_blank(), legend.position = 'right') +
    guides(colour = guide_legend(nrow = 1, byrow = T))
  print(plot)
  print(with(exp[exp$gene == g, ], pairwise.wilcox.test(rel.exp, group.nec, p.adj = 'holm')))
}
```


```
    Pairwise comparisons using Wilcoxon rank sum test 

data:  rel.exp and group.nec 

            LAC.Healthy LAC.NEC MIX.Healthy MIX.NEC CSS.Healthy
LAC.NEC     1.00        -       -           -       -          
MIX.Healthy 1.00        1.00    -           -       -          
MIX.NEC     0.32        1.00    0.38        -       -          
CSS.Healthy 0.68        1.00    0.83        1.00    -          
CSS.NEC     0.11        1.00    0.22        1.00    1.00       

P value adjustment method: holm 

    Pairwise comparisons using Wilcoxon rank sum test 

data:  rel.exp and group.nec 

            LAC.Healthy LAC.NEC MIX.Healthy MIX.NEC CSS.Healthy
LAC.NEC     1.00        -       -           -       -          
MIX.Healthy 1.00        1.00    -           -       -          
MIX.NEC     1.00        1.00    1.00        -       -          
CSS.Healthy 1.00        1.00    1.00        1.00    -          
CSS.NEC     0.15        1.00    0.44        1.00    1.00       

P value adjustment method: holm 

    Pairwise comparisons using Wilcoxon rank sum test 

data:  rel.exp and group.nec 

            LAC.Healthy LAC.NEC MIX.Healthy MIX.NEC CSS.Healthy
LAC.NEC     1.000       -       -           -       -          
MIX.Healthy 1.000       1.000   -           -       -          
MIX.NEC     0.044       1.000   0.686       -       -          
CSS.Healthy 0.673       1.000   1.000       1.000   -          
CSS.NEC     0.122       1.000   1.000       1.000   1.000      

P value adjustment method: holm 

    Pairwise comparisons using Wilcoxon rank sum test 

data:  rel.exp and group.nec 

            LAC.Healthy LAC.NEC MIX.Healthy MIX.NEC CSS.Healthy
LAC.NEC     1           -       -           -       -          
MIX.Healthy 1           1       -           -       -          
MIX.NEC     1           1       1           -       -          
CSS.Healthy 1           1       1           1       -          
CSS.NEC     1           1       1           1       1          

P value adjustment method: holm 

    Pairwise comparisons using Wilcoxon rank sum test 

data:  rel.exp and group.nec 

            LAC.Healthy LAC.NEC MIX.Healthy MIX.NEC CSS.Healthy
LAC.NEC     1.00        -       -           -       -          
MIX.Healthy 1.00        1.00    -           -       -          
MIX.NEC     1.00        1.00    1.00        -       -          
CSS.Healthy 1.00        1.00    0.84        0.50    -          
CSS.NEC     1.00        1.00    1.00        1.00    1.00       

P value adjustment method: holm
```


```
rm(pigs, exp, sum_c, g, genes, plot)
```

## Clean up and print out session info


```
rm(list=ls())
sessionInfo()
```


```
R version 3.3.1 (2016-06-21)
Platform: x86_64-w64-mingw32/x64 (64-bit)
Running under: Windows 7 x64 (build 7601) Service Pack 1

locale:
[1] LC_COLLATE=English_United States.1252  LC_CTYPE=English_United States.1252   
[3] LC_MONETARY=English_United States.1252 LC_NUMERIC=C                          
[5] LC_TIME=English_United States.1252    

attached base packages:
[1] stats     graphics  grDevices utils     datasets  methods   base     

other attached packages:
 [1] ggrepel_0.7.0      pheatmap_1.0.8     RColorBrewer_1.1-2 gridExtra_2.3      phyloseq_1.19.1   
 [6] ggthemes_3.4.0     scales_0.5.0       plyr_1.8.4         reshape2_1.4.2     ggplot2_2.2.1     
[11] multcomp_1.4-7     TH.data_1.0-8      MASS_7.3-47        survival_2.41-3    mvtnorm_1.0-6     

loaded via a namespace (and not attached):
 [1] zoo_1.8-0           splines_3.3.1       lattice_0.20-35     rhdf5_2.18.0        colorspace_1.3-2   
 [6] stats4_3.3.1        mgcv_1.8-22         rlang_0.1.2         BiocGenerics_0.20.0 foreach_1.4.3      
[11] stringr_1.2.0       zlibbioc_1.20.0     Biostrings_2.42.1   munsell_0.4.3       gtable_0.2.0       
[16] codetools_0.2-15    labeling_0.3        Biobase_2.34.0      knitr_1.17          permute_0.9-4      
[21] IRanges_2.8.2       biomformat_1.2.0    parallel_3.3.1      Rcpp_0.12.12        vegan_2.4-4        
[26] S4Vectors_0.12.2    jsonlite_1.5        XVector_0.14.1      digest_0.6.12       stringi_1.1.5      
[31] grid_3.3.1          ade4_1.7-8          tools_3.3.1         sandwich_2.4-0      magrittr_1.5       
[36] lazyeval_0.2.0      tibble_1.3.4        cluster_2.0.6       ape_4.1             pkgconfig_2.0.1    
[41] Matrix_1.2-11       data.table_1.10.4   assertthat_0.2.0    iterators_1.0.8     multtest_2.30.0    
[46] igraph_1.1.2        nlme_3.1-131
```

LS0tDQp0aXRsZTogIk1ldGFib2xvbWljIFNpZ25hdHVyZXMgRGlzdGluZ3Vpc2ggdGhlIEltcGFjdCBvZiBGb3JtdWxhIENhcmJvaHlkcmF0ZXMgb24gRGlzZWFzZSBPdXRjb21lIGluIGEgUHJldGVybSBQaWdsZXQgTW9kZWwgb2YgTkVDIg0Kb3V0cHV0OiBodG1sX25vdGVib29rDQotLS0NCiAgDQogIFRoaXMgZG9jdW1lbnQgaXMgYSByZWNvcmQgb2YgYWxsIGFuYWx5c2lzIHN0ZXBzIHJlcXVpcmVkIHRvIHJlcHJvZHVjZSB0aGUgd29yayByZXBvcnRlZCBpbiB0aGUgbWFudXNjcmlwdC4gQWx0aG91Z2ggc29tZSBmb3JtYXR0aW5nIGRpZmZlcmVuY2VzIG1heSBleGlzdCBmcm9tIHRoZSBmaW5hbCBwdWJsaXNoZWQgZmlndXJlcywgdGhlIG1haW4gcmVzdWx0cyBhbmQgY29uY2x1c2lvbnMgYXJlIGVxdWl2YWxlbnQuICBEYXRhIGZpbGVzIHVzZWQgZm9yIHRoaXMgYW5hbHlzaXMgc2hvdWxkIGJlIHBsYWNlZCBpbiB0aGUgc2FtZSBmb2xkZXIgYXMgdGhpcyAuUm1kIGZpbGUuDQoNCmBgYHtyIGdsb2JhbF9vcHRpb25zfQ0Ka25pdHI6Om9wdHNfY2h1bmskc2V0KHdhcm5pbmc9RkFMU0UsIG1lc3NhZ2U9RkFMU0UpDQpgYGANCg0KYGBge3IgcmVzdWx0cz0naGlkZSd9DQoNCmxpYnJhcnkobXVsdGNvbXApDQpsaWJyYXJ5KGdncGxvdDIpDQpsaWJyYXJ5KHJlc2hhcGUyKQ0KbGlicmFyeShwbHlyKQ0KbGlicmFyeShzY2FsZXMpDQpsaWJyYXJ5KGdndGhlbWVzKQ0KbGlicmFyeShwaHlsb3NlcSkNCmxpYnJhcnkoZ3JpZEV4dHJhKQ0KbGlicmFyeShSQ29sb3JCcmV3ZXIpDQpsaWJyYXJ5KHBoZWF0bWFwKSAgDQpsaWJyYXJ5KGdncmVwZWwpDQoNCg0KY29sb3JzPWMoJyM0ZGFmNGEnLCAnIzM3N2ViOCcsICcjZTQxYTFjJykNCmdyb3VwX2NvbG9ycz1jKCcjNGRhZjRhJywgJyMzNzdlYjgnLCAnI2U0MWExYycpDQpkaWV0X2NvbG9ycyA8LSBjKCcjNGRhZjRhJywgJyMzNzdlYjgnLCAnI2U0MWExYycpDQpobl9jb2xvcnMgPC0gYygnYmxhY2snLCAncmVkMicpIA0KZ3JvdXBfTkVDX2NvbG9ycyA8LSBjKCcjNGRhZjRhJywgJyMzMTZmMmYnLCAnIzM3N2ViOCcsICcjMWU0NjY2JywgJyNlNDFhMWMnLCAnI2IwMTQxNCcpICANCg0KIyBzZXQgdGhlbWUNCnRoZW1lX3NldCh0aGVtZV9jbGFzc2ljKCkgKyB0aGVtZSh0ZXh0ID0gZWxlbWVudF90ZXh0KGZhbWlseSA9ICdBcmlhbCcsIGZhY2UgPSAnYm9sZCcsIGNvbG9yID0gMSksDQogICAgICAgICAgICAgICAgICAgICAgICAgICAgICAgICAgYXhpcy50ZXh0ID0gZWxlbWVudF90ZXh0KGNvbG9yID0gMSwgc2l6ZSA9IHJlbCgxLjEpKSkpDQp0aGVtZV91cGRhdGUoYXhpcy50ZXh0ID0gZWxlbWVudF90ZXh0KGNvbG9yID0gJ2JsYWNrJyksDQogICAgICAgICAgICAgc3RyaXAudGV4dCA9IGVsZW1lbnRfdGV4dChjb2xvciA9ICdibGFjaycpLA0KICAgICAgICAgICAgIGF4aXMudGlja3MgPSBlbGVtZW50X2xpbmUoY29sb3IgPSAnYmxhY2snKSkNCndpbmRvd3NGb250cyhBcmlhbD13aW5kb3dzRm9udCgiVFQgQXJpYWwiKSkNCg0KDQptb3JwaC53cml0dGVuID0gRkFMU0UgIA0KDQojIHV0aWxpdHkgZnVuY3Rpb25zIHRvIGJlIHVzZWQgaW4gYW5hbHlzaXMgb2YgcGlnbGV0IE5FQyBjYXJib2h5ZHJhdGUgc3R1ZHkNCmdldE1KTlBpZ3MgPC0gZnVuY3Rpb24oaW5jbC5uZXcgPSBGQUxTRSkgew0KICAjIHJlYWQgaW4gZGF0YQ0KICBwaWdzIDwtIHJlYWQuY3N2KCdwaWdzX2FudGhyb3BvbWV0cmljc19tb3JwaG9tZXRyeV9zZXZlcml0eS5jc3YnLCBzdHJpcC53aGl0ZT1UKQ0KICANCiAgIyBwYXJzZSAncGlnSUQnIGludG8gc2VwYXJhdGUgY29sdW1ucyBmb3IgbGl0dGVyLCBsZXR0ZXINCiAgcGlncyA8LSBjYmluZChmYWN0b3IodW5saXN0KGxhcHBseShwaWdzWywgJ3BpZ0lEJ10sIGZ1bmN0aW9uKHgpe3N1YnN0cih4LCA0LCA0KX0pKSksIHBpZ3MpDQogIHBpZ3MgPC0gY2JpbmQoZmFjdG9yKHVubGlzdChsYXBwbHkocGlnc1ssICdwaWdJRCddLCBmdW5jdGlvbih4KXthcy5udW1lcmljKHN1YnN0cih4LCAxLCAzKSl9KSkpLCBwaWdzKQ0KICBuYW1lcyhwaWdzKVsxOjJdIDwtIGMoJ2xpdHRlcicsICdsZXR0ZXInKQ0KICANCiAgIyBhZGQgaW4gY2FsY3VsYXRlZCBjb2x1bW5zIHRvIHBpZ3MNCiAgcGlncyRjbGluLnRvdCA8LSBwaWdzJGNsaW4uc3QgKyBwaWdzJGNsaW4uamUgKyBwaWdzJGNsaW4uaWwgKyBwaWdzJGNsaW4uY28NCiAgcGlncyRoaXN0LnRvdCA8LSBwaWdzJGhpc3QucGogKyBwaWdzJGhpc3QuZGkgKyBwaWdzJGhpc3QuY28NCiAgcGlncyRjbGluLm1heCA8LSBhcHBseShwaWdzWywgYygnY2xpbi5zdCcsICdjbGluLmplJywgJ2NsaW4uaWwnLCAnY2xpbi5jbycpXSwgMSwgDQogICAgICAgICAgICAgICAgICAgICAgICAgZnVuY3Rpb24oeCl7c3VwcHJlc3NXYXJuaW5ncyhtYXgoeCwgbmEucm09VCkpfSkNCiAgcGlncyRoaXN0Lm1heCA8LSBhcHBseShwaWdzWywgYygnaGlzdC5waicsICdoaXN0LmRpJywgJ2hpc3QuY28nKV0sIDEsDQogICAgICAgICAgICAgICAgICAgICAgICAgZnVuY3Rpb24oeCl7c3VwcHJlc3NXYXJuaW5ncyhtYXgoeCwgbmEucm09VCkpfSkNCiAgcGlncyRjbGluLm5lYyA8LSBwaWdzJGNsaW4ubWF4ID4gMg0KICBwaWdzJGNsaW4ubmVjIDwtIGZhY3RvcihwaWdzJGNsaW4ubmVjLCBsYWJlbHM9YygnSGVhbHRoeScsICdORUMnKSkNCiAgcGlncyRoaXN0Lm5lYyA8LSBwaWdzJGhpc3QubWF4ID4gMQ0KICBwaWdzJGhpc3QubmVjIDwtIGZhY3RvcihwaWdzJGhpc3QubmVjLCBsYWJlbHM9YygnSGVhbHRoeScsICdORUMnKSkNCiAgDQogIHBpZ3MkZG9iIDwtIGFzLkRhdGUoYXMuY2hhcmFjdGVyKHBpZ3MkZG9iKSwgJyVZJW0lZCcpDQogIHBpZ3MkZG9kIDwtIGFzLkRhdGUoYXMuY2hhcmFjdGVyKHBpZ3MkZG9kKSwgJyVZJW0lZCcpDQogIA0KICAjIGNvbnZlcnQgdGltZSBvZiBkZWF0aCAodG9kKSB0byBob3VycyBpbiBkZWNpbWFsIGZvcm0NCiAgcGlncyR0b2QuZCA8LSBzYXBwbHkoc3Ryc3BsaXQoYXMuY2hhcmFjdGVyKHBpZ3NbLCAndG9kJ10pLCAnOicpLCBmdW5jdGlvbih4KXsNCiAgICB4IDwtIGFzLm51bWVyaWMoeCkNCiAgICB4WzFdK3hbMl0vNjANCiAgfSkNCiAgDQogICMgMjQgaHJzIGZvciBlYWNoIGRheSBvZiBmZWVkaW5nIChmaXJzdCAyIGRheXMgb2YgbGlmZSBhcmUgVFBOIG9ubHkpIHBsdXMgdGhlIHRpbWUgb2YgZGVhdGggDQogICMgICBvbiBmaW5hbCBkYXkgbWludXMgMTIgaHJzIGIvYyBmZWVkaW5nIGJlZ2lucyBhdCAxMjowMA0KICBwaWdzJGhycy5hZnRlci5mZWVkIDwtIGFzLm51bWVyaWMoKHBpZ3MkZG9kLXBpZ3MkZG9iLTIpKjI0ICsgcGlncyR0b2QuZCAtIDEyKSANCiAgDQogICMgd2VpZ2h0LmdhaW4gZXhwcmVzc2VkIGFzOiAgZyAvIChrZyAqIGRheSkgIGZyb20gYmlydGh3ZWlnaHQgdG8gZGVhdGh3ZWlnaHQNCiAgcGlncyR3ZWlnaHQuZ2FpbiA8LSAocGlncyR3ZWlnaHQuZCAtIHBpZ3Mkd2VpZ2h0LmIpIC8gDQogICAgKChwaWdzJHdlaWdodC5iIC8gMTAwMCkgKiAocGlncyRocnMuYWZ0ZXIuZmVlZCAvIDI0KSkNCiAgDQogICMgc2F2ZSBuZXcgZmlsZSB3LyBhZGRlZCBjb2x1bW5zIChvbmx5IGZpcnN0IHRpbWUgdGhpcyBmdW5jdGlvbiBpcyBjYWxsZWQpDQogIGlmKCFtb3JwaC53cml0dGVuKSB7DQogICAgd3JpdGUuY3N2KHBpZ3MsICdwaWdzX2FudGhyb3BvbWV0cmljc19tb3JwaG9tZXRyeV9zZXZlcml0eV9wcm9jZXNzZWQuY3N2Jywgcm93Lm5hbWVzID0gRikNCiAgICBtb3JwaC53cml0dGVuID0gVFJVRQ0KICB9DQogIA0KICAjIGFjY29yZGluZyB0byBwcmVmZXJlbmNlIHBhc3NlZCBpbiBmdW5jdGlvbiBjYWxsLCBpbmNsdWRlIG9yIHJlbW92ZSBhbGwgbmV3Ym9ybg0KICBpZihpbmNsLm5ldykgew0KICAgICMgZm9yY2UgdXNlIG9mIHRoaXMgb3JkZXIgTCAtPiBSDQogICAgcGlncyRncm91cCA8LSBmYWN0b3IocGlncyRncm91cCwgbGV2ZWxzPWMoJ05FVycsICdMQUMnLCAnTUlYJywgJ0NTUycpLCBvcmRlcmVkPVQpCQ0KICB9DQogIGVsc2Ugew0KICAgIHBpZ3MgPC0gcGlnc1t3aGljaChwaWdzWywgJ2dyb3VwJ10gIT0gJ05FVycpLCBdICANCiAgICBwaWdzJGdyb3VwIDwtIGRyb3BsZXZlbHMocGlncyRncm91cCkNCiAgICAjIGZvcmNlIHVzZSBvZiB0aGlzIG9yZGVyIEwgLT4gUg0KICAgIHBpZ3MkZ3JvdXAgPC0gZmFjdG9yKHBpZ3MkZ3JvdXAsIGxldmVscz1jKCdMQUMnLCAnTUlYJywgJ0NTUycpLCBvcmRlcmVkPVQpICANCiAgfQ0KICANCiAgIyByZW1vdmUgcGlncyBmcm9tIGxpdHRlcnMgMjI4IChubyBhbXBsaWZpY2F0aW9uKSwgMjM0IChlYXJseSBkZWF0aHMpLCANCiAgIyAgIGFuZCAyNDgtMjQ5IChmcmlkZ2UgZGllZCAtIHVzZWQgZGlmZmVyZW50IGZvcm11bGEpDQogIHBpZ3MgPC0gcGlnc1shYyhwaWdzJGxpdHRlciAlaW4lIGMoMjI4LCAyMzQsIDI0OCwgMjQ5KSksIF0NCiAgDQogICMgcmVtb3ZlIHBpZ3MgZm91bmQgd2l0aCBwZXJmb3JhdGVkIHN0b21hY2hzIGZyb20gb3JvZ2FzdHJpYyB0dWJlDQogIHBpZ3MgPC0gcGlnc1shYyhwaWdzJHBpZ0lEICVpbiUgYygnMjE1RScsJzIxNUknLCcyMTVMJykpLCBdDQogIA0KICAjIHJlbW92ZSBwaWdzIHdoaWNoIHJlY2VpdmVkIGxhY3Rvc2Ugb3IgbWl4IGRpZXQgZnJvbSBsaXR0ZXJzIDIxMiwgMjEzIA0KICAjICAgKGJhZCBiYXRjaCBvZiBsYWN0b3NlL21peCBmb3JtdWxhcykNCiAgcGlncyA8LSBwaWdzWyFjKHBpZ3MkbGl0dGVyICVpbiUgYygyMTIsIDIxMykgJiBwaWdzJGdyb3VwICVpbiUgYygnTEFDJywgJ01JWCcpKSwgXQ0KICBwaWdzJGxpdHRlciA8LSBkcm9wbGV2ZWxzKHBpZ3MkbGl0dGVyKQ0KICANCiAgcmV0dXJuKHBpZ3MpDQp9DQoNCnNlIDwtIGZ1bmN0aW9uKHgpIHsNCiAgc2QoeCkgLyBzcXJ0KGxlbmd0aCh4KSkNCn0NCg0KIyMgR2l2ZXMgY291bnQsIG1lYW4sIHN0YW5kYXJkIGRldmlhdGlvbiwgc3RhbmRhcmQgZXJyb3Igb2YgdGhlIG1lYW4sIGFuZCANCiMjICAgIGNvbmZpZGVuY2UgaW50ZXJ2YWwgKGRlZmF1bHQgOTUlKS4NCiMjICAgZGF0YTogYSBkYXRhIGZyYW1lLg0KIyMgICBtZWFzdXJldmFyOiB0aGUgbmFtZSBvZiBhIGNvbHVtbiB0aGF0IGNvbnRhaW5zIHRoZSB2YXJpYWJsZSB0byBiZSBzdW1tYXJpZXplZA0KIyMgICBncm91cHZhcnM6IGEgdmVjdG9yIGNvbnRhaW5pbmcgbmFtZXMgb2YgY29sdW1ucyB0aGF0IGNvbnRhaW4gZ3JvdXBpbmcgdmFyaWFibGVzDQojIyAgIG5hLnJtOiBhIGJvb2xlYW4gdGhhdCBpbmRpY2F0ZXMgd2hldGhlciB0byBpZ25vcmUgTkEncw0KIyMgICBjb25mLmludGVydmFsOiB0aGUgcGVyY2VudCByYW5nZSBvZiB0aGUgY29uZmlkZW5jZSBpbnRlcnZhbCAoZGVmYXVsdCBpcyA5NSUpDQojIyMjIyAgZnJvbSBodHRwOi8vd3d3LmNvb2tib29rLXIuY29tL0dyYXBocy9QbG90dGluZ19tZWFuc19hbmRfZXJyb3JfYmFyc18oZ2dwbG90MikNCnN1bW1hcnlTRSA8LSBmdW5jdGlvbihkYXRhPU5VTEwsIG1lYXN1cmV2YXIsIGdyb3VwdmFycz1OVUxMLCBuYS5ybT1GQUxTRSwNCiAgICAgICAgICAgICAgICAgICAgICBjb25mLmludGVydmFsPS45NSwgLmRyb3A9VFJVRSkgew0KICANCiAgIyBOZXcgdmVyc2lvbiBvZiBsZW5ndGggd2hpY2ggY2FuIGhhbmRsZSBOQSdzOiBpZiBuYS5ybT09VCwgZG9uJ3QgY291bnQgdGhlbQ0KICBsZW5ndGgyIDwtIGZ1bmN0aW9uICh4LCBuYS5ybT1GQUxTRSkgew0KICAgIGlmIChuYS5ybSkgc3VtKCFpcy5uYSh4KSkNCiAgICBlbHNlICAgICAgIGxlbmd0aCh4KQ0KICB9DQogIA0KICAjIFRoaXMgZG9lcyB0aGUgc3VtbWFyeS4gRm9yIGVhY2ggZ3JvdXAncyBkYXRhIGZyYW1lLCByZXR1cm4gYSB2ZWN0b3Igd2l0aA0KICAjIE4sIG1lYW4sIGFuZCBzZA0KICBkYXRhYyA8LSBkZHBseShkYXRhLCBncm91cHZhcnMsIC5kcm9wPS5kcm9wLA0KICAgICAgICAgICAgICAgICAuZnVuID0gZnVuY3Rpb24oeHgsIGNvbCkgew0KICAgICAgICAgICAgICAgICAgIGMoTiAgICA9IGxlbmd0aDIoeHhbW2NvbF1dLCBuYS5ybT1uYS5ybSksDQogICAgICAgICAgICAgICAgICAgICBtZWFuID0gbWVhbiAgICh4eFtbY29sXV0sIG5hLnJtPW5hLnJtKSwNCiAgICAgICAgICAgICAgICAgICAgIHNkICAgPSBzZCAgICAgKHh4W1tjb2xdXSwgbmEucm09bmEucm0pDQogICAgICAgICAgICAgICAgICAgKQ0KICAgICAgICAgICAgICAgICB9LA0KICAgICAgICAgICAgICAgICBtZWFzdXJldmFyDQogICkNCiAgDQogICMgUmVuYW1lIHRoZSAibWVhbiIgY29sdW1uICAgIA0KICBkYXRhYyA8LSByZW5hbWUoZGF0YWMsIGMoIm1lYW4iID0gbWVhc3VyZXZhcikpDQogIA0KICBkYXRhYyRzZSA8LSBkYXRhYyRzZCAvIHNxcnQoZGF0YWMkTikgICMgQ2FsY3VsYXRlIHN0YW5kYXJkIGVycm9yIG9mIHRoZSBtZWFuDQogIA0KICAjIENvbmZpZGVuY2UgaW50ZXJ2YWwgbXVsdGlwbGllciBmb3Igc3RhbmRhcmQgZXJyb3INCiAgIyBDYWxjdWxhdGUgdC1zdGF0aXN0aWMgZm9yIGNvbmZpZGVuY2UgaW50ZXJ2YWw6IA0KICAjIGUuZy4sIGlmIGNvbmYuaW50ZXJ2YWwgaXMgLjk1LCB1c2UgLjk3NSAoYWJvdmUvYmVsb3cpLCBhbmQgdXNlIGRmPU4tMQ0KICBjaU11bHQgPC0gcXQoY29uZi5pbnRlcnZhbC8yICsgLjUsIGRhdGFjJE4tMSkNCiAgZGF0YWMkY2kgPC0gZGF0YWMkc2UgKiBjaU11bHQNCiAgDQogIHJldHVybihkYXRhYykNCn0NCg0KYnJpZ2h0bmVzcyA8LSBmdW5jdGlvbihyZ2Jjb2wsIHYpIHsNCiAgY29udiA8LSBhcy5saXN0KGFzLmRhdGEuZnJhbWUodChyZ2IyaHN2KGNvbDJyZ2IocmdiY29sKSkpKSkNCiAgY29udltbM11dIDwtIHYNCiAgZG8uY2FsbChoc3YsIGNvbnYpDQp9DQoNCmBgYA0KDQojIyBUYWJsZSAyLiBCb2R5IHdlaWdodHMgYW5kIGd1dCBtb3JwaG9tZXRyeQ0KDQpgYGB7cn0NCnBpZ3MgPC0gZ2V0TUpOUGlncyhpbmNsLm5ldyA9IEYpDQoNCiMgZGVzY3JpcHRpdmUgc3VtbWFyaWVzDQp3aXRoKHBpZ3MsIHRhYmxlKGdyb3VwLCBoaXN0Lm5lYykpDQp3aXRoKHBpZ3MsIGFnZ3JlZ2F0ZShocnMuYWZ0ZXIuZmVlZCB+IGdyb3VwICsgaGlzdC5uZWMsIEZVTj1tZWFuKSkNCndpdGgocGlncywgYWdncmVnYXRlKGhycy5hZnRlci5mZWVkIH4gZ3JvdXAgKyBoaXN0Lm5lYywgRlVOPXNlKSkNCndpdGgocGlncywgYWdncmVnYXRlKHdlaWdodC5iIH4gZ3JvdXAgKyBoaXN0Lm5lYywgRlVOPW1lYW4pKQ0Kd2l0aChwaWdzLCBhZ2dyZWdhdGUod2VpZ2h0LmIgfiBncm91cCArIGhpc3QubmVjLCBGVU49c2UpKQ0Kd2l0aChwaWdzLCBhZ2dyZWdhdGUod2VpZ2h0LmQgfiBncm91cCArIGhpc3QubmVjLCBGVU49bWVhbikpDQp3aXRoKHBpZ3MsIGFnZ3JlZ2F0ZSh3ZWlnaHQuZCB+IGdyb3VwICsgaGlzdC5uZWMsIEZVTj1zZSkpDQp3aXRoKHBpZ3MsIGFnZ3JlZ2F0ZSh3ZWlnaHQuZ2FpbiB+IGdyb3VwICsgaGlzdC5uZWMsIEZVTj1tZWFuKSkNCndpdGgocGlncywgYWdncmVnYXRlKHdlaWdodC5nYWluIH4gZ3JvdXAgKyBoaXN0Lm5lYywgRlVOPXNlKSkNCndpdGgocGlncywgYWdncmVnYXRlKHBqLnZoIH4gZ3JvdXAgKyBoaXN0Lm5lYywgRlVOPW1lYW4pKQ0Kd2l0aChwaWdzLCBhZ2dyZWdhdGUocGoudmggfiBncm91cCArIGhpc3QubmVjLCBGVU49c2UpKQ0Kd2l0aChwaWdzLCBhZ2dyZWdhdGUocGouY2QgfiBncm91cCArIGhpc3QubmVjLCBGVU49bWVhbikpDQp3aXRoKHBpZ3MsIGFnZ3JlZ2F0ZShwai5jZCB+IGdyb3VwICsgaGlzdC5uZWMsIEZVTj1zZSkpDQp3aXRoKHBpZ3MsIGFnZ3JlZ2F0ZShkaS52aCB+IGdyb3VwICsgaGlzdC5uZWMsIEZVTj1tZWFuKSkNCndpdGgocGlncywgYWdncmVnYXRlKGRpLnZoIH4gZ3JvdXAgKyBoaXN0Lm5lYywgRlVOPXNlKSkNCndpdGgocGlncywgYWdncmVnYXRlKGRpLmNkIH4gZ3JvdXAgKyBoaXN0Lm5lYywgRlVOPW1lYW4pKQ0Kd2l0aChwaWdzLCBhZ2dyZWdhdGUoZGkuY2QgfiBncm91cCArIGhpc3QubmVjLCBGVU49c2UpKQ0Kd2l0aChwaWdzLCBhZ2dyZWdhdGUoY28uY2QgfiBncm91cCArIGhpc3QubmVjLCBGVU49bWVhbikpDQp3aXRoKHBpZ3MsIGFnZ3JlZ2F0ZShjby5jZCB+IGdyb3VwICsgaGlzdC5uZWMsIEZVTj1zZSkpDQojIHRlc3QgZm9yIGRpZmZlcmVuY2VzIGJldHdlZW4gaGVhbHRoeSBncm91cHMNCnBpZ3MuaCA8LSBwaWdzW3doaWNoKHBpZ3NbLCAnaGlzdC5uZWMnXSA9PSAnSGVhbHRoeScpLCBdDQpzdW1tYXJ5KGdsaHQoZ2xtKGhycy5hZnRlci5mZWVkIH4gZ3JvdXAsIGZhbWlseT1nYXVzc2lhbiwgZGF0YSA9IHBpZ3MuaCksIGxpbmZjdCA9IG1jcChncm91cD0nVHVrZXknKSkpDQpzdW1tYXJ5KGdsaHQoZ2xtKHdlaWdodC5iIH4gZ3JvdXAsIGZhbWlseT1nYXVzc2lhbiwgZGF0YT1waWdzLmgpLCBsaW5mY3QgPSBtY3AoZ3JvdXA9J1R1a2V5JykpKQ0Kc3VtbWFyeShnbGh0KGdsbSh3ZWlnaHQuZCB+IGdyb3VwLCBmYW1pbHk9Z2F1c3NpYW4sIGRhdGE9cGlncy5oKSwgbGluZmN0ID0gbWNwKGdyb3VwPSdUdWtleScpKSkNCnN1bW1hcnkoZ2xodChnbG0od2VpZ2h0LmdhaW4gfiBncm91cCwgZmFtaWx5PWdhdXNzaWFuLCBkYXRhPXBpZ3MuaCksIGxpbmZjdCA9IG1jcChncm91cD0nVHVrZXknKSkpDQpzdW1tYXJ5KGdsaHQoZ2xtKHBqLnZoIH4gZ3JvdXAsIGZhbWlseT1nYXVzc2lhbiwgZGF0YT1waWdzLmgpLCBsaW5mY3QgPSBtY3AoZ3JvdXA9J1R1a2V5JykpKQ0Kc3VtbWFyeShnbGh0KGdsbShwai5jZCB+IGdyb3VwLCBmYW1pbHk9Z2F1c3NpYW4sIGRhdGE9cGlncy5oKSwgbGluZmN0ID0gbWNwKGdyb3VwPSdUdWtleScpKSkNCnN1bW1hcnkoZ2xodChnbG0oZGkudmggfiBncm91cCwgZmFtaWx5PWdhdXNzaWFuLCBkYXRhPXBpZ3MuaCksIGxpbmZjdCA9IG1jcChncm91cD0nVHVrZXknKSkpDQpzdW1tYXJ5KGdsaHQoZ2xtKGRpLmNkIH4gZ3JvdXAsIGZhbWlseT1nYXVzc2lhbiwgZGF0YT1waWdzLmgpLCBsaW5mY3QgPSBtY3AoZ3JvdXA9J1R1a2V5JykpKQ0Kc3VtbWFyeShnbGh0KGdsbShjby5jZCB+IGdyb3VwLCBmYW1pbHk9Z2F1c3NpYW4sIGRhdGE9cGlncy5oKSwgbGluZmN0ID0gbWNwKGdyb3VwPSdUdWtleScpKSkNCiMgdGVzdCBmb3IgZGlmZmVyZW5jZXMgYmV0d2VlbiBORUMgZ3JvdXBzDQpwaWdzLm4gPC0gcGlnc1t3aGljaChwaWdzWywgJ2hpc3QubmVjJ10gPT0gJ05FQycpLCBdDQpzdW1tYXJ5KGdsaHQoZ2xtKGhycy5hZnRlci5mZWVkIH4gZ3JvdXAsIGZhbWlseT1nYXVzc2lhbiwgZGF0YSA9IHBpZ3MubiksIGxpbmZjdCA9IG1jcChncm91cD0nVHVrZXknKSkpDQpzdW1tYXJ5KGdsaHQoZ2xtKHdlaWdodC5iIH4gZ3JvdXAsIGZhbWlseT1nYXVzc2lhbiwgZGF0YT1waWdzLm4pLCBsaW5mY3QgPSBtY3AoZ3JvdXA9J1R1a2V5JykpKQ0Kc3VtbWFyeShnbGh0KGdsbSh3ZWlnaHQuZCB+IGdyb3VwLCBmYW1pbHk9Z2F1c3NpYW4sIGRhdGE9cGlncy5uKSwgbGluZmN0ID0gbWNwKGdyb3VwPSdUdWtleScpKSkNCnN1bW1hcnkoZ2xodChnbG0od2VpZ2h0LmdhaW4gfiBncm91cCwgZmFtaWx5PWdhdXNzaWFuLCBkYXRhPXBpZ3MubiksIGxpbmZjdCA9IG1jcChncm91cD0nVHVrZXknKSkpDQpzdW1tYXJ5KGdsaHQoZ2xtKHBqLnZoIH4gZ3JvdXAsIGZhbWlseT1nYXVzc2lhbiwgZGF0YT1waWdzLm4pLCBsaW5mY3QgPSBtY3AoZ3JvdXA9J1R1a2V5JykpKQ0Kc3VtbWFyeShnbGh0KGdsbShwai5jZCB+IGdyb3VwLCBmYW1pbHk9Z2F1c3NpYW4sIGRhdGE9cGlncy5uKSwgbGluZmN0ID0gbWNwKGdyb3VwPSdUdWtleScpKSkNCnN1bW1hcnkoZ2xodChnbG0oZGkudmggfiBncm91cCwgZmFtaWx5PWdhdXNzaWFuLCBkYXRhPXBpZ3MubiksIGxpbmZjdCA9IG1jcChncm91cD0nVHVrZXknKSkpDQpzdW1tYXJ5KGdsaHQoZ2xtKGRpLmNkIH4gZ3JvdXAsIGZhbWlseT1nYXVzc2lhbiwgZGF0YT1waWdzLm4pLCBsaW5mY3QgPSBtY3AoZ3JvdXA9J1R1a2V5JykpKQ0Kc3VtbWFyeShnbGh0KGdsbShjby5jZCB+IGdyb3VwLCBmYW1pbHk9Z2F1c3NpYW4sIGRhdGE9cGlncy5uKSwgbGluZmN0ID0gbWNwKGdyb3VwPSdUdWtleScpKSkNCiMgdGVzdCBmb3IgZGlmZmVyZW5jZXMgYmV0d2VlbiBoZWFsdGh5IHZzIE5FQyB3aXRoaW4gZ3JvdXBzDQp3aXRoKHBpZ3Nbd2hpY2gocGlncyRncm91cCA9PSAnTEFDJyksIF0sIHQudGVzdChocnMuYWZ0ZXIuZmVlZCB+IGhpc3QubmVjKSkNCndpdGgocGlnc1t3aGljaChwaWdzJGdyb3VwID09ICdMQUMnKSwgXSwgdC50ZXN0KHdlaWdodC5iIH4gaGlzdC5uZWMpKQ0Kd2l0aChwaWdzW3doaWNoKHBpZ3MkZ3JvdXAgPT0gJ0xBQycpLCBdLCB0LnRlc3Qod2VpZ2h0LmQgfiBoaXN0Lm5lYykpDQp3aXRoKHBpZ3Nbd2hpY2gocGlncyRncm91cCA9PSAnTEFDJyksIF0sIHQudGVzdCh3ZWlnaHQuZ2FpbiB+IGhpc3QubmVjKSkNCndpdGgocGlnc1t3aGljaChwaWdzJGdyb3VwID09ICdMQUMnKSwgXSwgdC50ZXN0KHBqLnZoIH4gaGlzdC5uZWMpKQ0Kd2l0aChwaWdzW3doaWNoKHBpZ3MkZ3JvdXAgPT0gJ0xBQycpLCBdLCB0LnRlc3QocGouY2QgfiBoaXN0Lm5lYykpDQp3aXRoKHBpZ3Nbd2hpY2gocGlncyRncm91cCA9PSAnTEFDJyksIF0sIHQudGVzdChkaS52aCB+IGhpc3QubmVjKSkNCndpdGgocGlnc1t3aGljaChwaWdzJGdyb3VwID09ICdMQUMnKSwgXSwgdC50ZXN0KGRpLmNkIH4gaGlzdC5uZWMpKQ0Kd2l0aChwaWdzW3doaWNoKHBpZ3MkZ3JvdXAgPT0gJ0xBQycpLCBdLCB0LnRlc3QoY28uY2QgfiBoaXN0Lm5lYykpDQp3aXRoKHBpZ3Nbd2hpY2gocGlncyRncm91cCA9PSAnTUlYJyksIF0sIHQudGVzdChocnMuYWZ0ZXIuZmVlZCB+IGhpc3QubmVjKSkNCndpdGgocGlnc1t3aGljaChwaWdzJGdyb3VwID09ICdNSVgnKSwgXSwgdC50ZXN0KHdlaWdodC5iIH4gaGlzdC5uZWMpKQ0Kd2l0aChwaWdzW3doaWNoKHBpZ3MkZ3JvdXAgPT0gJ01JWCcpLCBdLCB0LnRlc3Qod2VpZ2h0LmQgfiBoaXN0Lm5lYykpDQp3aXRoKHBpZ3Nbd2hpY2gocGlncyRncm91cCA9PSAnTUlYJyksIF0sIHQudGVzdCh3ZWlnaHQuZ2FpbiB+IGhpc3QubmVjKSkNCndpdGgocGlnc1t3aGljaChwaWdzJGdyb3VwID09ICdNSVgnKSwgXSwgdC50ZXN0KHBqLnZoIH4gaGlzdC5uZWMpKQ0Kd2l0aChwaWdzW3doaWNoKHBpZ3MkZ3JvdXAgPT0gJ01JWCcpLCBdLCB0LnRlc3QocGouY2QgfiBoaXN0Lm5lYykpDQp3aXRoKHBpZ3Nbd2hpY2gocGlncyRncm91cCA9PSAnTUlYJyksIF0sIHQudGVzdChkaS52aCB+IGhpc3QubmVjKSkNCndpdGgocGlnc1t3aGljaChwaWdzJGdyb3VwID09ICdNSVgnKSwgXSwgdC50ZXN0KGRpLmNkIH4gaGlzdC5uZWMpKQ0Kd2l0aChwaWdzW3doaWNoKHBpZ3MkZ3JvdXAgPT0gJ01JWCcpLCBdLCB0LnRlc3QoY28uY2QgfiBoaXN0Lm5lYykpDQp3aXRoKHBpZ3Nbd2hpY2gocGlncyRncm91cCA9PSAnQ1NTJyksIF0sIHQudGVzdChocnMuYWZ0ZXIuZmVlZCB+IGhpc3QubmVjKSkNCndpdGgocGlnc1t3aGljaChwaWdzJGdyb3VwID09ICdDU1MnKSwgXSwgdC50ZXN0KHdlaWdodC5iIH4gaGlzdC5uZWMpKQ0Kd2l0aChwaWdzW3doaWNoKHBpZ3MkZ3JvdXAgPT0gJ0NTUycpLCBdLCB0LnRlc3Qod2VpZ2h0LmQgfiBoaXN0Lm5lYykpDQp3aXRoKHBpZ3Nbd2hpY2gocGlncyRncm91cCA9PSAnQ1NTJyksIF0sIHQudGVzdCh3ZWlnaHQuZ2FpbiB+IGhpc3QubmVjKSkNCndpdGgocGlnc1t3aGljaChwaWdzJGdyb3VwID09ICdDU1MnKSwgXSwgdC50ZXN0KHBqLnZoIH4gaGlzdC5uZWMpKQ0Kd2l0aChwaWdzW3doaWNoKHBpZ3MkZ3JvdXAgPT0gJ0NTUycpLCBdLCB0LnRlc3QocGouY2QgfiBoaXN0Lm5lYykpDQp3aXRoKHBpZ3Nbd2hpY2gocGlncyRncm91cCA9PSAnQ1NTJyksIF0sIHQudGVzdChkaS52aCB+IGhpc3QubmVjKSkNCndpdGgocGlnc1t3aGljaChwaWdzJGdyb3VwID09ICdDU1MnKSwgXSwgdC50ZXN0KGRpLmNkIH4gaGlzdC5uZWMpKQ0Kd2l0aChwaWdzW3doaWNoKHBpZ3MkZ3JvdXAgPT0gJ0NTUycpLCBdLCB0LnRlc3QoY28uY2QgfiBoaXN0Lm5lYykpDQoNCnJtKHBpZ3MuaCwgcGlncy5uLCBwaWdzKQ0KDQpgYGANCg0KIyMgRmlndXJlIDEuIFBoZW5vdHlwaWMgb3V0Y29tZXMNCg0KYGBge3J9DQpwaWdzIDwtIGdldE1KTlBpZ3MoaW5jbC5uZXcgPSBGKQ0KDQojIGluY2lkZW5jZSBwbG90DQp0PC13aXRoKHBpZ3MsdGFibGUoZ3JvdXAsIGhpc3QubmVjKSkNCnBpZ3MkaGlzdC5uZWMubG9naWNhbCA8LSBwaWdzJGhpc3QubmVjID09ICdORUMnDQpjbGluX25lY19pbmNpZGVuY2Vfc3VtbWFyeSA8LSBzdW1tYXJ5U0UocGlncywgbWVhc3VyZXZhciA9ICdoaXN0Lm5lYy5sb2dpY2FsJywgZ3JvdXB2YXJzID0gJ2dyb3VwJykNCmdncGxvdChjbGluX25lY19pbmNpZGVuY2Vfc3VtbWFyeSwgYWVzKHggPSBncm91cCwgeSA9IGhpc3QubmVjLmxvZ2ljYWwsIGZpbGwgPSBncm91cCkpICsgDQogIHNjYWxlX2ZpbGxfbWFudWFsKHZhbHVlcyA9IGNvbG9ycykgKyBnZW9tX2JhcihzdGF0ID0gJ2lkZW50aXR5Jywgd2lkdGggPSAwLjgpICsNCiAgYW5ub3RhdGUoJ3RleHQnLCB4ID0gMTozLCB5ID0gMC4wNSwgbGFiZWwgPSBwYXN0ZSh0WywyXSwgY2xpbl9uZWNfaW5jaWRlbmNlX3N1bW1hcnkkTiwgc2VwID0gJy8nKSwNCiAgICAgICAgICAgZm9udGZhY2UgPSAnYm9sZCcsIGNvbG9yID0gJ3doaXRlJywgY2V4ID0gNikgKw0KICBsYWJzKGxpc3QodGl0bGUgPSAnTkVDIEluY2lkZW5jZScsIHggPSAnJywgeSA9ICcnKSkgKyBzY2FsZV95X2NvbnRpbnVvdXMoZWxlbWVudF9ibGFuaygpKSArDQogIHRoZW1lKHBhbmVsLmdyaWQubWFqb3IgPSBlbGVtZW50X2JsYW5rKCksIHBhbmVsLmdyaWQubWlub3IgPSBlbGVtZW50X2JsYW5rKCksDQogICAgICAgIGF4aXMubGluZSA9IGVsZW1lbnRfbGluZShjb2xvdXIgPSAnYmxhY2snKSwgYXhpcy50aXRsZS54ID0gZWxlbWVudF90ZXh0KHZqdXN0ID0gLTAuNSksDQogICAgICAgIGF4aXMudGl0bGUueSA9IGVsZW1lbnRfdGV4dCh2anVzdCA9IDEuNSksIHRleHQgPSBlbGVtZW50X3RleHQoZmFjZSA9ICdib2xkJywgc2l6ZSA9IDE4KSwNCiAgICAgICAgYXhpcy50ZXh0ID0gZWxlbWVudF90ZXh0KGNvbG9yID0gJ2JsYWNrJyksIGxlZ2VuZC50aXRsZSA9IGVsZW1lbnRfYmxhbmsoKSwNCiAgICAgICAgbGVnZW5kLnBvc2l0aW9uID0gJ25vbmUnKQ0Kcm0odCwgY2xpbl9uZWNfaW5jaWRlbmNlX3N1bW1hcnkpDQoNCiMgaW5jaWRlbmNlIHN0YXRzDQphZGRtYXJnaW5zKHRhYmxlKHBpZ3MkZ3JvdXAsIHBpZ3MkaGlzdC5uZWMsIHVzZU5BPSdpZmFueScpKQ0KZmlzaGVyLnRlc3QocGlncyRncm91cCwgcGlncyRoaXN0Lm5lYykNCg0KIyBzdXJ2aXZhbCBjdXJ2ZSBwbG90DQojIGNvdW50IGFueSBORUMgcGlnIHNhY3JpZmljZWQgYmVmb3JlIDlhbSAoYmVnaW5uaW5nIG9mIGNvbGxlY3Rpb24gZGF5KQ0KIyAgIChha2EgMTE3ID0gMyBocnMgbGVzcyB0aGFuIDEyMCkgYXMgYW4gZXZlbnQNCnN1cnZfbW9kZWwgPC0gd2l0aChwaWdzLCBTdXJ2KGhycy5hZnRlci5mZWVkLCBhcy5udW1lcmljKGhycy5hZnRlci5mZWVkIDwgMTE5ICYgaGlzdC5uZWMgPT0gJ05FQycpKSkNCg0Kc2YgIDwtICBzdXJ2Zml0KHN1cnZfbW9kZWwgfiBncm91cCwgY29uZi50eXBlPSJsb2ciLCANCiAgICAgICAgICAgICAgICBjb25mLmludD0wLjk1LCB0eXBlPSJrYXBsYW4tbWVpZXIiLCBlcnJvcj0iZ3JlZW53b29kIiwgZGF0YT1waWdzKQ0KDQpvcCA8LSBwYXIoZm9udD0yLCBjZXguYXhpcz0xLjUsIGNleC5sYWI9MS44LCBtYXIgPSBjKDUsIDcsIDQsIDIpICsgMC4xKQ0KcGxvdChzZiwgbHR5PTEsIGx3ZD00LCBmb250PTIsIGZvbnQubGFiPTIsIG1hcmsudGltZT1UUlVFLCB4bGFiPSdIb3VycyBvZiBlbnRlcmFsIGZlZWRpbmcnLA0KICAgICBmcmFtZS5wbG90PUYsIHhheHQ9J24nLCB5YXh0PSduJywgY29sPWNvbG9ycykNCnJlY3QoMCwgMCwgMTI0LCAxLCBjb2wgPSAnd2hpdGUnLCBsdHk9MCkNCmF4aXMoMSwgbHdkPTIsIGZvbnQ9MiwgZm9udC5sYWI9MiwgYXQ9YygwLCAxNDApLCBsd2QudGlja3M9MCwgcG9zPWMoMCwgMCkpDQpheGlzKDEsIGF0PXNlcSgwLCAxMzAsIGJ5PTI0KSwgbHdkPTAsIGx3ZC50aWNrcz0xLCBmb250PTIsIHBvcz1jKDAsIDApKQ0KYXhpcygyLCBsd2Q9MiwgbGFzPTIsIGZvbnQ9MiwgZm9udC5sYWI9MiwgYXQ9c2VxKDAsIDEsIDAuMiksIGxhYmVscz1wYXN0ZShzZXEoMCwgMTAwLCAyMCksICIlIiwgc2VwPSIiKSkNCmxpbmVzKHNmLCBsdHk9MSwgbHdkPTQsIG1hcmsudGltZT1GLCBjb2w9Y29sb3JzKQ0KdGl0bGUoeWxhYj0nTm9uLU5FQyBTdXJ2aXZhbCcsIGZvbnQubGFiPTIsIGNleC5sYWI9MS44LCBsaW5lPTQuNSkNCmxlZ2VuZCh0ZXh0LndpZHRoPTE0LCB4PTIyLCB5PTAuMzUsIGxlZ2VuZD1jKCdMQUMnLCAnTUlYJywgJ0NTUycpLCBjb2w9Y29sb3JzLCBsdHk9MSwgbHdkPTQsDQogICAgICAgYnR5PSJ5IiwgYmc9J3doaXRlJykNCnBhcihvcCkNCg0KIyBzdXJ2aXZhbCBjdXJ2ZSBzdGF0cyAoTG9nLXJhbmsgdGVzdCkNCnN1cnZkaWZmKHN1cnZfbW9kZWx+Z3JvdXAsIGRhdGE9cGlncywgcmhvPTApICAjIyBvdmVyYWxsDQpzdXJ2ZGlmZihzdXJ2X21vZGVsfmdyb3VwLCBkYXRhPXBpZ3MsIHN1YnNldD1ncm91cCAlaW4lIGMoJ0xBQycsICdDU1MnKSkgICMjIHBhaXJ3aXNlDQpzdXJ2ZGlmZihzdXJ2X21vZGVsfmdyb3VwLCBkYXRhPXBpZ3MsIHN1YnNldD1ncm91cCVpbiVjKCdMQUMnLCAnTUlYJykpICAjIyBwYWlyd2lzZQ0Kc3VydmRpZmYoc3Vydl9tb2RlbH5ncm91cCwgZGF0YT1waWdzLCBzdWJzZXQ9Z3JvdXAlaW4lYygnQ1NTJywgJ01JWCcpKSAgIyMgcGFpcndpc2UNCnJtKHN1cnZfbW9kZWwsIHNmLCBvcCkNCg0KIyBzZXZlcml0eSBwbG90cw0KaWR2YXJzIDwtIGMoJ3BpZ0lEJywgJ2xpdHRlcicsICdmYXJtJywgJ2dyb3VwJywgJ3NleCcsICdoaXN0Lm5lYycpDQpnaXZhcnMgPC0gYygnY2xpbi5zdCcsJ2NsaW4uamUnLCdjbGluLmlsJywnY2xpbi5jbycpDQptcGlncyA8LSBtZWx0KHBpZ3NbLCBjKGlkdmFycywgZ2l2YXJzKV0sIGlkLnZhcnM9aWR2YXJzLCB2YXJpYWJsZS5uYW1lPSdzaXRlJykNCnRtcCA8LSBzdW1tYXJ5U0UobXBpZ3MsIG1lYXN1cmV2YXIgPSAndmFsdWUnLCBncm91cHZhcnMgPSBjKCdncm91cCcsICdzaXRlJyksIG5hLnJtPVRSVUUpDQp3aXRoKHRtcCwgZ2dwbG90KGRhdGE9dG1wLCBhZXMoeD1zaXRlLCBmaWxsPWdyb3VwLCB5PXZhbHVlKSkgKyANCiAgICAgICBnZW9tX2Vycm9yYmFyKGFlcyh5bWluID0gdmFsdWUgLSBzZSwgeW1heCA9IHZhbHVlICsgc2UpLCB3aWR0aCA9IDAuMiwgc2l6ZSA9IDEuNSwNCiAgICAgICAgICAgICAgICAgICAgIHBvc2l0aW9uID0gcG9zaXRpb25fZG9kZ2UoMC45KSkgKyANCiAgICAgICBnZW9tX2JhcihzdGF0PSdpZGVudGl0eScsIHBvc2l0aW9uPXBvc2l0aW9uX2RvZGdlKCkpICsgDQogICAgICAgc2NhbGVfZmlsbF9tYW51YWwodmFsdWVzPWNvbG9ycykgKyANCiAgICAgICBsYWJzKHk9J21lYW4gc2V2ZXJpdHkgc2NvcmUnKSkNCmdpdmFycyA8LSBjKCdoaXN0LnBqJywgJ2hpc3QuZGknLCAnaGlzdC5jbycpDQptcGlncyA8LSBtZWx0KHBpZ3NbLCBjKGlkdmFycywgZ2l2YXJzKV0sIGlkLnZhcnM9aWR2YXJzLCB2YXJpYWJsZS5uYW1lPSdzaXRlJykNCnRtcCA8LSBzdW1tYXJ5U0UobXBpZ3MsIG1lYXN1cmV2YXIgPSAndmFsdWUnLCBncm91cHZhcnMgPSBjKCdncm91cCcsICdzaXRlJyksIG5hLnJtPVRSVUUpDQp3aXRoKHRtcCwgZ2dwbG90KGRhdGE9dG1wLCBhZXMoeD1zaXRlLCBmaWxsPWdyb3VwLCB5PXZhbHVlKSkgKyANCiAgICAgICBnZW9tX2Vycm9yYmFyKGFlcyh5bWluID0gdmFsdWUgLSBzZSwgeW1heCA9IHZhbHVlICsgc2UpLCB3aWR0aCA9IDAuMiwgc2l6ZSA9IDEuNSwNCiAgICAgICAgICAgICAgICAgICAgIHBvc2l0aW9uID0gcG9zaXRpb25fZG9kZ2UoMC45KSkgKyANCiAgICAgICBnZW9tX2JhcihzdGF0PSdpZGVudGl0eScsIHBvc2l0aW9uPXBvc2l0aW9uX2RvZGdlKCkpICsgDQogICAgICAgc2NhbGVfZmlsbF9tYW51YWwodmFsdWVzPWNvbG9ycykgKyANCiAgICAgICBsYWJzKHk9J21lYW4gc2V2ZXJpdHkgc2NvcmUnKSkNCg0Kcm0oaWR2YXJzLCBnaXZhcnMsIG1waWdzLCB0bXApDQoNCiMgc2V2ZXJpdHkgc3RhdHMNCnNldnJlZyA8LSBnbG0oY2xpbi5zdCB+IGdyb3VwICsgd2VpZ2h0LmIgKyBmYXJtLCBmYW1pbHk9Z2F1c3NpYW4sIGRhdGE9cGlncykNCnN1bW1hcnkoZ2xodChzZXZyZWcsIGxpbmZjdCA9IG1jcChncm91cD0nVHVrZXknKSkpDQoNCnNldnJlZyA8LSBnbG0oY2xpbi5qZSB+IGdyb3VwICsgd2VpZ2h0LmIgKyBmYXJtLCBmYW1pbHk9Z2F1c3NpYW4sIGRhdGE9cGlncykNCnN1bW1hcnkoZ2xodChzZXZyZWcsIGxpbmZjdCA9IG1jcChncm91cD0nVHVrZXknKSkpDQoNCnNldnJlZyA8LSBnbG0oY2xpbi5pbCB+IGdyb3VwICsgd2VpZ2h0LmIgKyBmYXJtLCBmYW1pbHk9Z2F1c3NpYW4sIGRhdGE9cGlncykNCnN1bW1hcnkoZ2xodChzZXZyZWcsIGxpbmZjdCA9IG1jcChncm91cD0nVHVrZXknKSkpDQoNCnNldnJlZyA8LSBnbG0oY2xpbi5jbyB+IGdyb3VwICsgd2VpZ2h0LmIgKyBmYXJtLCBmYW1pbHk9Z2F1c3NpYW4sIGRhdGE9cGlncykNCnN1bW1hcnkoZ2xodChzZXZyZWcsIGxpbmZjdCA9IG1jcChncm91cD0nVHVrZXknKSkpDQoNCnNldnJlZyA8LSBnbG0oaGlzdC5waiB+IGdyb3VwICsgd2VpZ2h0LmIgKyBmYXJtLCBmYW1pbHk9Z2F1c3NpYW4sIGRhdGE9cGlncykNCnN1bW1hcnkoZ2xodChzZXZyZWcsIGxpbmZjdCA9IG1jcChncm91cD0nVHVrZXknKSkpDQoNCnNldnJlZyA8LSBnbG0oaGlzdC5kaSB+IGdyb3VwICsgd2VpZ2h0LmIgKyBmYXJtLCBmYW1pbHk9Z2F1c3NpYW4sIGRhdGE9cGlncykNCnN1bW1hcnkoZ2xodChzZXZyZWcsIGxpbmZjdCA9IG1jcChncm91cD0nVHVrZXknKSkpDQoNCnNldnJlZyA8LSBnbG0oaGlzdC5jbyB+IGdyb3VwICsgd2VpZ2h0LmIgKyBmYXJtLCBmYW1pbHk9Z2F1c3NpYW4sIGRhdGE9cGlncykNCnN1bW1hcnkoZ2xodChzZXZyZWcsIGxpbmZjdCA9IG1jcChncm91cD0nVHVrZXknKSkpDQoNCnJtKHNldnJlZywgcGlncykNCg0KYGBgDQoNCiMjIEZpZ3VyZSAyLiBNaWNyb2Jpb3RhIHJpY2huZXNzIGFuZCBkaXZlcnNpdHkNCg0KYGBge3J9DQojIGRlZmluZSBmaWxlIGxvY2F0aW9ucw0KYmlvbV9maWxlIDwtICdtb3RodXJfZmluYWwuYmlvbScgDQptYXBwaW5nX2ZpbGUgPC0gJ21hcHBpbmdmaWxlX3BoeWxvc2VxLmNzdicNCg0KIyBjcmVhdGUgcGh5bG9zZXEgb2JqZWN0IGZyb20gaW5wdXQgZmlsZXMNCnBzIDwtIGltcG9ydF9iaW9tKGJpb21fZmlsZSkNCnNhbXBsZV9uYW1lcyhwcykgPC0gbWFrZS5uYW1lcyhzYW1wbGVfbmFtZXMocHMpKQ0Kc2RhdGEgPC0gc2FtcGxlX2RhdGEobWVyZ2UocmVhZC5jc3YobWFwcGluZ19maWxlKSwNCiAgICAgICAgICAgICAgICAgICAgICAgICAgIGdldE1KTlBpZ3MoaW5jbC5uZXcgPSBGKVssIGMoJ3BpZ0lEJywgJ2dyb3VwJywgJ2hpc3QubmVjJyldLA0KICAgICAgICAgICAgICAgICAgICAgICAgICAgYnkgPSAncGlnSUQnKSkNCnJvdy5uYW1lcyhzZGF0YSkgPC0gbWFrZS5uYW1lcyhzZGF0YSRzYW1wbGVJRCkNCnBzIDwtIG1lcmdlX3BoeWxvc2VxKHBzLCBzZGF0YSkNCmNvbG5hbWVzKHRheF90YWJsZShwcykpIDwtIGMoJ0RvbWFpbicsICdQaHlsdW0nLCAnQ2xhc3MnLCAnT3JkZXInLCAnRmFtaWx5JywgJ0dlbnVzJykNCnNhbXBsZV9kYXRhKHBzKSRncm91cCA8LSBmYWN0b3Ioc2FtcGxlX2RhdGEocHMpJGdyb3VwLCBsZXZlbHMgPSBjKCdMQUMnLCAnTUlYJywgJ0NTUycpKQ0Kc2FtcGxlX2RhdGEocHMpJHNpdGUgPC0gZmFjdG9yKHNhbXBsZV9kYXRhKHBzKSRzaXRlLCBsZXZlbHMgPSBjKCdTdG9tYWNoJywgJ0lsZXVtJywgJ0NvbG9uJykpDQoNCiMgY2xlYW4gdXAgdGF4YSBuYW1lcw0KdGF4X3RhYmxlKHBzKSA8LSBnc3ViKCdfJywgJyAnLCBnc3ViKCdfXycsICcnLCB0YXhfdGFibGUocHMpKSkNCg0KIyByZW1vdmUgYW55IHNwdXJpb3VzIHRheGENCnBzIDwtIHN1YnNldF90YXhhKHBzLCBEb21haW4gPT0gJ0JhY3RlcmlhJykNCg0KIyBNYWtlIGEgZGF0YSBmcmFtZSB3aXRoIGEgY29sdW1uIGZvciB0aGUgcmVhZCBjb3VudHMgb2YgZWFjaCBzYW1wbGUNCnNhbXBsZV9zdW1fZGYgPC0gZGF0YS5mcmFtZShzdW0gPSBzYW1wbGVfc3VtcyhwcykpIA0KDQojIEhpc3RvZ3JhbSBvZiBzYW1wbGUgcmVhZCBjb3VudHMNCiMgICBieSBzYW1wbGUgdHlwZSAodGlzc3VlIHYgY29udGVudHMpDQpnZ3Bsb3QobWVyZ2Uoc2FtcGxlX3N1bV9kZiwgc2FtcGxlX2RhdGEocHMpLCBieSA9ICdyb3cubmFtZXMnKSwgYWVzKHggPSBzdW0pKSArICBmYWNldF9ncmlkKCB+IHR5cGUpICsNCiAgZ2VvbV9oaXN0b2dyYW0oYmlud2lkdGggPSA1MDApICsNCiAgZ2d0aXRsZSgnRGlzdHJpYnV0aW9uIG9mIHNhbXBsZSBzZXF1ZW5jaW5nIGRlcHRoIChyZWQgbGluZSA9IDIwMDApJykgKyANCiAgeGxhYignUmVhZCBjb3VudHMgKGJpbj01MDApJykgKw0KICB4bGltKC01MDAsIE5BKSArICAjIC01MDAgdG8gZW5zdXJlIGFsbCBkYXRhIGFyZSBkaXNwbGF5ZWQgb24gY2hhcnQ7IE5BIHRvIHNldCBsaW1pdCB0byBkYXRhIG1heA0KICBnZW9tX3ZsaW5lKHhpbnRlcmNlcHQgPSAyMDAwLCBsaW5ldHlwZSA9ICdkYXNoZWQnLCBjb2xvciA9ICdyZWQnKSArDQogIHRoZW1lKGF4aXMudGl0bGUueSA9IGVsZW1lbnRfYmxhbmsoKSkNCiMgICBieSBhbGwgY2F0ZWdvcmllcyAoc2l0ZSwgdHlwZSwgZ3JvdXAsIGhpc3QubmVjKQ0KZ2dwbG90KG1lcmdlKHNhbXBsZV9zdW1fZGYsIHNhbXBsZV9kYXRhKHBzKSwgYnkgPSAncm93Lm5hbWVzJyksIGFlcyh4ID0gc3VtKSkgKw0KICBmYWNldF9ncmlkKHR5cGUgKyBncm91cCB+IHNpdGUgKyBoaXN0Lm5lYykgKw0KICBnZW9tX2hpc3RvZ3JhbShiaW53aWR0aCA9IDUwMCkgKyB0aGVtZV9idygpICsNCiAgZ2d0aXRsZSgnRGlzdHJpYnV0aW9uIG9mIHNhbXBsZSBzZXF1ZW5jaW5nIGRlcHRoIChyZWQgbGluZSA9IDIwMDApJykgKyANCiAgeGxhYignUmVhZCBjb3VudHMgKGJpbj01MDApJykgKw0KICB4bGltKC01MDAsIE5BKSArICAjIC01MDAgdG8gZW5zdXJlIGFsbCBkYXRhIGFyZSBkaXNwbGF5ZWQgb24gY2hhcnQ7IE5BIHRvIHNldCBsaW1pdCB0byBkYXRhIG1heA0KICBnZW9tX3ZsaW5lKHhpbnRlcmNlcHQgPSAyMDAwLCBsaW5ldHlwZSA9ICdkYXNoZWQnLCBjb2xvciA9ICdyZWQnKSArDQogIHRoZW1lKGF4aXMudGl0bGUueSA9IGVsZW1lbnRfYmxhbmsoKSkNCg0KIyByZW1vdmUgc2FtcGxlcyB3aXRoIGxvdyByZWFkIGNvdW50cw0KcHMgPC0gcHJ1bmVfc2FtcGxlcyhzYW1wbGVfc3VtcyhwcykgPj0gMjAwMCwgcHMpICAjIHRoZXJlIGFyZW4ndCBhbnkgaW4gdGhpcyBkYXRhDQoNCiMgc3Vic2V0IGludG8gdGlzc3VlIGFuZCBjb250ZW50cw0KcHMudCA8LSBzdWJzZXRfc2FtcGxlcyhwcywgdHlwZSA9PSAnVGlzc3VlJykNCnBzLmMgPC0gc3Vic2V0X3NhbXBsZXMocHMsIHR5cGUgPT0gJ0NvbnRlbnRzJykNCg0KIyBzdWJzZXQgaW50byBIZWFsdGh5IGFuZCBORUMgKGhpc3QubmVjKQ0KcHMudC5oIDwtIHN1YnNldF9zYW1wbGVzKHBzLnQsIGhpc3QubmVjID09ICdIZWFsdGh5JykNCnBzLnQubiA8LSBzdWJzZXRfc2FtcGxlcyhwcy50LCBoaXN0Lm5lYyA9PSAnTkVDJykNCnBzLmMuaCA8LSBzdWJzZXRfc2FtcGxlcyhwcy5jLCBoaXN0Lm5lYyA9PSAnSGVhbHRoeScpDQpwcy5jLm4gPC0gc3Vic2V0X3NhbXBsZXMocHMuYywgaGlzdC5uZWMgPT0gJ05FQycpDQoNCiMgcmVtb3ZlIE9UVXMgd2hpY2ggYXJlIHByZXNlbnQgaW4gMCBzYW1wbGVzDQpwcyA8LSBwcnVuZV90YXhhKHRheGFfc3VtcyhwcykgPiAwLCBwcykNCnBzLnQgPC0gcHJ1bmVfdGF4YSh0YXhhX3N1bXMocHMudCkgPiAwLCBwcy50KQ0KcHMudC5oIDwtIHBydW5lX3RheGEodGF4YV9zdW1zKHBzLnQuaCkgPiAwLCBwcy50LmgpDQpwcy50Lm4gPC0gcHJ1bmVfdGF4YSh0YXhhX3N1bXMocHMudC5uKSA+IDAsIHBzLnQubikNCnBzLmMgPC0gcHJ1bmVfdGF4YSh0YXhhX3N1bXMocHMuYykgPiAwLCBwcy5jKQ0KcHMuYy5oIDwtIHBydW5lX3RheGEodGF4YV9zdW1zKHBzLmMuaCkgPiAwLCBwcy5jLmgpDQpwcy5jLm4gPC0gcHJ1bmVfdGF4YSh0YXhhX3N1bXMocHMuYy5uKSA+IDAsIHBzLmMubikNCg0KcmljaC5jIDwtIG1lcmdlKGVzdGltYXRlX3JpY2huZXNzKHBzLmMsIG1lYXN1cmVzID0gYygnT2JzZXJ2ZWQnLCAnSW52U2ltcHNvbicpKSwNCiAgICAgICAgICAgICAgICBzYW1wbGVfZGF0YShwcy5jKSwgYnkgPSAncm93Lm5hbWVzJykNCnJpY2gudCA8LSBtZXJnZShlc3RpbWF0ZV9yaWNobmVzcyhwcy50LCBtZWFzdXJlcyA9IGMoJ09ic2VydmVkJywgJ0ludlNpbXBzb24nKSksDQogICAgICAgICAgICAgICAgc2FtcGxlX2RhdGEocHMudCksIGJ5ID0gJ3Jvdy5uYW1lcycpDQpyaWNoLmMuaCA8LSBtZXJnZShlc3RpbWF0ZV9yaWNobmVzcyhwcy5jLmgsIG1lYXN1cmVzID0gYygnT2JzZXJ2ZWQnLCAnSW52U2ltcHNvbicpKSwNCiAgICAgICAgICAgICAgICAgIHNhbXBsZV9kYXRhKHBzLmMuaCksIGJ5ID0gJ3Jvdy5uYW1lcycpDQpyaWNoLmMubiA8LSBtZXJnZShlc3RpbWF0ZV9yaWNobmVzcyhwcy5jLm4sIG1lYXN1cmVzID0gYygnT2JzZXJ2ZWQnLCAnSW52U2ltcHNvbicpKSwNCiAgICAgICAgICAgICAgICAgIHNhbXBsZV9kYXRhKHBzLmMubiksIGJ5ID0gJ3Jvdy5uYW1lcycpDQpyaWNoLnQuaCA8LSBtZXJnZShlc3RpbWF0ZV9yaWNobmVzcyhwcy50LmgsIG1lYXN1cmVzID0gYygnT2JzZXJ2ZWQnLCAnSW52U2ltcHNvbicpKSwNCiAgICAgICAgICAgICAgICAgIHNhbXBsZV9kYXRhKHBzLnQuaCksIGJ5ID0gJ3Jvdy5uYW1lcycpDQpyaWNoLnQubiA8LSBtZXJnZShlc3RpbWF0ZV9yaWNobmVzcyhwcy50Lm4sIG1lYXN1cmVzID0gYygnT2JzZXJ2ZWQnLCAnSW52U2ltcHNvbicpKSwNCiAgICAgICAgICAgICAgICAgIHNhbXBsZV9kYXRhKHBzLnQubiksIGJ5ID0gJ3Jvdy5uYW1lcycpDQoNCiMgdGVzdCBmb3Igc2lnbmlmaWNhbnQgZGlmZmVyZW5jZXMgaW4gcmljaG5lc3MNCmxhcHBseShzcGxpdC5kYXRhLmZyYW1lKHJpY2guYy5oLCByaWNoLmMuaCRzaXRlLCBkcm9wID0gVCksDQogICAgICAgZnVuY3Rpb24oeCkgeyBzdW1tYXJ5KGdsaHQoZ2xtKA0KICAgICAgICAgT2JzZXJ2ZWQgfiBncm91cCwgZmFtaWx5ID0gZ2F1c3NpYW4sIGRhdGEgPSB4KSwgbGluZmN0ID0gbWNwKGdyb3VwID0gJ1R1a2V5JykpKSB9ICkNCmxhcHBseShzcGxpdC5kYXRhLmZyYW1lKHJpY2guYy5uLCByaWNoLmMubiRzaXRlLCBkcm9wID0gVCksDQogICAgICAgZnVuY3Rpb24oeCkgeyBzdW1tYXJ5KGdsaHQoZ2xtKA0KICAgICAgICAgT2JzZXJ2ZWQgfiBncm91cCwgZmFtaWx5ID0gZ2F1c3NpYW4sIGRhdGEgPSB4KSwgbGluZmN0ID0gbWNwKGdyb3VwID0gJ1R1a2V5JykpKSB9ICkNCmxhcHBseShzcGxpdC5kYXRhLmZyYW1lKHJpY2gudC5oLCByaWNoLnQuaCRzaXRlLCBkcm9wID0gVCksDQogICAgICAgZnVuY3Rpb24oeCkgeyBzdW1tYXJ5KGdsaHQoZ2xtKA0KICAgICAgICAgT2JzZXJ2ZWQgfiBncm91cCwgZmFtaWx5ID0gZ2F1c3NpYW4sIGRhdGEgPSB4KSwgbGluZmN0ID0gbWNwKGdyb3VwID0gJ1R1a2V5JykpKSB9ICkNCmxhcHBseShzcGxpdC5kYXRhLmZyYW1lKHJpY2gudC5uLCByaWNoLnQubiRzaXRlLCBkcm9wID0gVCksDQogICAgICAgZnVuY3Rpb24oeCkgeyBzdW1tYXJ5KGdsaHQoZ2xtKA0KICAgICAgICAgT2JzZXJ2ZWQgfiBncm91cCwgZmFtaWx5ID0gZ2F1c3NpYW4sIGRhdGEgPSB4KSwgbGluZmN0ID0gbWNwKGdyb3VwID0gJ1R1a2V5JykpKSB9ICkNCiMgdGVzdCBmb3Igc2lnbmlmaWNhbnQgZGlmZmVyZW5jZXMgaW4gYWxwaGEgZGl2ZXJzaXR5DQpsYXBwbHkoc3BsaXQuZGF0YS5mcmFtZShyaWNoLmMuaCwgcmljaC5jLmgkc2l0ZSwgZHJvcCA9IFQpLA0KICAgICAgIGZ1bmN0aW9uKHgpIHsgc3VtbWFyeShnbGh0KGdsbShJbnZTaW1wc29uIH4gZ3JvdXAsIGZhbWlseSA9IGdhdXNzaWFuLCBkYXRhID0geCksDQogICAgICAgICAgICAgICAgICAgICAgICAgICAgICAgICAgbGluZmN0ID0gbWNwKGdyb3VwID0gJ1R1a2V5JykpKSB9ICkNCmxhcHBseShzcGxpdC5kYXRhLmZyYW1lKHJpY2guYy5uLCByaWNoLmMubiRzaXRlLCBkcm9wID0gVCksDQogICAgICAgZnVuY3Rpb24oeCkgeyBzdW1tYXJ5KGdsaHQoZ2xtKEludlNpbXBzb24gfiBncm91cCwgZmFtaWx5ID0gZ2F1c3NpYW4sIGRhdGEgPSB4KSwNCiAgICAgICAgICAgICAgICAgICAgICAgICAgICAgICAgICBsaW5mY3QgPSBtY3AoZ3JvdXAgPSAnVHVrZXknKSkpIH0gKQ0KbGFwcGx5KHNwbGl0LmRhdGEuZnJhbWUocmljaC50LmgsIHJpY2gudC5oJHNpdGUsIGRyb3AgPSBUKSwNCiAgICAgICBmdW5jdGlvbih4KSB7IHN1bW1hcnkoZ2xodChnbG0oSW52U2ltcHNvbiB+IGdyb3VwLCBmYW1pbHkgPSBnYXVzc2lhbiwgZGF0YSA9IHgpLA0KICAgICAgICAgICAgICAgICAgICAgICAgICAgICAgICAgIGxpbmZjdCA9IG1jcChncm91cCA9ICdUdWtleScpKSkgfSApDQpsYXBwbHkoc3BsaXQuZGF0YS5mcmFtZShyaWNoLnQubiwgcmljaC50Lm4kc2l0ZSwgZHJvcCA9IFQpLA0KICAgICAgIGZ1bmN0aW9uKHgpIHsgc3VtbWFyeShnbGh0KGdsbShJbnZTaW1wc29uIH4gZ3JvdXAsIGZhbWlseSA9IGdhdXNzaWFuLCBkYXRhID0geCksDQogICAgICAgICAgICAgICAgICAgICAgICAgICAgICAgICAgbGluZmN0ID0gbWNwKGdyb3VwID0gJ1R1a2V5JykpKSB9ICkNCg0KIyBwbG90IE9ic2VydmVkIE9UVXMgKEFsbC1mZWQgcGlncyB0b2dldGhlcikgYnkgZm9ybXVsYSBncm91cCBhbmQgYnkgTkVDL2hlYWx0aHkNCiMgICBjb250ZW50cw0KZ2cxIDwtIGdncGxvdChyaWNoLmMsIGFlcyh4ID0gc2l0ZSwgeSA9IE9ic2VydmVkLCBjb2xvciA9IGdyb3VwKSkgKw0KICBnZW9tX2JveHBsb3QobHdkID0gMSwgcG9zaXRpb24gPSBwb3NpdGlvbl9kb2RnZSh3aWR0aCA9IDAuODUpLCB3aWR0aCA9IDAuNywgb3V0bGllci5zaGFwZSA9IE5BKSArDQogIGdlb21fcG9pbnQoc2hhcGUgPSAxOSwgcG9zaXRpb24gPSBwb3NpdGlvbl9qaXR0ZXJkb2RnZShqaXR0ZXIud2lkdGggPSAwLjE1LCBkb2RnZS53aWR0aCA9IDAuODUpKSArDQogIHNjYWxlX2NvbG91cl9tYW51YWwodmFsdWVzID0gZ3JvdXBfY29sb3JzKSArDQogIGxhYnMobGlzdCh0aXRsZSA9ICdPYnMsIEFsbC1mZWQsIENvbnRlbnRzJywgeCA9IGVsZW1lbnRfYmxhbmsoKSwgeSA9IGVsZW1lbnRfYmxhbmsoKSkpICsNCiAgeWxpbSgwLCAxNTApDQpnZzIgPC0gZ2dwbG90KHJpY2guYywgYWVzKHggPSBzaXRlLCB5ID0gT2JzZXJ2ZWQsIGNvbG9yID0gaGlzdC5uZWMpKSArDQogIGdlb21fYm94cGxvdChsd2QgPSAxLCBwb3NpdGlvbiA9IHBvc2l0aW9uX2RvZGdlKHdpZHRoID0gMC44NSksIHdpZHRoID0gMC43LCBvdXRsaWVyLnNoYXBlID0gTkEpICsNCiAgZ2VvbV9wb2ludChzaGFwZSA9IDE5LCBwb3NpdGlvbiA9IHBvc2l0aW9uX2ppdHRlcmRvZGdlKGppdHRlci53aWR0aCA9IDAuMTUsIGRvZGdlLndpZHRoID0gMC44NSkpICsNCiAgc2NhbGVfY29sb3VyX21hbnVhbCh2YWx1ZXMgPSBobl9jb2xvcnMpICsgeWxpbSgwLCAxNTApICsNCiAgbGFicyhsaXN0KHRpdGxlID0gJ09icywgQWxsLWZlZCwgQ29udGVudHMnLCB4ID0gZWxlbWVudF9ibGFuaygpLCB5ID0gZWxlbWVudF9ibGFuaygpKSkgDQpncmlkLmFycmFuZ2UoZ2cxLCBnZzIsIG5jb2wgPSAyKSAgDQojICAgdGlzc3VlDQpnZzEgPC0gZ2dwbG90KHJpY2gudCwgYWVzKHggPSBzaXRlLCB5ID0gT2JzZXJ2ZWQsIGNvbG9yID0gZ3JvdXApKSArDQogIGdlb21fYm94cGxvdChsd2QgPSAxLCBwb3NpdGlvbiA9IHBvc2l0aW9uX2RvZGdlKHdpZHRoID0gMC44NSksIHdpZHRoID0gMC43LCBvdXRsaWVyLnNoYXBlID0gTkEpICsNCiAgZ2VvbV9wb2ludChzaGFwZSA9IDE5LCBwb3NpdGlvbiA9IHBvc2l0aW9uX2ppdHRlcmRvZGdlKGppdHRlci53aWR0aCA9IDAuMTUsIGRvZGdlLndpZHRoID0gMC44NSkpICsNCiAgc2NhbGVfY29sb3VyX21hbnVhbCh2YWx1ZXMgPSBncm91cF9jb2xvcnMpICsNCiAgbGFicyhsaXN0KHRpdGxlID0gJ09icywgQWxsLWZlZCwgVGlzc3VlIE11Y29zYScsIHggPSBlbGVtZW50X2JsYW5rKCksIHkgPSBlbGVtZW50X2JsYW5rKCkpKSArDQogIHlsaW0oMCwgMTUwKQ0KZ2cyIDwtIGdncGxvdChyaWNoLnQsIGFlcyh4ID0gc2l0ZSwgeSA9IE9ic2VydmVkLCBjb2xvciA9IGhpc3QubmVjKSkgKw0KICBnZW9tX2JveHBsb3QobHdkID0gMSwgcG9zaXRpb24gPSBwb3NpdGlvbl9kb2RnZSh3aWR0aCA9IDAuODUpLCB3aWR0aCA9IDAuNywgb3V0bGllci5zaGFwZSA9IE5BKSArDQogIGdlb21fcG9pbnQoc2hhcGUgPSAxOSwgcG9zaXRpb24gPSBwb3NpdGlvbl9qaXR0ZXJkb2RnZShqaXR0ZXIud2lkdGggPSAwLjE1LCBkb2RnZS53aWR0aCA9IDAuODUpKSArDQogIHNjYWxlX2NvbG91cl9tYW51YWwodmFsdWVzID0gaG5fY29sb3JzKSArIHlsaW0oMCwgMTUwKSArDQogIGxhYnMobGlzdCh0aXRsZSA9ICdPYnMsIEFsbC1mZWQsIFRpc3N1ZSBNdWNvc2EnLCB4ID0gZWxlbWVudF9ibGFuaygpLCB5ID0gZWxlbWVudF9ibGFuaygpKSkgDQpncmlkLmFycmFuZ2UoZ2cxLCBnZzIsIG5jb2wgPSAyKSAgDQoNCiMgdGVzdCBPYnNlcnZlZCBPVFVzIChBbGwtZmVkIHBpZ3MgdG9nZXRoZXIpIGJ5IGZvcm11bGEgZ3JvdXAgYW5kIGJ5IE5FQy9oZWFsdGh5DQojICAgY29udGVudHMNCmxhcHBseShzcGxpdC5kYXRhLmZyYW1lKHJpY2guYywgcmljaC5jJHNpdGUsIGRyb3AgPSBUKSwNCiAgICAgICBmdW5jdGlvbih4KSB7IHN1bW1hcnkoZ2xodChnbG0oDQogICAgICAgICBPYnNlcnZlZCB+IGdyb3VwLCBmYW1pbHkgPSBnYXVzc2lhbiwgZGF0YSA9IHgpLCBsaW5mY3QgPSBtY3AoZ3JvdXAgPSAnVHVrZXknKSkpIH0gKQ0KbGFwcGx5KHNwbGl0LmRhdGEuZnJhbWUocmljaC5jLCByaWNoLmMkc2l0ZSwgZHJvcCA9IFQpLA0KICAgICAgIGZ1bmN0aW9uKHgpIHsgc3VtbWFyeShnbGh0KGdsbSgNCiAgICAgICAgIE9ic2VydmVkIH4gaGlzdC5uZWMsIGZhbWlseSA9IGdhdXNzaWFuLCBkYXRhID0geCksIGxpbmZjdCA9IG1jcChoaXN0Lm5lYyA9ICdUdWtleScpKSkgfSApDQojICAgdGlzc3VlDQpsYXBwbHkoc3BsaXQuZGF0YS5mcmFtZShyaWNoLnQsIHJpY2gudCRzaXRlLCBkcm9wID0gVCksDQogICAgICAgZnVuY3Rpb24oeCkgeyBzdW1tYXJ5KGdsaHQoZ2xtKA0KICAgICAgICAgT2JzZXJ2ZWQgfiBncm91cCwgZmFtaWx5ID0gZ2F1c3NpYW4sIGRhdGEgPSB4KSwgbGluZmN0ID0gbWNwKGdyb3VwID0gJ1R1a2V5JykpKSB9ICkNCmxhcHBseShzcGxpdC5kYXRhLmZyYW1lKHJpY2gudCwgcmljaC50JHNpdGUsIGRyb3AgPSBUKSwNCiAgICAgICBmdW5jdGlvbih4KSB7IHN1bW1hcnkoZ2xodChnbG0oDQogICAgICAgICBPYnNlcnZlZCB+IGhpc3QubmVjLCBmYW1pbHkgPSBnYXVzc2lhbiwgZGF0YSA9IHgpLCBsaW5mY3QgPSBtY3AoaGlzdC5uZWMgPSAnVHVrZXknKSkpIH0gKQ0KDQojIGdldCBtZWRpYW4gdmFsdWVzIG9mIG9ic2VydmVkIE9UVXMgZm9yIGNvbG9uIHNhbXBsZXMNCndpdGgocmljaC5jW3JpY2guYyRzaXRlID09ICdDb2xvbicsIF0sIGFnZ3JlZ2F0ZShPYnNlcnZlZCB+IGhpc3QubmVjOmdyb3VwLCBGVU49bWVkaWFuKSkgDQoNCnJtKHJpY2guYywgcmljaC5jLmgsIHJpY2guYy5uLCByaWNoLnQsIHJpY2gudC5oLCByaWNoLnQubiwgZ2cxLCBnZzIsIHNhbXBsZV9zdW1fZGYsIA0KICAgc2RhdGEsIGJpb21fZmlsZSwgbWFwcGluZ19maWxlKQ0KDQpgYGANCg0KIyMgT3ZlcnZpZXcgLSBjbGFzcyBsZXZlbA0KDQpgYGB7cn0NCiMgc3Vic2FtcGxlIHRvIGV2ZW4gcmVhZCBkZXB0aA0KcHMudC5yIDwtIHJhcmVmeV9ldmVuX2RlcHRoKHBzLnQsIHJuZ3NlZWQgPSAyNTAxLCByZXBsYWNlID0gRkFMU0UpDQpwcy5jLnIgPC0gcmFyZWZ5X2V2ZW5fZGVwdGgocHMuYywgcm5nc2VlZCA9IDI1MDEsIHJlcGxhY2UgPSBGQUxTRSkNCg0KIyBzdWJzZXQgYnkgR0kgdHJhY3Qgc2l0ZQ0KcHMubGlzdCA8LSBsaXN0KCdJbGV1bSBUaXNzdWUnID0gc3Vic2V0X3NhbXBsZXMocHMudC5yLCBzaXRlID09ICdJbGV1bScpLA0KICAgICAgICAgICAgICAgICdDb2xvbiBUaXNzdWUnID0gc3Vic2V0X3NhbXBsZXMocHMudC5yLCBzaXRlID09ICdDb2xvbicpLA0KICAgICAgICAgICAgICAgICdTdG9tYWNoIENvbnRlbnRzJyA9IHN1YnNldF9zYW1wbGVzKHBzLmMuciwgc2l0ZSA9PSAnU3RvbWFjaCcpLA0KICAgICAgICAgICAgICAgICdJbGV1bSBDb250ZW50cycgPSBzdWJzZXRfc2FtcGxlcyhwcy5jLnIsIHNpdGUgPT0gJ0lsZXVtJyksDQogICAgICAgICAgICAgICAgJ0NvbG9uIENvbnRlbnRzJyA9IHN1YnNldF9zYW1wbGVzKHBzLmMuciwgc2l0ZSA9PSAnQ29sb24nKSkNCg0KIyB0cmFuc2Zvcm0gYWJ1bmRhbmNlcyBmcm9tIGFic29sdXRlIHRvIHJlbGF0aXZlIChwcm9wb3J0aW9ucykNCnBzLmxpc3QgPC0gbGxwbHkocHMubGlzdCwgZnVuY3Rpb24oeCkgew0KICB0cmFuc2Zvcm1fc2FtcGxlX2NvdW50cyh4LCBmdW5jdGlvbih5KSB5IC8gc3VtKHkpKSB9KQ0KcHMudC5yIDwtIHRyYW5zZm9ybV9zYW1wbGVfY291bnRzKHBzLnQuciwgZnVuY3Rpb24oeCkgeCAvIHN1bSh4KSkNCnBzLmMuciA8LSB0cmFuc2Zvcm1fc2FtcGxlX2NvdW50cyhwcy5jLnIsIGZ1bmN0aW9uKHgpIHggLyBzdW0oeCkpDQoNCiMgYWdnbG9tZXJhdGUgT1RVcyBieSBjbGFzcw0KcHMubGlzdC5jbGFzcyA8LSBsbHBseShwcy5saXN0LCBmdW5jdGlvbih4KSB7IHRheF9nbG9tKHgsICdDbGFzcycpIH0pDQpvdHVzIDwtIGxscGx5KHBzLmxpc3QuY2xhc3MsIGZ1bmN0aW9uKHgpIHsgcm93Lm5hbWVzKHRheF90YWJsZSh4KSkgfSkNCmtlZXAgPC0gbGxwbHkobmFtZXMocHMubGlzdC5jbGFzcyksIGZ1bmN0aW9uKHgpIHsNCiAgcm93Lm5hbWVzKHRheF90YWJsZShwcy5saXN0LmNsYXNzW1t4XV0pKVt3aGljaCh0YXhfdGFibGUocHMubGlzdC5jbGFzc1tbeF1dKVsgLCAnQ2xhc3MnXSANCiAgICAgICAgICAgICAgICAgICAgICAgICAgICAgICAgICAgICAgICAgICAgICAgICAlaW4lIGMoJ0dhbW1hcHJvdGVvYmFjdGVyaWEnLCAnQ2xvc3RyaWRpYScsICdCYWNpbGxpJykpXSB9KQ0KDQojIGNvbWJpbmUgc21hbGwgdGF4YSBpbnRvICdPdGhlcicNCm5hbWVzKGtlZXApIDwtIG5hbWVzKHBzLmxpc3QuY2xhc3MpDQpwcy5saXN0LmNsYXNzLm8gPC0gbGxwbHkobmFtZXMocHMubGlzdC5jbGFzcyksIGZ1bmN0aW9uKHgpIHsgDQogIG1lcmdlX3RheGEocHMubGlzdC5jbGFzc1tbeF1dLCBzZXRkaWZmKG90dXNbW3hdXSwga2VlcFtbeF1dKSkgfSkNCm5hbWVzKHBzLmxpc3QuY2xhc3MubykgPC0gbmFtZXMocHMubGlzdC5jbGFzcykNCmZvcih4IGluIHNlcV9hbG9uZyhwcy5saXN0LmNsYXNzLm8pKSB7DQogIHRheF90YWJsZShwcy5saXN0LmNsYXNzLm9bW3hdXSlbaXMubmEodGF4X3RhYmxlKHBzLmxpc3QuY2xhc3Mub1tbeF1dKVsgLCAnQ2xhc3MnXSksIDI6M10gPC0gYygnT3RoZXInLCAnT3RoZXInKQ0KfQ0KDQpybShrZWVwLCBvdHVzLCB4LCBwcy5saXN0LmNsYXNzLm8pDQoNCmBgYA0KDQoNCiMjIEZpZ3VyZXMgUzMgYW5kIFM0LiBGb3JtdWxhIGdyb3VwcyAoYWxsIGZlZCkgLSBjbGFzcyBsZXZlbA0KDQpgYGB7cn0NCiMgdG9wIE4gbnVtYmVyIG9mIHRheGEgdG8gY29tcGFyZQ0KdG9wTiA8LSA0DQoNCiMgcHJlcGFyZSB0byBwbG90IHRvcCBOICJDTEFTUyIgaW4gYm94cGxvdHMNCnBzbS5saXN0LmNsYXNzIDwtIGxhcHBseShwcy5saXN0LmNsYXNzLCBwc21lbHQpDQoNCiMgZ2V0IHRvcCBOIHRheGEgcmFua2VkIGJ5IGRlY3JlYXNpbmcgbWVhbiBhYnVuZGFuY2UNCm1lYW5zIDwtIGxhcHBseShwc20ubGlzdC5jbGFzcywgZnVuY3Rpb24oeCl7IGRkcGx5KHgsIH4gQ2xhc3MsDQogICAgICAgICAgICAgICAgICAgICAgICAgICAgICAgICAgICAgICAgICAgICAgICAgICBmdW5jdGlvbih5KXsgYyhtZWFuID0gbWVhbih5JEFidW5kYW5jZSkpIH0pIH0pDQptZWFucyA8LSBsYXBwbHkobWVhbnMsIGZ1bmN0aW9uKHgpeyB4W3JldihvcmRlcih4JG1lYW4pKSwgXSB9KQ0KdG9wTi50YXhhIDwtIGxhcHBseShtZWFucywgZnVuY3Rpb24oeCl7IHhbMTp0b3BOLCAnQ2xhc3MnXSB9KQ0KdG9wTi50YXhhIDwtIGxhcHBseSh0b3BOLnRheGEsIGZ1bmN0aW9uKHgpIHsgdG9wTi50YXhhW1snQ29sb24gQ29udGVudHMnXV0gfSkgIyBmb3JjZSBzYW1lIG9yZGVyDQpwc20ubGlzdC5jbGFzcyA8LSBtYXBwbHkoZnVuY3Rpb24oeCwgaSl7IHhbeCRDbGFzcyAlaW4lIHRvcE4udGF4YVtbaV1dLCBdIH0sIHBzbS5saXN0LmNsYXNzLCBzZXFfYWxvbmcocHNtLmxpc3QuY2xhc3MpLA0KICAgICAgICAgICAgICAgICAgICAgICAgIFNJTVBMSUZZID0gRkFMU0UsIFVTRS5OQU1FUyA9IFRSVUUpDQoNCg0KIyBkZXRlcm1pbmUgaWYgYW55IGRpZmZlcmVuY2VzIGFjcm9zcyBncm91cHMNCmt3LnB2YWxzIDwtIGxhcHBseShwc20ubGlzdC5jbGFzcywgZnVuY3Rpb24oeCl7IGRkcGx5KHgsIH4gQ2xhc3MsIGZ1bmN0aW9uKHkpIHsNCiAgYyhwcm9iID0ga3J1c2thbC50ZXN0KGRhdGEgPSB5LCBBYnVuZGFuY2UgfiBncm91cCkkcC52YWx1ZSkgfSkgfSkNCmZvcihpIGluIHNlcV9hbG9uZyhrdy5wdmFscykpIHsNCiAga3cucHZhbHNbW2ldXSRwcm9iIDwtIHAuYWRqdXN0KGt3LnB2YWxzW1tpXV0kcHJvYiwgJ2hvbG0nKQ0KfQ0KIyBnZXQgbGFiZWxzIGZvciB0YXhhIHdoaWNoIGFyZSBkaWZmZXJlbnQNCnNpZ3MgPC0gbGxwbHkobmFtZXMoa3cucHZhbHMpLCBmdW5jdGlvbih4KSB7a3cucHZhbHNbW3hdXSRwcm9iIDw9IDAuMDV9KQ0KbmFtZXMoc2lncykgPC0gbmFtZXMoa3cucHZhbHMpDQpzaWdzIDwtIGxscGx5KG5hbWVzKHNpZ3MpLCBmdW5jdGlvbih4KSB7IGlmKGFueShzaWdzW1t4XV0pKSBrdy5wdmFsc1tbeF1dW3NpZ3NbW3hdXSwgJ0NsYXNzJ10gZWxzZSBOQSB9KQ0KbmFtZXMoc2lncykgPC0gbmFtZXMoa3cucHZhbHMpDQoNCmZvcihpIGluIHNlcV9hbG9uZyhwc20ubGlzdC5jbGFzcykpIHsNCiAgcGxvdCA8LSBnZ3Bsb3QoZGF0YS5mcmFtZShwc20ubGlzdC5jbGFzc1tbaV1dKSwgYWVzKHggPSBDbGFzcywgeSA9IEFidW5kYW5jZSwgY29sb3IgPSBncm91cCkpICsgDQogICAgZ2VvbV9ib3hwbG90KGx3ZCA9IDEsIHBvc2l0aW9uID0gcG9zaXRpb25fZG9kZ2Uod2lkdGggPSAwLjc1KSwgd2lkdGggPSAwLjcsDQogICAgICAgICAgICAgICAgIG91dGxpZXIuc2hhcGUgPSBOQSkgKw0KICAgIGdlb21fcG9pbnQoc2hhcGUgPSAxOSwgcG9zaXRpb24gPSBwb3NpdGlvbl9qaXR0ZXJkb2RnZShqaXR0ZXIud2lkdGggPSAwLjE1LA0KICAgICAgICAgICAgICAgICAgICAgICAgICAgICAgICAgICAgICAgICAgICAgICAgICAgICAgICAgICBkb2RnZS53aWR0aCA9IDAuNzUpKSArDQogICAgc2NhbGVfeF9kaXNjcmV0ZShsaW1pdHMgPSB1bmxpc3QodG9wTi50YXhhW2ldKSkgKw0KICAgIHNjYWxlX3lfc3FydChicmVha3MgPSBjKDEsIDAuNjQsIDAuMzYsIDAuMTYsIDAuMDQsIDAuMCksIGxpbWl0cyA9IGMoMCwgMS4xKSwgbGFiZWxzID0gcGVyY2VudCkgKw0KICAgIHNjYWxlX2NvbG91cl9tYW51YWwodmFsdWVzID0gZ3JvdXBfY29sb3JzKSArIA0KICAgIGFubm90YXRlKCd0ZXh0JywgeCA9IHVubGlzdChzaWdzW2ldKSwgbmEucm0gPSBUUlVFLCB5ID0gMS4wNSwgbGFiZWwgPSAnKicsIHNpemUgPSAxMikgKw0KICAgIHRoZW1lKGF4aXMudGV4dC54ID0gZWxlbWVudF90ZXh0KGFuZ2xlID0gLTQ1LCBoanVzdCA9IDAsIHZqdXN0ID0gMSkpICsNCiAgICBsYWJzKGxpc3QodGl0bGUgPSBuYW1lcyhwc20ubGlzdC5jbGFzcylbaV0sIHggPSBlbGVtZW50X2JsYW5rKCksIHkgPSBlbGVtZW50X3RleHQoJ1JlbGF0aXZlIEFidW5kYW5jZScpKSkgKw0KICAgIHRoZW1lKGxlZ2VuZC5wb3NpdGlvbiA9ICdyaWdodCcsIGxlZ2VuZC50aXRsZSA9IGVsZW1lbnRfYmxhbmsoKSwgcGxvdC5tYXJnaW4gPSBtYXJnaW4oMTAsIDEwLCAxMCwgMjUpKQ0KICBwcmludChwbG90KQ0KfQ0KDQojIHRlc3QgZm9yIGRpZmZlcmVuY2UgaW4gQmFjaWxsaSBiZXR3ZWVuIEdJIGNvbnRlbnRzIHNpdGVzDQpzYyA8LSBwc20ubGlzdC5jbGFzcyQnU3RvbWFjaCBDb250ZW50cycNCmljIDwtIHBzbS5saXN0LmNsYXNzJCdJbGV1bSBDb250ZW50cycNCmNjIDwtIHBzbS5saXN0LmNsYXNzJCdDb2xvbiBDb250ZW50cycNCmJhY2lsbGkubGlzdCA8LSBsaXN0KHNjW3NjJENsYXNzID09ICdCYWNpbGxpJywgJ0FidW5kYW5jZSddLCBpY1tpYyRDbGFzcyA9PSAnQmFjaWxsaScsICdBYnVuZGFuY2UnXSwNCiAgICAgICAgICAgICAgICAgICAgIGNjW2NjJENsYXNzID09ICdCYWNpbGxpJywgJ0FidW5kYW5jZSddKQ0KbGFwcGx5KGJhY2lsbGkubGlzdCwgbWVkaWFuKQ0Ka3J1c2thbC50ZXN0KGJhY2lsbGkubGlzdCkNCg0Kcm0oYmFjaWxsaS5saXN0LCBrdy5wdmFscywgbWVhbnMsIHBsb3QsIHBzbS5saXN0LmNsYXNzLCBzaWdzLCB0b3BOLCB0b3BOLnRheGEsDQogICBzYywgaWMsIGNjLCBpKQ0KDQpgYGANCg0KDQojIyBGaWd1cmVzIFM1IGFuZCBTNi4gSGVhbHRoeSB2cyBORUMgKGFsbCBmZWQpIC0gY2xhc3MgbGV2ZWwNCg0KYGBge3J9DQojIHRvcCBOIG51bWJlciBvZiB0YXhhIHRvIGNvbXBhcmUNCnRvcE4gPC0gNA0KDQojIHByZXBhcmUgdG8gcGxvdCB0b3AgTiAiQ0xBU1MiIGluIGJveHBsb3RzDQpwc20ubGlzdC5jbGFzcyA8LSBsYXBwbHkocHMubGlzdC5jbGFzcywgcHNtZWx0KQ0KDQojIGdldCB0b3AgTiB0YXhhIHJhbmtlZCBieSBkZWNyZWFzaW5nIG1lYW4gYWJ1bmRhbmNlDQptZWFucyA8LSBsYXBwbHkocHNtLmxpc3QuY2xhc3MsIGZ1bmN0aW9uKHgpeyBkZHBseSh4LCB+IENsYXNzLA0KICAgICAgICAgICAgICAgICAgICAgICAgICAgICAgICAgICAgICAgICAgICAgICAgICAgZnVuY3Rpb24oeSl7IGMobWVhbiA9IG1lYW4oeSRBYnVuZGFuY2UpKSB9KSB9KQ0KbWVhbnMgPC0gbGFwcGx5KG1lYW5zLCBmdW5jdGlvbih4KXsgeFtyZXYob3JkZXIoeCRtZWFuKSksIF0gfSkNCnRvcE4udGF4YSA8LSBsYXBwbHkobWVhbnMsIGZ1bmN0aW9uKHgpeyB4WzE6dG9wTiwgJ0NsYXNzJ10gfSkNCnRvcE4udGF4YSA8LSBsYXBwbHkodG9wTi50YXhhLCBmdW5jdGlvbih4KSB7IHRvcE4udGF4YVtbJ0NvbG9uIENvbnRlbnRzJ11dIH0pICMgZm9yY2Ugc2FtZSBvcmRlcg0KcHNtLmxpc3QuY2xhc3MgPC0gbWFwcGx5KGZ1bmN0aW9uKHgsIGkpeyB4W3gkQ2xhc3MgJWluJSB0b3BOLnRheGFbW2ldXSwgXSB9LCBwc20ubGlzdC5jbGFzcywgc2VxX2Fsb25nKHBzbS5saXN0LmNsYXNzKSwNCiAgICAgICAgICAgICAgICAgICAgICAgICBTSU1QTElGWSA9IEZBTFNFLCBVU0UuTkFNRVMgPSBUUlVFKQ0KDQojIGRldGVybWluZSBpZiBhbnkgZGlmZmVyZW5jZXMgYWNyb3NzIGdyb3Vwcw0KbXd1LnB2YWxzIDwtIGxhcHBseShwc20ubGlzdC5jbGFzcywgZnVuY3Rpb24oeCl7IGRkcGx5KHgsIH4gQ2xhc3MsIGZ1bmN0aW9uKHkpIHsNCiAgYyhwcm9iID0gd2lsY294LnRlc3QoZGF0YSA9IHksIEFidW5kYW5jZSB+IGhpc3QubmVjLCBleGFjdCA9IEZBTFNFKSRwLnZhbHVlKSB9KSB9KQ0KZm9yKGkgaW4gc2VxX2Fsb25nKG13dS5wdmFscykpIHsNCiAgbXd1LnB2YWxzW1tpXV0kcHJvYiA8LSBwLmFkanVzdChtd3UucHZhbHNbW2ldXSRwcm9iLCAnaG9sbScpDQp9DQojIGdldCBsYWJlbHMgZm9yIHRheGEgd2hpY2ggYXJlIGRpZmZlcmVudA0Kc2lncyA8LSBsbHBseShuYW1lcyhtd3UucHZhbHMpLCBmdW5jdGlvbih4KSB7bXd1LnB2YWxzW1t4XV0kcHJvYiA8PSAwLjA1fSkNCm5hbWVzKHNpZ3MpIDwtIG5hbWVzKG13dS5wdmFscykNCnNpZ3MgPC0gbGxwbHkobmFtZXMoc2lncyksIGZ1bmN0aW9uKHgpIHsgaWYoYW55KHNpZ3NbW3hdXSkpIG13dS5wdmFsc1tbeF1dW3NpZ3NbW3hdXSwgJ0NsYXNzJ10gZWxzZSBOQSB9KQ0KbmFtZXMoc2lncykgPC0gbmFtZXMobXd1LnB2YWxzKQ0KDQpmb3IoaSBpbiBzZXFfYWxvbmcocHNtLmxpc3QuY2xhc3MpKSB7DQogIHBsb3QgPC0gZ2dwbG90KGRhdGEuZnJhbWUocHNtLmxpc3QuY2xhc3NbW2ldXSksIGFlcyh4ID0gQ2xhc3MsIHkgPSBBYnVuZGFuY2UsIGNvbG9yID0gaGlzdC5uZWMpKSArIA0KICAgIGdlb21fYm94cGxvdChsd2QgPSAxLCBwb3NpdGlvbiA9IHBvc2l0aW9uX2RvZGdlKHdpZHRoID0gMC43NSksIHdpZHRoID0gMC43LA0KICAgICAgICAgICAgICAgICBvdXRsaWVyLnNoYXBlID0gTkEpICsNCiAgICBnZW9tX3BvaW50KHNoYXBlID0gMTksIHBvc2l0aW9uID0gcG9zaXRpb25faml0dGVyZG9kZ2Uoaml0dGVyLndpZHRoID0gMC4xNSwNCiAgICAgICAgICAgICAgICAgICAgICAgICAgICAgICAgICAgICAgICAgICAgICAgICAgICAgICAgICAgZG9kZ2Uud2lkdGggPSAwLjc1KSkgKw0KICAgIHNjYWxlX3hfZGlzY3JldGUobGltaXRzID0gdW5saXN0KHRvcE4udGF4YVtpXSkpICsNCiAgICBzY2FsZV95X3NxcnQoYnJlYWtzID0gYygxLCAwLjY0LCAwLjM2LCAwLjE2LCAwLjA0LCAwLjApLCBsaW1pdHMgPSBjKDAsIDEuMSksIGxhYmVscyA9IHBlcmNlbnQpICsNCiAgICBzY2FsZV9jb2xvdXJfbWFudWFsKHZhbHVlcyA9IGhuX2NvbG9ycykgKyANCiAgICBhbm5vdGF0ZSgndGV4dCcsIHggPSBzaWdzW1tpXV0sIG5hLnJtID0gVFJVRSwgeSA9IDEuMDUsIGxhYmVsID0gJyonLCBzaXplID0gMTIpICsNCiAgICB0aGVtZShheGlzLnRleHQueCA9IGVsZW1lbnRfdGV4dChhbmdsZSA9IC00NSwgaGp1c3QgPSAwLCB2anVzdCA9IDEpKSArDQogICAgbGFicyhsaXN0KHRpdGxlID0gbmFtZXMocHNtLmxpc3QuY2xhc3MpW2ldLCB4ID0gZWxlbWVudF9ibGFuaygpLCB5ID0gZWxlbWVudF90ZXh0KCdSZWxhdGl2ZSBBYnVuZGFuY2UnKSkpICsNCiAgICB0aGVtZShsZWdlbmQucG9zaXRpb24gPSAncmlnaHQnLCBsZWdlbmQudGl0bGUgPSBlbGVtZW50X2JsYW5rKCksIHBsb3QubWFyZ2luID0gbWFyZ2luKDEwLCAxMCwgMTAsIDI1KSkNCiAgcHJpbnQocGxvdCkNCn0NCg0Kcm0obWVhbnMsIG13dS5wdmFscywgcGxvdCwgcHNtLmxpc3QuY2xhc3MsIHNpZ3MsIHRvcE4sIHRvcE4udGF4YSwgaSkNCg0KYGBgDQoNCg0KIyMgRmlndXJlcyAzIGFuZCBTNy4gRGlldCBncm91cHMgKGFsbCBmZWQpIC0gZ2VudXMgbGV2ZWwNCg0KYGBge3J9DQojIHRvcCBOIG51bWJlciBvZiB0YXhhIHRvIGNvbXBhcmUNCnRvcE4gPC0gNw0KDQojIGFnZ2xvbWVyYXRlIE9UVXMgYnkgZ2VudXMNCnBzLmxpc3QuZ2VudXMgPC0gbGxwbHkocHMubGlzdCwgZnVuY3Rpb24oeCkgeyB0YXhfZ2xvbSh4LCAnR2VudXMnKSB9KQ0KDQojIG1lbHQgZGF0YSB0byBsb25nIGZvcm1hdA0KcHNtLmxpc3QuZ2VudXMgPC0gbGxwbHkocHMubGlzdC5nZW51cywgZnVuY3Rpb24oeCkgcHNtZWx0KHgpKQ0KDQojIGdldCB0b3AgTiB0YXhhIHJhbmtlZCBieSBkZWNyZWFzaW5nIG1lZGlhbiBhYnVuZGFuY2UNCm1lZGlhbnMgPC0gbGFwcGx5KHBzbS5saXN0LmdlbnVzLCBmdW5jdGlvbih4KXsgZGRwbHkoeCwgfiBHZW51cywNCiAgICAgICAgICAgICAgICAgICAgICAgICAgICAgICAgICAgICAgICAgICAgICAgICAgICAgZnVuY3Rpb24oeSl7IGMobWVkaWFuID0gbWVkaWFuKHkkQWJ1bmRhbmNlKSkgfSkgfSkNCm1lZGlhbnMgPC0gbGFwcGx5KG1lZGlhbnMsIGZ1bmN0aW9uKHgpeyB4W3JldihvcmRlcih4JG1lZGlhbikpLCBdIH0pDQp0b3BOLnRheGEgPC0gbGFwcGx5KG1lZGlhbnMsIGZ1bmN0aW9uKHgpeyB4WzE6dG9wTiwgJ0dlbnVzJ10gfSkNCnRvcE4udGF4YSA8LSBsYXBwbHkodG9wTi50YXhhLCBmdW5jdGlvbih4KSB7IHRvcE4udGF4YVtbJ0NvbG9uIENvbnRlbnRzJ11dIH0pICMgZm9yY2Ugc2FtZSBvcmRlcg0KcHNtLmxpc3QuZ2VudXMgPC0gbWFwcGx5KGZ1bmN0aW9uKHgsIGkpeyB4W3gkR2VudXMgJWluJSB0b3BOLnRheGFbW2ldXSwgXSB9LCBwc20ubGlzdC5nZW51cywgc2VxX2Fsb25nKHBzbS5saXN0LmdlbnVzKSwNCiAgICAgICAgICAgICAgICAgICAgICAgICBTSU1QTElGWSA9IEZBTFNFLCBVU0UuTkFNRVMgPSBUUlVFKQ0KDQojIGRldGVybWluZSBpZiBhbnkgZGlmZmVyZW5jZXMgYWNyb3NzIGdyb3Vwcw0Ka3cucHZhbHMgPC0gbGFwcGx5KHBzbS5saXN0LmdlbnVzLCBmdW5jdGlvbih4KXsgZGRwbHkoeCwgfiBHZW51cywgZnVuY3Rpb24oeSkgew0KICBjKHByb2IgPSBrcnVza2FsLnRlc3QoZGF0YSA9IHksIEFidW5kYW5jZSB+IGdyb3VwKSRwLnZhbHVlKSB9KSB9KQ0KZm9yKGkgaW4gc2VxX2Fsb25nKGt3LnB2YWxzKSkgew0KICBrdy5wdmFsc1tbaV1dJHByb2IgPC0gcC5hZGp1c3Qoa3cucHZhbHNbW2ldXSRwcm9iLCAnaG9sbScpDQp9DQojIGdldCBsYWJlbHMgZm9yIHRheGEgd2hpY2ggYXJlIGRpZmZlcmVudA0Kc2lncyA8LSBsbHBseShuYW1lcyhrdy5wdmFscyksIGZ1bmN0aW9uKHgpIHtrdy5wdmFsc1tbeF1dJHByb2IgPD0gMC4wNX0pDQpuYW1lcyhzaWdzKSA8LSBuYW1lcyhrdy5wdmFscykNCnNpZ3MgPC0gbGxwbHkobmFtZXMoc2lncyksIGZ1bmN0aW9uKHgpIHsgaWYoYW55KHNpZ3NbW3hdXSkpIGt3LnB2YWxzW1t4XV1bc2lnc1tbeF1dLCAnR2VudXMnXSBlbHNlIE5BIH0pDQpuYW1lcyhzaWdzKSA8LSBuYW1lcyhrdy5wdmFscykNCg0KIyBtYWtlIGJveHBsb3RzDQpmb3IoaSBpbiBzZXFfYWxvbmcocHNtLmxpc3QuZ2VudXMpKSB7DQogIHBsb3QgPC0gZ2dwbG90KGRhdGEuZnJhbWUocHNtLmxpc3QuZ2VudXNbW2ldXSksIGFlcyh4ID0gR2VudXMsIHkgPSBBYnVuZGFuY2UsIGNvbG9yID0gZ3JvdXApKSArIA0KICAgIGdlb21fYm94cGxvdChsd2QgPSAxLCBwb3NpdGlvbiA9IHBvc2l0aW9uX2RvZGdlKHdpZHRoID0gMC43NSksIHdpZHRoID0gMC43LA0KICAgICAgICAgICAgICAgICBvdXRsaWVyLnNoYXBlID0gTkEpICsNCiAgICBnZW9tX3BvaW50KHNoYXBlID0gMTksIHBvc2l0aW9uID0gcG9zaXRpb25faml0dGVyZG9kZ2Uoaml0dGVyLndpZHRoID0gMC4xNSwNCiAgICAgICAgICAgICAgICAgICAgICAgICAgICAgICAgICAgICAgICAgICAgICAgICAgICAgICAgICAgZG9kZ2Uud2lkdGggPSAwLjc1KSkgKw0KICAgIHNjYWxlX3hfZGlzY3JldGUobGltaXRzID0gdG9wTi50YXhhW1tpXV0pICsNCiAgICBzY2FsZV95X3NxcnQoYnJlYWtzID0gYygxLCAwLjY0LCAwLjM2LCAwLjE2LCAwLjA0LCAwLjApLCBsaW1pdHMgPSBjKDAsIDEuMSksIGxhYmVscyA9IHBlcmNlbnQpICsNCiAgICBzY2FsZV9jb2xvdXJfbWFudWFsKHZhbHVlcyA9IGRpZXRfY29sb3JzKSArIA0KICAgIGFubm90YXRlKCd0ZXh0JywgeCA9IHVubGlzdChzaWdzW2ldKSwgbmEucm0gPSBUUlVFLCB5ID0gMS4wNSwgbGFiZWwgPSAnKicsIHNpemUgPSAxMikgKw0KICAgIHRoZW1lKGF4aXMudGV4dC54ID0gZWxlbWVudF90ZXh0KGFuZ2xlID0gLTQ1LCBoanVzdCA9IDAsIHZqdXN0ID0gMSkpICsNCiAgICBsYWJzKGxpc3QodGl0bGUgPSBuYW1lcyhwc20ubGlzdC5nZW51cylbaV0sIHggPSBlbGVtZW50X2JsYW5rKCksIHkgPSBlbGVtZW50X3RleHQoJ1JlbGF0aXZlIEFidW5kYW5jZScpKSkgKw0KICAgIHRoZW1lKGxlZ2VuZC5wb3NpdGlvbiA9ICdyaWdodCcsIGxlZ2VuZC50aXRsZSA9IGVsZW1lbnRfYmxhbmsoKSwgcGxvdC5tYXJnaW4gPSBtYXJnaW4oMTAsIDEwLCAxMCwgMjUpKQ0KICBwcmludChwbG90KQ0KfQ0KDQojIGZpbmQgbWVkaWFuIHJlbGF0aXZlIGFidW5kYW5jZSBvZiBFbnRlcm9iYWN0ZXJpYWNlYWUNCmljIDwtIHBzbS5saXN0LmdlbnVzJCdJbGV1bSBDb250ZW50cycNCmNjIDwtIHBzbS5saXN0LmdlbnVzJCdDb2xvbiBDb250ZW50cycNCmVudC5saXN0IDwtIGxpc3QoaWNbaWMkR2VudXMgPT0gJ0VudGVyb2JhY3RlcmlhY2VhZSB1bmNsYXNzaWZpZWQnLCAnQWJ1bmRhbmNlJ10sDQogICAgICAgICAgICAgICAgIGNjW2NjJEdlbnVzID09ICdFbnRlcm9iYWN0ZXJpYWNlYWUgdW5jbGFzc2lmaWVkJywgJ0FidW5kYW5jZSddKQ0KbWVkaWFuKHVubGlzdChlbnQubGlzdCkpDQoNCnJtKGVudC5saXN0LCBpLCBrdy5wdmFscywgbWVkaWFucywgcGxvdCwgcHNtLmxpc3QuZ2VudXMsIHNpZ3MsIHRvcE4sIHRvcE4udGF4YSwgaWMsIGNjKQ0KDQpgYGANCg0KIyMgRmlndXJlcyA0IGFuZCBTOC4gSGVhbHRoeSB2cyBORUMgKGFsbCBmZWQpIC0gR2VudXMgbGV2ZWwNCg0KYGBge3J9DQojIHRvcCBOIG51bWJlciBvZiB0YXhhIHRvIGNvbXBhcmUNCnRvcE4gPC0gNw0KDQojIHByZXBhcmUgdG8gcGxvdCB0b3AgTiAiR0VOVVMiIGluIGJveHBsb3RzDQpwc20ubGlzdC5nZW51cyA8LSBsYXBwbHkocHMubGlzdC5nZW51cywgcHNtZWx0KQ0KDQojIGdldCB0b3AgTiB0YXhhIHJhbmtlZCBieSBkZWNyZWFzaW5nIG1lZGlhbiBhYnVuZGFuY2UNCm1lZGlhbnMgPC0gbGFwcGx5KHBzbS5saXN0LmdlbnVzLCBmdW5jdGlvbih4KXsgZGRwbHkoeCwgfiBHZW51cywNCiAgICAgICAgICAgICAgICAgICAgICAgICAgICAgICAgICAgICAgICAgICAgICAgICAgICAgZnVuY3Rpb24oeSl7IGMobWVkaWFuID0gbWVkaWFuKHkkQWJ1bmRhbmNlKSkgfSkgfSkNCm1lZGlhbnMgPC0gbGFwcGx5KG1lZGlhbnMsIGZ1bmN0aW9uKHgpeyB4W3JldihvcmRlcih4JG1lZGlhbikpLCBdIH0pDQp0b3BOLnRheGEgPC0gbGFwcGx5KG1lZGlhbnMsIGZ1bmN0aW9uKHgpeyB4WzE6dG9wTiwgJ0dlbnVzJ10gfSkNCnRvcE4udGF4YSA8LSBsYXBwbHkodG9wTi50YXhhLCBmdW5jdGlvbih4KSB7IHRvcE4udGF4YVtbJ0NvbG9uIENvbnRlbnRzJ11dIH0pICMgZm9yY2Ugc2FtZSBvcmRlcg0KcHNtLmxpc3QuZ2VudXMgPC0gbWFwcGx5KGZ1bmN0aW9uKHgsIGkpeyB4W3gkR2VudXMgJWluJSB0b3BOLnRheGFbW2ldXSwgXSB9LCBwc20ubGlzdC5nZW51cywgc2VxX2Fsb25nKHBzbS5saXN0LmdlbnVzKSwNCiAgICAgICAgICAgICAgICAgICAgICAgICBTSU1QTElGWSA9IEZBTFNFLCBVU0UuTkFNRVMgPSBUUlVFKQ0KDQojIGRldGVybWluZSBpZiBhbnkgZGlmZmVyZW5jZXMgYWNyb3NzIGdyb3Vwcw0KbXd1LnB2YWxzIDwtIGxhcHBseShwc20ubGlzdC5nZW51cywgZnVuY3Rpb24oeCl7IGRkcGx5KHgsIH4gR2VudXMsIGZ1bmN0aW9uKHkpIHsNCiAgYyhwcm9iID0gd2lsY294LnRlc3QoZGF0YSA9IHksIEFidW5kYW5jZSB+IGhpc3QubmVjLCBleGFjdCA9IEZBTFNFKSRwLnZhbHVlKSB9KSB9KQ0KZm9yKGkgaW4gc2VxX2Fsb25nKG13dS5wdmFscykpIHsNCiAgbXd1LnB2YWxzW1tpXV0kcHJvYiA8LSBwLmFkanVzdChtd3UucHZhbHNbW2ldXSRwcm9iLCAnaG9sbScpDQp9DQojIGdldCBsYWJlbHMgZm9yIHRheGEgd2hpY2ggYXJlIGRpZmZlcmVudA0Kc2lncyA8LSBsbHBseShuYW1lcyhtd3UucHZhbHMpLCBmdW5jdGlvbih4KSB7bXd1LnB2YWxzW1t4XV0kcHJvYiA8PSAwLjA1fSkNCm5hbWVzKHNpZ3MpIDwtIG5hbWVzKG13dS5wdmFscykNCnNpZ3MgPC0gbGxwbHkobmFtZXMoc2lncyksIGZ1bmN0aW9uKHgpIHsgaWYoYW55KHNpZ3NbW3hdXSkpIG13dS5wdmFsc1tbeF1dW3NpZ3NbW3hdXSwgJ0dlbnVzJ10gZWxzZSBOQSB9KQ0KbmFtZXMoc2lncykgPC0gbmFtZXMobXd1LnB2YWxzKQ0KDQpmb3IoaSBpbiBzZXFfYWxvbmcocHNtLmxpc3QuZ2VudXMpKSB7DQogIHBsb3QgPC0gZ2dwbG90KGRhdGEuZnJhbWUocHNtLmxpc3QuZ2VudXNbW2ldXSksIGFlcyh4ID0gR2VudXMsIHkgPSBBYnVuZGFuY2UsIGNvbG9yID0gaGlzdC5uZWMpKSArIA0KICAgIGdlb21fYm94cGxvdChsd2QgPSAxLCBwb3NpdGlvbiA9IHBvc2l0aW9uX2RvZGdlKHdpZHRoID0gMC43NSksIHdpZHRoID0gMC43LA0KICAgICAgICAgICAgICAgICBvdXRsaWVyLnNoYXBlID0gTkEpICsNCiAgICBnZW9tX3BvaW50KHNoYXBlID0gMTksIHBvc2l0aW9uID0gcG9zaXRpb25faml0dGVyZG9kZ2Uoaml0dGVyLndpZHRoID0gMC4xNSwNCiAgICAgICAgICAgICAgICAgICAgICAgICAgICAgICAgICAgICAgICAgICAgICAgICAgICAgICAgICAgZG9kZ2Uud2lkdGggPSAwLjc1KSkgKw0KICAgIHNjYWxlX3hfZGlzY3JldGUobGltaXRzID0gdW5saXN0KHRvcE4udGF4YVtpXSkpICsNCiAgICBzY2FsZV95X3NxcnQoYnJlYWtzID0gYygxLCAwLjY0LCAwLjM2LCAwLjE2LCAwLjA0LCAwLjApLCBsaW1pdHMgPSBjKDAsIDEuMSksIGxhYmVscyA9IHBlcmNlbnQpICsNCiAgICBzY2FsZV9jb2xvdXJfbWFudWFsKHZhbHVlcyA9IGhuX2NvbG9ycykgKyANCiAgICBhbm5vdGF0ZSgndGV4dCcsIHggPSBzaWdzW1tpXV0sIG5hLnJtID0gVFJVRSwgeSA9IDEuMDUsIGxhYmVsID0gJyonLCBzaXplID0gMTIpICsNCiAgICB0aGVtZShheGlzLnRleHQueCA9IGVsZW1lbnRfdGV4dChhbmdsZSA9IC00NSwgaGp1c3QgPSAwLCB2anVzdCA9IDEpKSArDQogICAgbGFicyhsaXN0KHRpdGxlID0gbmFtZXMocHNtLmxpc3QuZ2VudXMpW2ldLCB4ID0gZWxlbWVudF9ibGFuaygpLCB5ID0gZWxlbWVudF90ZXh0KCdSZWxhdGl2ZSBBYnVuZGFuY2UnKSkpICsNCiAgICB0aGVtZShsZWdlbmQucG9zaXRpb24gPSAncmlnaHQnLCBsZWdlbmQudGl0bGUgPSBlbGVtZW50X2JsYW5rKCksIHBsb3QubWFyZ2luID0gbWFyZ2luKDEwLCAxMCwgMTAsIDI1KSkNCiAgcHJpbnQocGxvdCkNCn0NCg0KIyBtZWFzdXJlIGRpZmZlcmVuY2UgaW4gQ2xvc3RyaWRpdW0gc2Vuc3Ugc3RyaWN0byBiZXR3ZWVuIGhlYWx0aHkvTkVDDQppYyA8LSBwc20ubGlzdC5nZW51cyQnSWxldW0gQ29udGVudHMnDQp3aXRoKGljW2ljJEdlbnVzID09ICdDbG9zdHJpZGl1bSBzZW5zdSBzdHJpY3RvJywgYygnQWJ1bmRhbmNlJywgJ2hpc3QubmVjJyldLCANCiAgICAgYWdncmVnYXRlKEFidW5kYW5jZSB+IGhpc3QubmVjLCBGVU49bWVkaWFuKSkNCg0KIyBtZWFzdXJlIGRpZmZlcmVuY2UgaW4gRW50ZXJvYmFjdGVyaWFjZWFlIGJldHdlZW4gaGVhbHRoeS9ORUMNCnNjIDwtIHBzbS5saXN0LmdlbnVzJCdTdG9tYWNoIENvbnRlbnRzJw0Kd2l0aChzY1tzYyRHZW51cyA9PSAnRW50ZXJvYmFjdGVyaWFjZWFlIHVuY2xhc3NpZmllZCcsIGMoJ0FidW5kYW5jZScsICdoaXN0Lm5lYycpXSwNCiAgICAgYWdncmVnYXRlKEFidW5kYW5jZSB+IGhpc3QubmVjLCBGVU49bWVkaWFuKSkNCndpdGgoaWNbaWMkR2VudXMgPT0gJ0VudGVyb2JhY3RlcmlhY2VhZSB1bmNsYXNzaWZpZWQnLCBjKCdBYnVuZGFuY2UnLCAnaGlzdC5uZWMnKV0sDQogICAgIGFnZ3JlZ2F0ZShBYnVuZGFuY2UgfiBoaXN0Lm5lYywgRlVOPW1lZGlhbikpDQoNCnJtKGksIG1lZGlhbnMsIG13dS5wdmFscywgcGxvdCwgcHNtLmxpc3QuZ2VudXMsIHNpZ3MsIHRvcE4sIHRvcE4udGF4YSwgaWMpDQoNCmBgYA0KDQojIyBNZXRhYm9saXRlcyBvdmVydmlldyAoYWxsIGZlZCkNCg0KYGBge3J9DQojIGxvYWQgZGF0YQ0KYy5jbXBkcyA8LSByZWFkLmNzdignY2VjYWxfY29tcG91bmRzLmNzdicsIHN0cmlwLndoaXRlID0gVCkNCmNlY2FsIDwtIHJlYWQuY3N2KCdjZWNhbC5jc3YnLCBzdHJpcC53aGl0ZSA9IFQpICAjICJDT01QIElEIiB1bmlxdWVseSBpZGVudGlmaWVzIGNvbXBvdW5kcw0KcC5jbXBkcyA8LSByZWFkLmNzdigncGxhc21hX2NvbXBvdW5kcy5jc3YnLCBzdHJpcC53aGl0ZSA9IFQpDQpwbGFzbWEgPC0gcmVhZC5jc3YoJ3BsYXNtYS5jc3YnLCBzdHJpcC53aGl0ZSA9IFQpDQpwaWdzIDwtIGdldE1KTlBpZ3MoaW5jbC5uZXcgPSBGKQ0KY2VjYWwgPC0gbWVyZ2UocGlncywgY2VjYWwpDQpwbGFzbWEgPC0gbWVyZ2UocGlncywgcGxhc21hKQ0KDQojIGdldCBjb2x1bW4gbmFtZXMgZm9yIGNvbXBvdW5kcw0KYy5jb21wSURzIDwtIG5hbWVzKGNlY2FsKVshKG5hbWVzKGNlY2FsKSAlaW4lIGMobmFtZXMocGlncyksICdtYXNzLmRyeS5nJywgJ2JyYWRmb3JkLnByb3RlaW4nKSldDQpwLmNvbXBJRHMgPC0gbmFtZXMocGxhc21hKVshKG5hbWVzKHBsYXNtYSkgJWluJSBuYW1lcyhwaWdzKSldDQoNCiMgcmVtb3ZlIGNvbXBvdW5kcyB0aGF0IGhhdmUgbm8gdmFsdWVzIChpLmUuIHdlcmUgb25seSBmb3VuZCBpbiAnTkVXJyBwaWdzKQ0KIyAgY2VjYWwNCmMuY29tcElEcy50b0ZpbHRlciA8LSBuYW1lcyh3aGljaChjb2xTdW1zKGNlY2FsWywgYy5jb21wSURzXSwgbmEucm0gPSBUKSA9PSAwKSkNCmNlY2FsIDwtIGNlY2FsWyFuYW1lcyhjZWNhbCkgJWluJSBjLmNvbXBJRHMudG9GaWx0ZXJdDQpjLmNvbXBJRHMgPC0gc2V0ZGlmZihjLmNvbXBJRHMsIGMuY29tcElEcy50b0ZpbHRlcikNCiMgIHBsYXNtYQ0KcC5jb21wSURzLnRvRmlsdGVyIDwtIG5hbWVzKHdoaWNoKGNvbFN1bXMocGxhc21hWywgcC5jb21wSURzXSwgbmEucm0gPSBUKSA9PSAwKSkNCnBsYXNtYSA8LSBwbGFzbWFbIW5hbWVzKHBsYXNtYSkgJWluJSBwLmNvbXBJRHMudG9GaWx0ZXJdDQpwLmNvbXBJRHMgPC0gc2V0ZGlmZihwLmNvbXBJRHMsIHAuY29tcElEcy50b0ZpbHRlcikNCg0KIyBub3JtYWxpemUgY29uY2VudHJhdGlvbiBtZWFzdXJlbWVudHMgZm9yIGVhY2ggY2VjYWwgc2FtcGxlIC0gZGl2aWRlIGJ5IGRyeSBtYXNzIG9mIHNhbXBsZQ0KY2VjYWxbLCBjLmNvbXBJRHNdIDwtIHN3ZWVwKGNlY2FsWywgYy5jb21wSURzXSwgMSwgY2VjYWxbLCAnbWFzcy5kcnkuZyddLCAnLycpDQoNCiMgcmVwbGFjZSAwcyBhbmQgTkEgKG1pc3NpbmcgdmFsdWVzKSB3LyAxLzIgY29sdW1uIG1pbmltdW0NCiMgIGNlY2FsDQpjLmNvbG1pbnMgPC0gYXBwbHkoY2VjYWxbLCBjLmNvbXBJRHNdLCAyLCBmdW5jdGlvbih4KXsgbWluKHgsIG5hLnJtID0gVCkgfSkNCmMubmFzIDwtIHdoaWNoKGlzLm5hKGNlY2FsKSwgYXJyLmluZCA9IFQpDQpjLm5hcyA8LSBjLm5hc1t3aGljaChjLm5hc1ssIDJdID4gbmNvbChjZWNhbCkgLSBsZW5ndGgoYy5jb21wSURzKSksIF0NCmNlY2FsW2MubmFzXSA8LSBjLmNvbG1pbnNbYy5uYXNbLCAyXSAtIChuY29sKGNlY2FsKSAtIGxlbmd0aChjLmNvbXBJRHMpKV0gLyAyDQojICBwbGFzbWENCnAuY29sbWlucyA8LSBhcHBseShwbGFzbWFbLCBwLmNvbXBJRHNdLCAyLCBmdW5jdGlvbih4KXsgbWluKHgsIG5hLnJtID0gVCkgfSkNCnAubmFzIDwtIHdoaWNoKGlzLm5hKHBsYXNtYSksIGFyci5pbmQgPSBUKQ0KcC5uYXMgPC0gcC5uYXNbd2hpY2gocC5uYXNbLCAyXSA+IG5jb2wocGxhc21hKSAtIGxlbmd0aChwLmNvbXBJRHMpKSwgXQ0KcGxhc21hW3AubmFzXSA8LSBwLmNvbG1pbnNbcC5uYXNbLCAyXSAtIChuY29sKHBsYXNtYSkgLSBsZW5ndGgocC5jb21wSURzKSldIC8gMg0KDQojIHNhdmUgYWJzb2x1dGUgdmFsdWVzIHRvIHVzZSBmb3IgY2FsY3VsYXRpbmcgZm9sZC1jaGFuZ2UNCmMuYWJzIDwtIGNlY2FsDQpwLmFicyA8LSBwbGFzbWENCg0KIyB0cmFuc2Zvcm0gdmFsdWVzIGJ5IGdlbmVyYWxpemVkIGxvZw0KY2VjYWxbLCBjLmNvbXBJRHNdIDwtIGFwcGx5KGNlY2FsWywgYy5jb21wSURzXSwgMiwgZnVuY3Rpb24oeCkgeyBsb2cyKCh4ICsgc3FydCh4XjIgKyAxKSkgLyAyKSB9KQ0KcGxhc21hWywgcC5jb21wSURzXSA8LSBhcHBseShwbGFzbWFbLCBwLmNvbXBJRHNdLCAyLCBmdW5jdGlvbih4KSB7IGxvZzIoKHggKyBzcXJ0KHheMiArIDEpKSAvIDIpIH0pDQoNCiMgc2NhbGUgdmFsdWVzIGJ5IGF1dG9zY2FsaW5nIChmb3JjZSBlYWNoIHZhcmlhYmxlIHRvIGhhdmUgbWVhbiA9IDAgYW5kIHNkID0gMSkNCmNlY2FsWywgYy5jb21wSURzXSA8LSBhcHBseShjZWNhbFssIGMuY29tcElEc10sIDIsIGZ1bmN0aW9uKHgpIHsgKHggLSBtZWFuKHgpKSAvIHNkKHgpIH0pDQpwbGFzbWFbLCBwLmNvbXBJRHNdIDwtIGFwcGx5KHBsYXNtYVssIHAuY29tcElEc10sIDIsIGZ1bmN0aW9uKHgpIHsgKHggLSBtZWFuKHgpKSAvIHNkKHgpIH0pDQoNCiMgY2hhbmdlIGNvbHVtbiBuYW1lcyBmcm9tICdjLiMjIyMjJyBvciAncC4jIyMjIycgZm9ybWF0IHRvIGJpb2NoZW1pY2FsIG5hbWUNCiMgIGNlY2FsDQpjLmNtcGRzJGNvbG5hbWVzSUQgPC0gcGFzdGUoJ2MuJywgYy5jbXBkcyRjb21wLmlkLCBzZXA9JycpDQpjLmNvbXAubmFtZXMgPC0gYXMuY2hhcmFjdGVyKGMuY21wZHMkYmlvY2hlbWljYWxbbmEub21pdChtYXRjaChjb2xuYW1lcyhjZWNhbCksIGMuY21wZHMkY29sbmFtZXNJRCkpXSkNCmNvbG5hbWVzKGNlY2FsKVtjb2xuYW1lcyhjZWNhbCkgJWluJSBjLmNvbXBJRHNdIDwtIGMuY29tcC5uYW1lcw0KY29sbmFtZXMoYy5hYnMpW2NvbG5hbWVzKGMuYWJzKSAlaW4lIGMuY29tcElEc10gPC0gYy5jb21wLm5hbWVzDQojIHBsYXNtYQ0KcC5jbXBkcyRjb2xuYW1lc0lEIDwtIHBhc3RlKCdwLicsIHAuY21wZHMkY29tcC5pZCwgc2VwPScnKQ0KcC5jb21wLm5hbWVzIDwtIGFzLmNoYXJhY3RlcihwLmNtcGRzJGJpb2NoZW1pY2FsW25hLm9taXQobWF0Y2goY29sbmFtZXMocGxhc21hKSwgcC5jbXBkcyRjb2xuYW1lc0lEKSldKQ0KY29sbmFtZXMocGxhc21hKVtjb2xuYW1lcyhwbGFzbWEpICVpbiUgcC5jb21wSURzXSA8LSBwLmNvbXAubmFtZXMNCmNvbG5hbWVzKHAuYWJzKVtjb2xuYW1lcyhwLmFicykgJWluJSBwLmNvbXBJRHNdIDwtIHAuY29tcC5uYW1lcw0KDQpjLmZlZCA8LSBkcm9wbGV2ZWxzKGNlY2FsW2NlY2FsJGdyb3VwICVpbiUgYygnTEFDJywgJ01JWCcsICdDU1MnKSwgXSkNCnAuZmVkIDwtIGRyb3BsZXZlbHMocGxhc21hW3BsYXNtYSRncm91cCAlaW4lIGMoJ0xBQycsICdNSVgnLCAnQ1NTJyksIF0pDQoNCg0KIyBwbGFzbWENCiMgYnVpbGQgZGF0YWZyYW1lIG9mIGZyb20gdHlwZSBJSSBTUyBBTk9WQSBmb3IgZ3JvdXAgYW5kIGhpc3QubmVjDQoNCiMgdGhpcyBjYWxsIHRvIGFub3ZhIHJldHVybnMgcC12YWxzIGZvciBncm91cCB0ZXJtDQojICAgaS5lLiByZXR1cm4gdGhlIHAtdmFsIGZvciB0aGUgZ3JvdXAgdGVybSBhZnRlciBjb250cm9sbGluZyBmb3IgaGlzdC5uZWMNCnAuYW5vdmEgPC0gYXMuZGF0YS5mcmFtZShhcHBseShwLmZlZFssIGFzLmNoYXJhY3RlcihwLmNvbXAubmFtZXMpXSwgMiwgDQogICAgICAgICAgICAgICAgICAgICAgICAgICAgICAgZnVuY3Rpb24oeCkgYW5vdmEobG0oeCB+IHAuZmVkJGhpc3QubmVjICogcC5mZWQkZ3JvdXApKSQnUHIoPkYpJ1syXSkpDQoNCiMgdGhpcyBjYWxsIHRvIGFub3ZhIHJldHVybnMgcC12YWxzIGZvciBoaXN0Lm5lYyB0ZXJtIGFuZCBncm91cDpoaXN0Lm5lYyBpbnRlcmFjdGlvbiB0ZXJtDQojICAgaS5lLiByZXR1cm4gdGhlIHAtdmFsIGZvciB0aGUgaGlzdC5uZWMgdGVybSBhbmQgZ3JvdXA6aGlzdC5uZWMgaW50ZXJhY3Rpb24gdGVybSANCiMgICBhZnRlciBjb250cm9sbGluZyBmb3IgZ3JvdXANCnAuYW5vdmEgPC0gY2JpbmQocC5hbm92YSwgdChhcHBseShwLmZlZFssIGFzLmNoYXJhY3RlcihwLmNvbXAubmFtZXMpXSwgMiwNCiAgICAgICAgICAgICAgICAgICAgICAgICAgICAgICAgICBmdW5jdGlvbih4KSBhbm92YShsbSh4IH4gcC5mZWQkZ3JvdXAgKiBwLmZlZCRoaXN0Lm5lYykpJCdQcig+RiknWzI6M10pKSkNCmNvbG5hbWVzKHAuYW5vdmEpIDwtIGMoJ2dyb3VwLnAnLCAnaGlzdC5uZWMucCcsICdpdHgucCcpDQoNCiMgYWRkIG1lZGlhbiBmb2xkIGRpZmZlcmVuY2VzIGJldHdlZW4gSGVhbHRoeSBhbmQgTkVDDQptZWRpYW4uaCA8LSBhcHBseShwLmFic1twLmFicyRoaXN0Lm5lYyA9PSAnSGVhbHRoeScsIHJvdy5uYW1lcyhwLmFub3ZhKV0sIDIsIG1lZGlhbiwgbmEucm0gPSBUKQ0KbWVkaWFuLm4gPC0gYXBwbHkocC5hYnNbcC5hYnMkaGlzdC5uZWMgPT0gJ05FQycsIHJvdy5uYW1lcyhwLmFub3ZhKV0sIDIsIG1lZGlhbiwgbmEucm0gPSBUKQ0KcC5hbm92YSRuZWMuZm9sZC5kaWZmIDwtIG1lZGlhbi5oIC8gbWVkaWFuLm4NCg0KIyBhZGQgbWVkaWFuIGZvbGQgZGlmZmVyZW5jZXMgYmV0d2VlbiBMQUMgYW5kIENTUw0KbWVkaWFuLmxhYyA8LSBhcHBseShwLmFic1twLmFicyRncm91cCA9PSAnTEFDJywgcm93Lm5hbWVzKHAuYW5vdmEpXSwgMiwgbWVkaWFuLCBuYS5ybSA9IFQpDQptZWRpYW4uY3NzIDwtIGFwcGx5KHAuYWJzW3AuYWJzJGdyb3VwID09ICdDU1MnLCByb3cubmFtZXMocC5hbm92YSldLCAyLCBtZWRpYW4sIG5hLnJtID0gVCkNCnAuYW5vdmEkZ3JvdXAuZm9sZC5kaWZmIDwtIG1lZGlhbi5sYWMgLyBtZWRpYW4uY3NzDQoNCiMgY29udHJvbCBmYW1pbHktd2lzZSBlcnJvciByYXRlIGZvciBncm91cCBhbmQgaGlzdC5uZWMgdXNpbmcgRkRSDQpwLmFub3ZhWyAsIGMoJ2dyb3VwLnAnLCAnaGlzdC5uZWMucCcsICdpdHgucCcpXSA8LSBhcHBseShwLmFub3ZhWyAsIGMoJ2dyb3VwLnAnLCAnaGlzdC5uZWMucCcsICdpdHgucCcpXSwgMiwgDQogICAgICAgICAgICAgICAgICAgICAgICAgICAgICAgICAgICAgICAgICAgICAgICAgICAgICAgICBmdW5jdGlvbih4KSBwLmFkanVzdCh4LCBtZXRob2QgPSAnZmRyJykpDQoNCiMgY291bnQgaG93IG1hbnkgbWV0YWJvbGl0ZXMgd2VyZSBmb3VuZCB0byBiZSBkaWZmZXJlbnQgYXQgcSA8IDAuMDUNCiMgICBjb3VudCBvbmx5IG1ldGFib2xpdGVzIHNpZ25pZmljYW50IGZvciB0aGUgbWFpbiBlZmZlY3QgQU5EIG5vIGludGVyYWN0aW9uDQpjb3VudChwLmFub3ZhJGdyb3VwLnAgPCAwLjAxICYgcC5hbm92YSRpdHgucCA+IDAuMDEpDQpjb3VudChwLmFub3ZhJGhpc3QubmVjLnAgPCAwLjAxICYgcC5hbm92YSRpdHgucCA+IDAuMDEpDQpjb3VudChwLmFub3ZhJGl0eC5wIDwgMC4wMSkNCg0KDQojIGNlY2FsDQojIGJ1aWxkIGRhdGFmcmFtZSBvZiBmcm9tIHR5cGUgSUkgU1MgQU5PVkEgZm9yIGdyb3VwIGFuZCBoaXN0Lm5lYw0KDQojIHRoaXMgY2FsbCB0byBhbm92YSByZXR1cm5zIHAtdmFscyBmb3IgZ3JvdXAgdGVybQ0KIyAgIGkuZS4gcmV0dXJuIHRoZSBwLXZhbCBmb3IgdGhlIGdyb3VwIHRlcm0gYWZ0ZXIgY29udHJvbGxpbmcgZm9yIGhpc3QubmVjDQpjLmFub3ZhIDwtIGFzLmRhdGEuZnJhbWUoYXBwbHkoYy5mZWRbLCBhcy5jaGFyYWN0ZXIoYy5jb21wLm5hbWVzKV0sIDIsIA0KICAgICAgICAgICAgICAgICAgICAgICAgICAgICAgIGZ1bmN0aW9uKHgpIGFub3ZhKGxtKHggfiBjLmZlZCRoaXN0Lm5lYyAqIGMuZmVkJGdyb3VwKSkkJ1ByKD5GKSdbMl0pKQ0KDQojIHRoaXMgY2FsbCB0byBhbm92YSByZXR1cm5zIHAtdmFscyBmb3IgaGlzdC5uZWMgdGVybSBhbmQgZ3JvdXA6aGlzdC5uZWMgaW50ZXJhY3Rpb24gdGVybQ0KIyAgIGkuZS4gcmV0dXJuIHRoZSBwLXZhbCBmb3IgdGhlIGhpc3QubmVjIHRlcm0gYW5kIGdyb3VwOmhpc3QubmVjIGludGVyYWN0aW9uIHRlcm0gDQojICAgYWZ0ZXIgY29udHJvbGxpbmcgZm9yIGdyb3VwDQpjLmFub3ZhIDwtIGNiaW5kKGMuYW5vdmEsIHQoYXBwbHkoYy5mZWRbLCBhcy5jaGFyYWN0ZXIoYy5jb21wLm5hbWVzKV0sIDIsDQogICAgICAgICAgICAgICAgICAgICAgICAgICAgICAgICAgZnVuY3Rpb24oeCkgYW5vdmEobG0oeCB+IGMuZmVkJGdyb3VwICogYy5mZWQkaGlzdC5uZWMpKSQnUHIoPkYpJ1syOjNdKSkpDQpjb2xuYW1lcyhjLmFub3ZhKSA8LSBjKCdncm91cC5wJywgJ2hpc3QubmVjLnAnLCAnaXR4LnAnKQ0KDQojIGFkZCBtZWRpYW4gZm9sZCBkaWZmZXJlbmNlcyBiZXR3ZWVuIEhlYWx0aHkgYW5kIE5FQw0KbWVkaWFuLmggPC0gYXBwbHkoYy5hYnNbYy5hYnMkaGlzdC5uZWMgPT0gJ0hlYWx0aHknLCByb3cubmFtZXMoYy5hbm92YSldLCAyLCBtZWRpYW4sIG5hLnJtID0gVCkNCm1lZGlhbi5uIDwtIGFwcGx5KGMuYWJzW2MuYWJzJGhpc3QubmVjID09ICdORUMnLCByb3cubmFtZXMoYy5hbm92YSldLCAyLCBtZWRpYW4sIG5hLnJtID0gVCkNCmMuYW5vdmEkbmVjLmZvbGQuZGlmZiA8LSBtZWRpYW4uaCAvIG1lZGlhbi5uDQoNCiMgYWRkIG1lZGlhbiBmb2xkIGRpZmZlcmVuY2VzIGJldHdlZW4gTEFDIGFuZCBDU1MNCm1lZGlhbi5sYWMgPC0gYXBwbHkoYy5hYnNbYy5hYnMkZ3JvdXAgPT0gJ0xBQycsIHJvdy5uYW1lcyhjLmFub3ZhKV0sIDIsIG1lZGlhbiwgbmEucm0gPSBUKQ0KbWVkaWFuLmNzcyA8LSBhcHBseShjLmFic1tjLmFicyRncm91cCA9PSAnQ1NTJywgcm93Lm5hbWVzKGMuYW5vdmEpXSwgMiwgbWVkaWFuLCBuYS5ybSA9IFQpDQpjLmFub3ZhJGdyb3VwLmZvbGQuZGlmZiA8LSBtZWRpYW4ubGFjIC8gbWVkaWFuLmNzcw0KDQojIGNvbnRyb2wgZmFtaWx5LXdpc2UgZXJyb3IgcmF0ZSBmb3IgZ3JvdXAgYW5kIGhpc3QubmVjIHVzaW5nIEZEUg0KYy5hbm92YVsgLCBjKCdncm91cC5wJywgJ2hpc3QubmVjLnAnLCAnaXR4LnAnKV0gPC0gYXBwbHkoYy5hbm92YVsgLCBjKCdncm91cC5wJywgJ2hpc3QubmVjLnAnLCAnaXR4LnAnKV0sIDIsIA0KICAgICAgICAgICAgICAgICAgICAgICAgICAgICAgICAgICAgICAgICAgICAgICAgICAgICAgICAgZnVuY3Rpb24oeCkgcC5hZGp1c3QoeCwgbWV0aG9kID0gJ2ZkcicpKQ0KDQojIGNvdW50IGhvdyBtYW55IG1ldGFib2xpdGVzIHdlcmUgZm91bmQgdG8gYmUgZGlmZmVyZW50IGF0IHEgPCAwLjA1DQojICAgY291bnQgb25seSBtZXRhYm9saXRlcyBzaWduaWZpY2FudCBmb3IgdGhlIG1haW4gZWZmZWN0IEFORCBubyBpbnRlcmFjdGlvbg0KY291bnQoYy5hbm92YSRncm91cC5wIDwgMC4wMSAmIGMuYW5vdmEkaXR4LnAgPiAwLjAxKQ0KY291bnQoYy5hbm92YSRoaXN0Lm5lYy5wIDwgMC4wMSAmIGMuYW5vdmEkaXR4LnAgPiAwLjAxKQ0KY291bnQoYy5hbm92YSRpdHgucCA8IDAuMDEpDQoNCg0Kcm0oYy5hYnMsIHAuYWJzLCBjLmNtcGRzLCBwLmNtcGRzLCBjLmZlZCwgcC5mZWQsIGMubmFzLCBwLm5hcywgcGlncywgbWVkaWFuLmNzcywNCiAgIG1lZGlhbi5sYWMsIG1lZGlhbi5oLCBtZWRpYW4ubiwgcC5jb2xtaW5zLCBjLmNvbG1pbnMsIGMuY29tcC5uYW1lcywgcC5jb21wLm5hbWVzLA0KICAgYy5jb21wSURzLCBwLmNvbXBJRHMsIGMuY29tcElEcy50b0ZpbHRlciwgcC5jb21wSURzLnRvRmlsdGVyKQ0KDQpgYGANCg0KDQpgYGB7cn0NCiMgRmlndXJlIDYuICBDZWNhbCBtZXRhYm9saXRlcyBoZWF0bWFwDQoNCiMgZ2V0IG1ldGFib2xpdGVzIHRvIHVzZSBmb3IgaGVhdG1hcCAtIHVzZSBwLXZhbCBjdXRvZmYgYW5kIHJhbmsgdGhvc2Ugd2l0aGluDQojICAgdGhlIGN1dG9mZiBieSBkZWNyZWFzaW5nIGVmZmVjdCBzaXplLCB0aGVuIHRha2UgdG9wIDUwIChvciBhbGwgaWYgPCA1MCkNCnRtcCA8LSBjLmFub3ZhW3doaWNoKGMuYW5vdmEkaGlzdC5uZWMucCA8IDAuMDUpLCBdDQpjLm1ldGFicy5wbG90IDwtIHJvdy5uYW1lcyh0bXBbb3JkZXIoYWJzKGxvZzIodG1wJG5lYy5mb2xkLmRpZmYpKSwgZGVjcmVhc2luZyA9IFQpLCBdKSAjIGJ5IGVmZmVjdCBzaXplDQpjLm1ldGFicy5wbG90IDwtIGMubWV0YWJzLnBsb3RbMSA6IGlmZWxzZShsZW5ndGgoYy5tZXRhYnMucGxvdCkgPCA1MCwgbGVuZ3RoKGMubWV0YWJzLnBsb3QpLCA1MCldDQpybSh0bXApDQoNCiMgcGxvdCBoZWF0bWFwDQpjLm1hdCA8LSB0KGFzLm1hdHJpeC5kYXRhLmZyYW1lKGNlY2FsW29yZGVyKGNlY2FsJGhpc3QubmVjLCBjZWNhbCRncm91cCksIGMubWV0YWJzLnBsb3RdKSkNCmNvbG5hbWVzKGMubWF0KSA8LSBjZWNhbFtvcmRlcihjZWNhbCRoaXN0Lm5lYywgY2VjYWwkZ3JvdXApLCAncGlnSUQnXQ0Kcm93bmFtZXMoYy5tYXQpIDwtIGMubWV0YWJzLnBsb3QNCmFubm90LmRmIDwtIGNlY2FsWywgYygnaGlzdC5uZWMnLCAnZ3JvdXAnKV0NCmFubm90LmRmWywgJ2hpc3QubmVjJ10gPC0gYXMuY2hhcmFjdGVyKGFubm90LmRmWywgJ2hpc3QubmVjJ10pDQphbm5vdC5kZlssICdncm91cCddIDwtIGFzLmNoYXJhY3Rlcihhbm5vdC5kZlssICdncm91cCddKQ0Kcm93bmFtZXMoYW5ub3QuZGYpIDwtIGNlY2FsJHBpZ0lEDQphbm4uY29sb3JzIDwtIGxpc3QoaGlzdC5uZWMgPSBjKCdIZWFsdGh5JyA9IGhuX2NvbG9yc1sxXSwgJ05FQycgPSBobl9jb2xvcnNbMl0pLA0KICAgICAgICAgICAgICAgICAgIGdyb3VwID0gYygnTEFDJyA9IGRpZXRfY29sb3JzWzFdLCAnTUlYJyA9IGRpZXRfY29sb3JzWzJdLCAnQ1NTJyA9IGRpZXRfY29sb3JzWzNdKSkNCnBoZWF0bWFwKGMubWF0LA0KICAgICAgICAgY2x1c3Rlcl9yb3dzID0gVCwgDQogICAgICAgICBjbHVzdGVyX2NvbHMgPSBULA0KICAgICAgICAgY29sb3IgPSByZXYoY29sb3JSYW1wUGFsZXR0ZShicmV3ZXIucGFsKDgsICJSZEJ1IikpKDI1NikpLA0KICAgICAgICAgZm9udHNpemVfcm93ID0gNywNCiAgICAgICAgIGFubm90YXRpb24gPSBhbm5vdC5kZiwNCiAgICAgICAgIGFubm90YXRpb25fY29sb3JzID0gYW5uLmNvbG9ycywNCiAgICAgICAgIGNsdXN0ZXJpbmdfZGlzdGFuY2Vfcm93cyA9ICdldWNsaWRlYW4nLA0KICAgICAgICAgY2x1c3RlcmluZ19tZXRob2QgPSAnd2FyZC5EMicsDQogICAgICAgICBib3JkZXJfY29sb3IgPSAnZ3JheScsDQogICAgICAgICBzY2FsZSA9ICdub25lJywNCiAgICAgICAgIGdhcHNfY29sID0gbGVuZ3RoKHdoaWNoKGFubm90LmRmWywgJ2hpc3QubmVjJ10gPT0gJ0hlYWx0aHknKSksDQogICAgICAgICBzaG93X2NvbG5hbWVzID0gRiwNCiAgICAgICAgICNtYWluID0gJ0NlY2FsJywNCiAgICAgICAgIGxlZ2VuZCA9IEYsIGFubm90YXRpb25fbGVnZW5kID0gRiwNCiAgICAgICAgIGFubm90YXRpb25fbmFtZXNfY29sID0gRikNCg0Kcm0oYW5ub3QuZGYsIGMubWF0LCBhbm4uY29sb3JzLCBjLm1ldGFicy5wbG90KQ0KDQpgYGANCg0KDQpgYGB7cn0NCiMgRmlndXJlIFM5LiAgUGxhc21hIG1ldGFib2xpdGVzIGhlYXRtYXANCg0KIyBnZXQgbWV0YWJvbGl0ZXMgdG8gdXNlIGZvciBoZWF0bWFwIC0gdXNlIHAtdmFsIGN1dG9mZiBhbmQgcmFuayB0aG9zZSB3aXRoaW4NCiMgICB0aGUgY3V0b2ZmIGJ5IGRlY3JlYXNpbmcgZWZmZWN0IHNpemUsIHRoZW4gdGFrZSB0b3AgNTAgKG9yIGFsbCBpZiA8IDUwKQ0KdG1wIDwtIHAuYW5vdmFbd2hpY2gocC5hbm92YSRoaXN0Lm5lYy5wIDwgMC4wNSksIF0NCnAubWV0YWJzLnBsb3QgPC0gcm93Lm5hbWVzKHRtcFtvcmRlcihhYnMobG9nMih0bXAkbmVjLmZvbGQuZGlmZikpLCBkZWNyZWFzaW5nID0gVCksIF0pICMgYnkgZWZmZWN0IHNpemUNCnAubWV0YWJzLnBsb3QgPC0gcC5tZXRhYnMucGxvdFsxIDogaWZlbHNlKGxlbmd0aChwLm1ldGFicy5wbG90KSA8IDUwLCBsZW5ndGgocC5tZXRhYnMucGxvdCksIDUwKV0NCnJtKHRtcCkNCg0KIyBwbG90IGhlYXRtYXANCnAubWF0IDwtIHQoYXMubWF0cml4LmRhdGEuZnJhbWUocGxhc21hW29yZGVyKHBsYXNtYSRoaXN0Lm5lYywgcGxhc21hJGdyb3VwKSwgcC5tZXRhYnMucGxvdF0pKQ0KY29sbmFtZXMocC5tYXQpIDwtIHBsYXNtYVtvcmRlcihwbGFzbWEkaGlzdC5uZWMsIHBsYXNtYSRncm91cCksICdwaWdJRCddDQpyb3duYW1lcyhwLm1hdCkgPC0gcC5tZXRhYnMucGxvdA0KYW5ub3QuZGYgPC0gcGxhc21hWywgYygnaGlzdC5uZWMnLCAnZ3JvdXAnKV0NCmFubm90LmRmWywgJ2hpc3QubmVjJ10gPC0gYXMuY2hhcmFjdGVyKGFubm90LmRmWywgJ2hpc3QubmVjJ10pDQphbm5vdC5kZlssICdncm91cCddIDwtIGFzLmNoYXJhY3Rlcihhbm5vdC5kZlssICdncm91cCddKQ0Kcm93bmFtZXMoYW5ub3QuZGYpIDwtIHBsYXNtYSRwaWdJRA0KYW5uLmNvbG9ycyA8LSBsaXN0KGhpc3QubmVjID0gYygnSGVhbHRoeScgPSBobl9jb2xvcnNbMV0sICdORUMnID0gaG5fY29sb3JzWzJdKSwNCiAgICAgICAgICAgICAgICAgICBncm91cCA9IGMoJ0xBQycgPSBkaWV0X2NvbG9yc1sxXSwgJ01JWCcgPSBkaWV0X2NvbG9yc1syXSwgJ0NTUycgPSBkaWV0X2NvbG9yc1szXSkpDQpwaGVhdG1hcChwLm1hdCwNCiAgICAgICAgIGNsdXN0ZXJfcm93cyA9IFQsIA0KICAgICAgICAgY2x1c3Rlcl9jb2xzID0gVCwNCiAgICAgICAgIGNvbG9yID0gcmV2KGNvbG9yUmFtcFBhbGV0dGUoYnJld2VyLnBhbCg4LCAiUmRCdSIpKSgyNTYpKSwNCiAgICAgICAgIGZvbnRzaXplX3JvdyA9IDcsDQogICAgICAgICBhbm5vdGF0aW9uID0gYW5ub3QuZGYsDQogICAgICAgICBhbm5vdGF0aW9uX2NvbG9ycyA9IGFubi5jb2xvcnMsDQogICAgICAgICBjbHVzdGVyaW5nX2Rpc3RhbmNlX3Jvd3MgPSAnZXVjbGlkZWFuJywNCiAgICAgICAgIGNsdXN0ZXJpbmdfbWV0aG9kID0gJ3dhcmQuRDInLA0KICAgICAgICAgYm9yZGVyX2NvbG9yID0gJ2dyYXknLA0KICAgICAgICAgc2NhbGUgPSAnbm9uZScsDQogICAgICAgICBnYXBzX2NvbCA9IGxlbmd0aCh3aGljaChhbm5vdC5kZlssICdoaXN0Lm5lYyddID09ICdIZWFsdGh5JykpLA0KICAgICAgICAgc2hvd19jb2xuYW1lcyA9IEYsDQogICAgICAgICAjbWFpbiA9ICdwbGFzbWEnLA0KICAgICAgICAgbGVnZW5kID0gRiwgYW5ub3RhdGlvbl9sZWdlbmQgPSBGLA0KICAgICAgICAgYW5ub3RhdGlvbl9uYW1lc19jb2wgPSBGKQ0KDQpybShhbm5vdC5kZiwgcC5tYXQsIGFubi5jb2xvcnMsIHAubWV0YWJzLnBsb3QpDQoNCmBgYA0KDQoNCiMjIEZpZ3VyZSA2LiBTZWxlY3RlZCBwbGFzbWEgbWV0YWJvbGl0ZXMNCg0KYGBge3J9DQoNCiMgY3JlYXRlIHBsb3R0aW5nIGZ1bmN0aW9uDQpwbG90TWV0YWJvbGl0ZXMgPC0gZnVuY3Rpb24ocGxvdC5kZiwgbWV0YWJzLCBwbG90X2dyb3Vwcykgew0KICBmb3IgKHBsb3QuY21wZCBpbiBtZXRhYnMpIHsNCiAgICBpZihwbG90X2dyb3VwcyA9PSAnaGlzdC5uZWMnKSB7DQogICAgICBwbG90X2NvbG9ycyA8LSBobl9jb2xvcnMgDQogICAgfSBlbHNlIGlmKHBsb3RfZ3JvdXBzID09ICdncm91cCcpIHsNCiAgICAgIHBsb3RfY29sb3JzIDwtIGdyb3VwX2NvbG9ycw0KICAgIH0gZWxzZSB7DQogICAgICBwbG90X2NvbG9ycyA8LSBncm91cF9ORUNfY29sb3JzDQogICAgfQ0KICAgIHBsb3QgPC0gZ2dwbG90KHBsb3QuZGYsIGFlcyh4ID0gcGxvdC5kZlsgLCBwbG90X2dyb3Vwc10sIHkgPSBwbG90LmRmWywgcGxvdC5jbXBkXSwNCiAgICAgICAgICAgICAgICAgICAgICAgICAgICAgICAgY29sb3IgPSBwbG90LmRmWyAsIHBsb3RfZ3JvdXBzXSkpICsNCiAgICAgIGdlb21fYm94cGxvdChsd2QgPSAxLCBwb3NpdGlvbiA9IHBvc2l0aW9uX2RvZGdlKHdpZHRoID0gMC44NSksIHdpZHRoID0gMC43LA0KICAgICAgICAgICAgICAgICAgIG91dGxpZXIuc2hhcGUgPSBOQSkgKw0KICAgICAgZ2VvbV9wb2ludChzaGFwZSA9IDE5LCBwb3NpdGlvbiA9IHBvc2l0aW9uX2ppdHRlcmRvZGdlKGppdHRlci53aWR0aCA9IDAuMTUsDQogICAgICAgICAgICAgICAgICAgICAgICAgICAgICAgICAgICAgICAgICAgICAgICAgICAgICAgICAgICAgZG9kZ2Uud2lkdGggPSAwLjg1KSkgKw0KICAgICAgc2NhbGVfY29sb3VyX21hbnVhbCh2YWx1ZXMgPSBwbG90X2NvbG9ycykgKyB0aGVtZShsZWdlbmQucG9zaXRpb24gPSAnbm9uZScpICsNCiAgICAgIGxhYnMobGlzdCh0aXRsZSA9IHBsb3QuY21wZCwgeCA9IGVsZW1lbnRfYmxhbmsoKSwgeSA9ICdSZWxhdGl2ZSBDb25jZW50cmF0aW9uJykpICsNCiAgICAgIHRoZW1lKGF4aXMudGV4dC54ID0gZWxlbWVudF90ZXh0KGFuZ2xlID0gLTMwLCBoanVzdCA9IDApKSArIHlsaW0oLTIuNzUsIDIuNzUpDQogICAgcHJpbnQocGxvdCkNCiAgfQ0KfQ0KDQojIGFkZCBmYWN0b3IgdG8gYWxsb3cgcGxvdHRpbmcgYnkgZ3JvdXAtTkVDDQpwbGFzbWEgPC0gY2JpbmQocGxhc21hLCAnZ3JvdXAtTkVDJyA9IHBhc3RlKHBsYXNtYVssICdncm91cCddLCBwbGFzbWFbLCAnaGlzdC5uZWMnXSwgc2VwID0gJy0nKSkNCnBsYXNtYSQnZ3JvdXAtTkVDJyA8LSBmYWN0b3IocGxhc21hJCdncm91cC1ORUMnLA0KICAgICAgICAgICAgICAgICAgICAgICAgICAgICBsZXZlbHMgPSBjKCdMQUMtSGVhbHRoeScsICdMQUMtTkVDJywgJ01JWC1IZWFsdGh5JywgJ01JWC1ORUMnLA0KICAgICAgICAgICAgICAgICAgICAgICAgICAgICAgICAgICAgICAgICdDU1MtSGVhbHRoeScsICdDU1MtTkVDJykpDQoNCnBsb3QuY21wZHMgPC0gYygnbGFjdGF0ZScsICcyLXBhbG1pdG95bGdseWNlcm9waG9zcGhvZXRoYW5vbGFtaW5lJywgDQogICAgICAgICAgICAgICAgJ2FjZXR5bGNhcm5pdGluZScsICdreW51cmVuYXRlJykNCg0KIyBwbG90cyBvZiBtZXRhYm9saXRlcyB3aGljaCBkaWZmZXIgYnkgTkVDDQpwbG90TWV0YWJvbGl0ZXMocGxhc21hLCBwbG90LmNtcGRzLCAnaGlzdC5uZWMnKQ0KDQpwbG90LmNtcGRzIDwtIGMoJzIsMy1idXRhbmVkaW9sJywgJzMtaW5kb3h5bCBzdWxmYXRlJywgJ2h5b2Rlb3h5Y2hvbGF0ZScpDQoNCiMgcGxvdHMgb2YgbWV0YWJvbGl0ZXMgd2hpY2ggZGlmZmVyIGJ5IGRpZXQNCnBsb3RNZXRhYm9saXRlcyhwbGFzbWEsIHBsb3QuY21wZHMsICdncm91cCcpDQoNCnJtKHBsb3QuY21wZHMpDQoNCmBgYA0KDQojIyBGaWd1cmUgNiAoYWxzbykuIFNlbGVjdGVkIGNlY2FsIG1ldGFib2xpdGVzDQoNCmBgYHtyfQ0KDQojIGFkZCBmYWN0b3IgdG8gYWxsb3cgcGxvdHRpbmcgYnkgZ3JvdXAtTkVDDQpjZWNhbCA8LSBjYmluZChjZWNhbCwgJ2dyb3VwLU5FQycgPSBwYXN0ZShjZWNhbFssICdncm91cCddLCBjZWNhbFssICdoaXN0Lm5lYyddLCBzZXAgPSAnLScpKQ0KY2VjYWwkJ2dyb3VwLU5FQycgPC0gZmFjdG9yKGNlY2FsJCdncm91cC1ORUMnLA0KICAgICAgICAgICAgICAgICAgICAgICAgICAgIGxldmVscyA9IGMoJ0xBQy1IZWFsdGh5JywgJ0xBQy1ORUMnLCAnTUlYLUhlYWx0aHknLCAnTUlYLU5FQycsDQogICAgICAgICAgICAgICAgICAgICAgICAgICAgICAgICAgICAgICAnQ1NTLUhlYWx0aHknLCAnQ1NTLU5FQycpKQ0KDQpwbG90LmNtcGRzIDwtIGMoJ3BoZW55bGFjZXRhdGUnLCAnc2Vyb3RvbmluICg1SFQpJywgJ2RpaG9tby1saW5vbGVhdGUgKDIwOjJuNiknLA0KICAgICAgICAgICAgICAgICdreW51cmVuaW5lJykNCg0KIyBwbG90cyBvZiBtZXRhYm9saXRlcyB3aGljaCBkaWZmZXIgYnkgTkVDDQpwbG90TWV0YWJvbGl0ZXMoY2VjYWwsIHBsb3QuY21wZHMsICdoaXN0Lm5lYycpDQoNCnBsb3QuY21wZHMgPC0gYygnbGFjdGF0ZScsICdpbWlkYXpvbGUgcHJvcGlvbmF0ZScsICdwYWxtaXRveWwgZXRoYW5vbGFtaWRlJywNCiAgICAgICAgICAgICAgICAncXVpbmFsZGljIGFjaWQnLCAnMi1teXJpc3RveWxnbHljZXJvcGhvc3Bob2Nob2xpbmUnKQ0KDQpwbG90TWV0YWJvbGl0ZXMoY2VjYWwsIHBsb3QuY21wZHMsICdncm91cCcpDQoNCnJtKHBsb3QuY21wZHMpDQoNCmBgYA0KDQoNCiMjIEZpZ3VyZSBTMi4gQ3l0b2tpbmUgZXhwcmVzc2lvbg0KDQpgYGB7cn0NCnBpZ3MgPC0gZ2V0TUpOUGlncyhpbmNsLm5ldyA9IEYpDQpleHAgPSByZWFkLmNzdignRElfY3l0b2tpbmVzLmNzdicsIHN0cmlwLndoaXRlPVQpDQpleHAgPC0gbWVyZ2UocGlnc1ssIGMoJ3BpZ0lEJywgJ2dyb3VwJywgJ2hpc3QubmVjJyldLCBleHApDQoNCiMgeD1nZW5lLCBkb2RnZSBieSBncm91cHMgYW5kL29yIE5FQw0KZXhwJGdyb3VwLm5lYyA8LSBwYXN0ZShleHAkZ3JvdXAsIGV4cCRoaXN0Lm5lYywgc2VwPScuJykNCmV4cCRncm91cC5uZWMgPC0gZmFjdG9yKGV4cCRncm91cC5uZWMsDQogICAgICAgICAgICAgICAgICAgICAgICBsZXZlbHMgPSBjKCdMQUMuSGVhbHRoeScsICdMQUMuTkVDJywnTUlYLkhlYWx0aHknLCAnTUlYLk5FQycsDQogICAgICAgICAgICAgICAgICAgICAgICAgICAgICAgICAgICdDU1MuSGVhbHRoeScsICdDU1MuTkVDJyksIG9yZGVyZWQgPSBUKQ0Kc3VtX2MgPC0gc3VtbWFyeVNFKGV4cCwgbWVhc3VyZXZhciA9ICdyZWwuZXhwJywgZ3JvdXB2YXJzID0gYygnZ3JvdXAubmVjJywgJ2hpc3QubmVjJywgJ2dlbmUnKSwgbmEucm09VCkNCmdlbmVzIDwtIGxldmVscyhzdW1fYyRnZW5lKQ0KZm9yIChnIGluIGdlbmVzKSB7DQogIHBsb3QgPC0gZ2dwbG90KHN1bV9jW3N1bV9jJGdlbmUgPT0gZywgXSwgYWVzKHg9Z2VuZSwgeT1yZWwuZXhwLCBmaWxsID0gZ3JvdXAubmVjKSkgKw0KICAgIHNjYWxlX2ZpbGxfbWFudWFsKHZhbHVlcyA9IGdyb3VwX05FQ19jb2xvcnMpICsgDQogICAgc2NhbGVfY29sb3VyX21hbnVhbCh2YWx1ZXMgPSBncm91cF9ORUNfY29sb3JzKSArDQogICAgZ2VvbV9lcnJvcmJhcihhZXMoeW1pbiA9IHJlbC5leHAgLSBzZSwgeW1heCA9IHJlbC5leHAgKyBzZSksIHdpZHRoID0gMC4yLCANCiAgICAgICAgICAgICAgICAgIHBvc2l0aW9uID0gcG9zaXRpb25fZG9kZ2Uod2lkdGggPSAwLjkpKSArDQogICAgZ2VvbV9iYXIoc3RhdCA9ICdpZGVudGl0eScsIGFlcyhjb2xvdXIgPSBncm91cC5uZWMpLCBwb3NpdGlvbiA9IHBvc2l0aW9uX2RvZGdlKHdpZHRoID0gMC45KSkgKw0KICAgIGxhYnMobGlzdCh0aXRsZSA9IGVsZW1lbnRfYmxhbmsoKSwgeCA9IGVsZW1lbnRfYmxhbmsoKSwgeSA9ICdSZWxhdGl2ZSBFeHByZXNzaW9uJykpICsNCiAgICB0aGVtZShwYW5lbC5iYWNrZ3JvdW5kID0gZWxlbWVudF9ibGFuaygpLCBwYW5lbC5ncmlkLm1ham9yID0gZWxlbWVudF9ibGFuaygpLA0KICAgICAgICAgIHBhbmVsLmdyaWQubWlub3IgPSBlbGVtZW50X2JsYW5rKCksIGF4aXMubGluZSA9IGVsZW1lbnRfbGluZShjb2xvdXI9J2JsYWNrJyksDQogICAgICAgICAgYXhpcy50aXRsZS54ID0gZWxlbWVudF90ZXh0KHZqdXN0PS0wLjUpLCBheGlzLnRpdGxlLnkgPSBlbGVtZW50X3RleHQodmp1c3QgPSAxLjUpLA0KICAgICAgICAgIHRleHQgPSBlbGVtZW50X3RleHQoZmFjZSA9ICdib2xkJywgc2l6ZSA9IDE4KSwNCiAgICAgICAgICBheGlzLnRleHQgPSBlbGVtZW50X3RleHQoY29sb3IgPSAnYmxhY2snKSwNCiAgICAgICAgICBsZWdlbmQudGl0bGUgPSBlbGVtZW50X2JsYW5rKCksIGxlZ2VuZC5wb3NpdGlvbiA9ICdyaWdodCcpICsNCiAgICBndWlkZXMoY29sb3VyID0gZ3VpZGVfbGVnZW5kKG5yb3cgPSAxLCBieXJvdyA9IFQpKQ0KICBwcmludChwbG90KQ0KICBwcmludCh3aXRoKGV4cFtleHAkZ2VuZSA9PSBnLCBdLCBwYWlyd2lzZS53aWxjb3gudGVzdChyZWwuZXhwLCBncm91cC5uZWMsIHAuYWRqID0gJ2hvbG0nKSkpDQp9DQoNCnJtKHBpZ3MsIGV4cCwgc3VtX2MsIGcsIGdlbmVzLCBwbG90KQ0KDQpgYGANCg0KIyMgQ2xlYW4gdXAgYW5kIHByaW50IG91dCBzZXNzaW9uIGluZm8NCg0KYGBge3J9DQoNCnJtKGxpc3Q9bHMoKSkNCg0Kc2Vzc2lvbkluZm8oKQ0KDQpgYGA=
